# Inorganic Cobalt Sandwich Complex [(η^5^‑P_5_)Co(η^3^‑P_3_)]^−^

**DOI:** 10.1021/jacs.6c00419

**Published:** 2026-03-13

**Authors:** Karolina Trabitsch, Christoph G. P. Ziegler, Lukas Prock, Gábor Balázs, Kai Schwedtmann, Eduardo García-Padilla, Florian Meurer, Demi D. Snabilié, Bas de Bruin, Jan J. Weigand, Robert Wolf

**Affiliations:** † Institute of Inorganic Chemistry, 9147 University of Regensburg, 93040 Regensburg, Germany; ‡ Faculty of Chemistry and Food Chemistry, Dresden University of Technology, 01062 Dresden, Germany; ⊥ Van ‘t Hoff Institute for Molecular Sciences, 365304 University of Amsterdam, Science Park 904, 1098 XH Amsterdam, The Netherlands

## Abstract

Sandwich compounds are foundational to organometallic chemistry, yet carbon-free analogs remain exceptionally rare. We report the all-phosphorus heteroleptic cobalt sandwich anion [(η^5^-P_5_)­Co­(η^3^-P_3_)]^−^ (**4**), obtained via a stepwise P_4_ activation/unmasking sequence in which (nacnac′)­SiP_4_ serves as a controllable, silicon-protected P_4_ synthon. Reaction with the cobalt­(−I) complex [K­(THF)_0.2_]­[Co­(η^2^:η^2^-cod)_2_] furnishes a bis­(sila­tetra­phospha­cyclo­penta­dienyl) cobaltate anion (**1**), and successive cleavage of the Si­(nacnac′) units delivers the “naked” *cyclo*-P_5_/*cyclo*-P_3_ sandwich framework via monosilylated intermediates **2** and **3**. The cryptate salts [M­(crypt-222)]**4** (M = Na, K) were characterized by ^31^P­{^1^H} NMR spectroscopy, ESI-MS, and single-crystal X-ray diffraction (scXRD) for [Na­(crypt-222)]**4**. In solution, **4** undergoes slow disproportionation to give the paramagnetic dianion [(η^4^-P_5_)­Co­(η^3^-P_3_)]^2–^ (**5**), isolated as [K­(crypt-222)]_2_ **5** and authenticated by scXRD and EPR spectroscopy. Complexes **4** and **5** establish a deliberate strategy for accessing inorganic sandwich architectures and extend carbon-free metallocene chemistry to cobalt in two different oxidation states.

## Graphical abstract

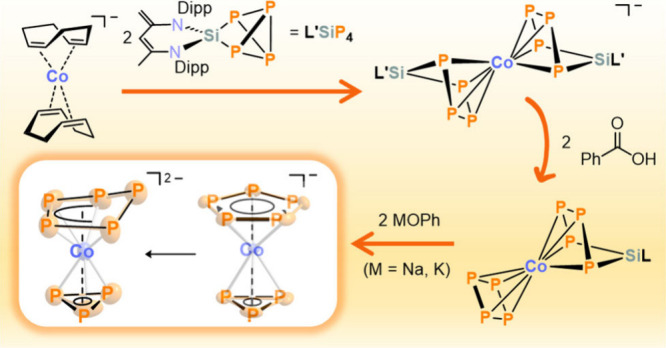

Sandwich complexes such as ferrocene, Cp_2_Fe (**A**, Cp = η^5^-C_5_H_5_), are a significant class of organometallic compounds.^1–4^ Numerous sandwich complexes have been synthesized, and metallocenes in particular have been widely used in catalysis, materials science, and medicinal chemistry.^5–9^ Recently, there has been renewed interest in their redox chemistry, leading to the discovery of rare metallocene anions and dications.^10–13^

Based on the isolobal analogy, the CH units in a Cp ligand can be formally substituted by a phosphorus atom.^14^ Baudler and co-workers demonstrated an application of this idea by synthesizing the pentaphosphacyclopentadienide (P_5_^–^) anion, a genuine phosphorus analogue of Cp^–^.^15^ The solid-state molecular structure of P_5_^–^ and its assembly on the Ag(111) surface were reported recently.^16,17^ The reaction of P_5_^–^ with FeCl_2_ in the presence of LiCp* (Cp* = C_5_Me_5_) yielded the pentaphosphaferrocene [Cp*Fe­(η^5^-P_5_)] (**B**, Chart 1), which has been applied as a building block in supramolecular chemistry.^15,18,19^

**Chart 1:**
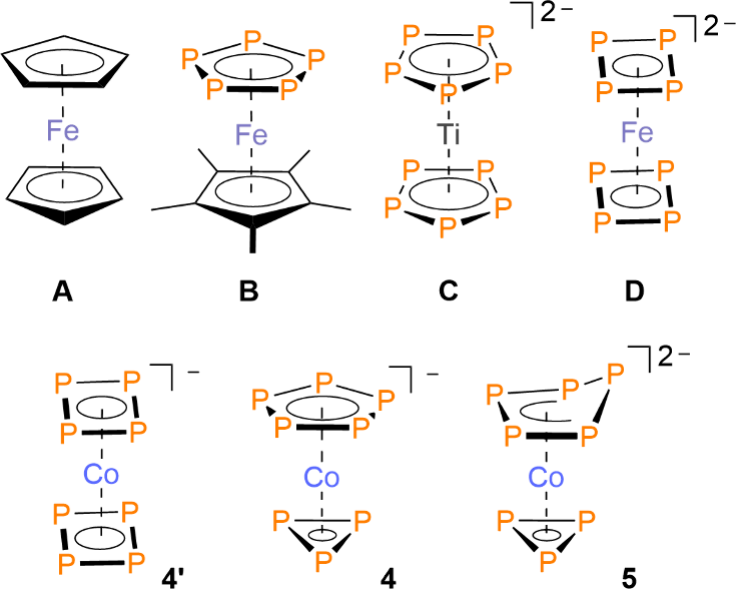
Ferrocene and Related Inorganic Sandwich Compounds

In contrast to the rich chemistry of hydrocarbon-based metallocenes, carbon-free sandwich complexes are largely unexplored, with only two examples reported to date. A landmark report by Ellis demonstrated the synthesis of the carbon-free sandwich complex [(η^5^-P_5_)_2_Ti]^2–^ (**C**, Chart 1) through the reaction of white phosphorus (P_4_) with the highly reduced titanate [Ti­(η^4^-C_10_H_8_)_3_]^2–^ (C_10_H_8_ = naphthalene).^20^ Additionally, Sun has recently reported the complex [(η^4^-P_4_)_2_Fe]^2–^ (**D**, Chart 1), which has been synthesized by the reaction of *in situ*-generated P_4_^2–^ with Fe­(O*t*Bu)_3_.^21^ Carbon-free sandwich-like compounds based on As and Sb have also been reported.^22^ Here, we describe the inorganic sandwich complex [(η^5^-P_5_)­Co­(η^3^-P_3_)]^−^ (**4**), accessed via a stepwise P_4_ activation/unmasking sequence that enables deliberate installation of “naked” *cyclo*-P_n_ decks. To our knowledge, complex **4** is the first carbon-free sandwich complex of cobalt and the first example containing two different *cyclo-*P_n_ ligands. In solution, complex **4** disproportionates slowly to generate the dianion [(η^4^-P_5_)­Co­(η^3^-P_3_)]^2–^ (**5**).

DFT calculations showed that **4** is a viable species with calculated ^31^P NMR signals at δ = 243 ppm and −211 ppm. The putative isomer [Co­(η^4^-P_4_)_2_]^−^ (**4′**) analogous to complex **D** is higher in energy by 11.5 kcal·mol^–1^. Building on previous studies of P_4_ activation by low-oxidation-state cobalt complexes,^23–26^ we initially attempted to synthesize **4** by reaction of P_4_ with cobaltate anions, such as [K([18]-crown-6)]­[Co­(η^4^-C_14_H_10_)_2_], and [K­(THF)_0.2_]­[Co­(η^2^:η^2^-1,5-cod)_2_] (C_14_H_10_ = anthracene, cod = 1,5-cyclooctadiene).^27–29^ However, these reactions did not produce **4** or any other tractable product. Given the complex reactivity of P_4_ and its well-known tendency to oligomerize,^30^ we reasoned that a stepwise activation procedure would be more suitable and devised a simple synthetic strategy based on the previously reported SiP_4_ compound (nacnac′)­SiP_4_ (nacnac′ = CH­[(C=CH_2_)­CMe]­[N­(2,6-*i*Pr_2_C_6_H_3_)]_2_), which is readily accessible from P_4_ and Driess’s silylene (nacnac′)Si (Scheme 1).^31^

**Scheme 1:**
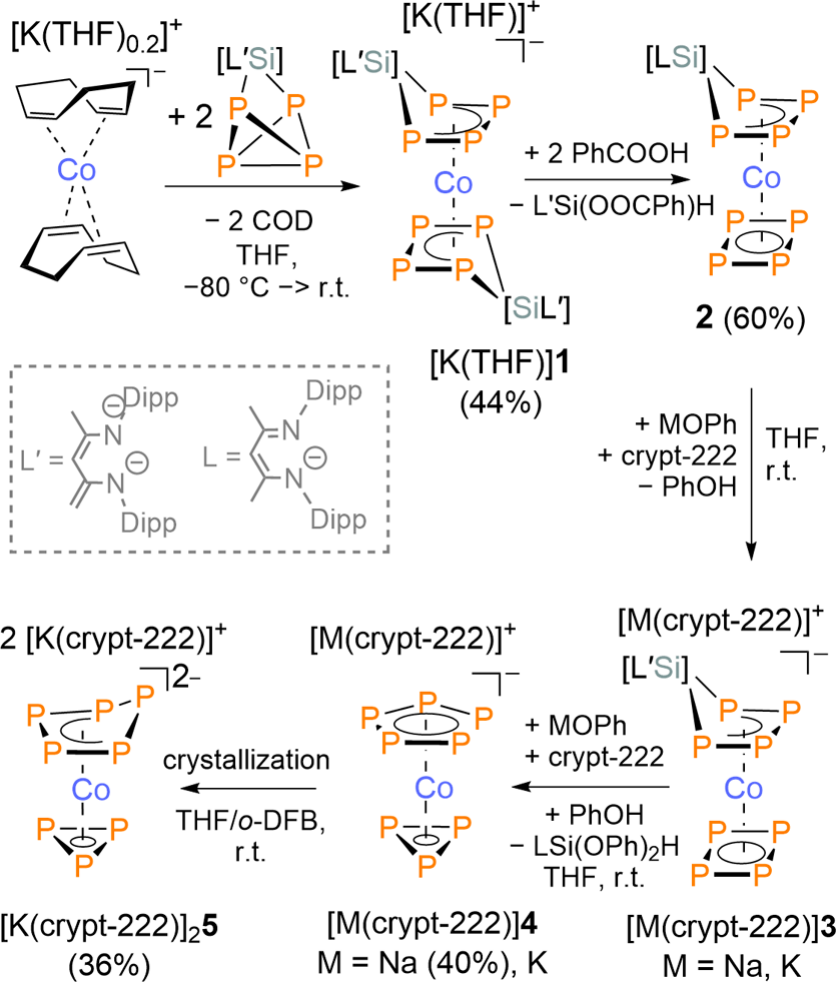
Synthesis of Complexes **1**–**5** ^a^

a Yields of isolated compounds are given in parentheses; r.t. = room temperature.

The addition of [K­(THF)_0.2_]­[Co­(η^2^:η^2^-cod)_2_] to a solution of (nacnac)­SiP_4_ in THF at low temperature (−80 °C) readily gave [K­(THF)]­[Co­{η^4^-P_4_Si­(nacnac′)}_2_] ([K­(THF)]**1**, Scheme 1) in 44% isolated yield. The ^31^P­{^1^H} NMR spectrum reveals signals corresponding to two partly overlapping AA′A″A‴XX′X″X‴ spin systems, corresponding to two isomers, which arise from different orientations of the asymmetrical nacnac′ ligands (see SI).

Crystals suitable for single-crystal X-ray diffraction (scXRD) were obtained by recrystallization of [K­(THF)]**1** from DME/*n*-hexane. The solid-state molecular structure of the resulting DME-containing salt of [K­(DME)_3_]**1** confirms that two (nacnac′)­SiP_4_ are coordinated to cobalt with the (nacnac′)Si moieties in a transoid orientation (Figure 1). As expected, the P atoms form a planar, butadiene-like arrangement.^32,33^

**Figure 1:**
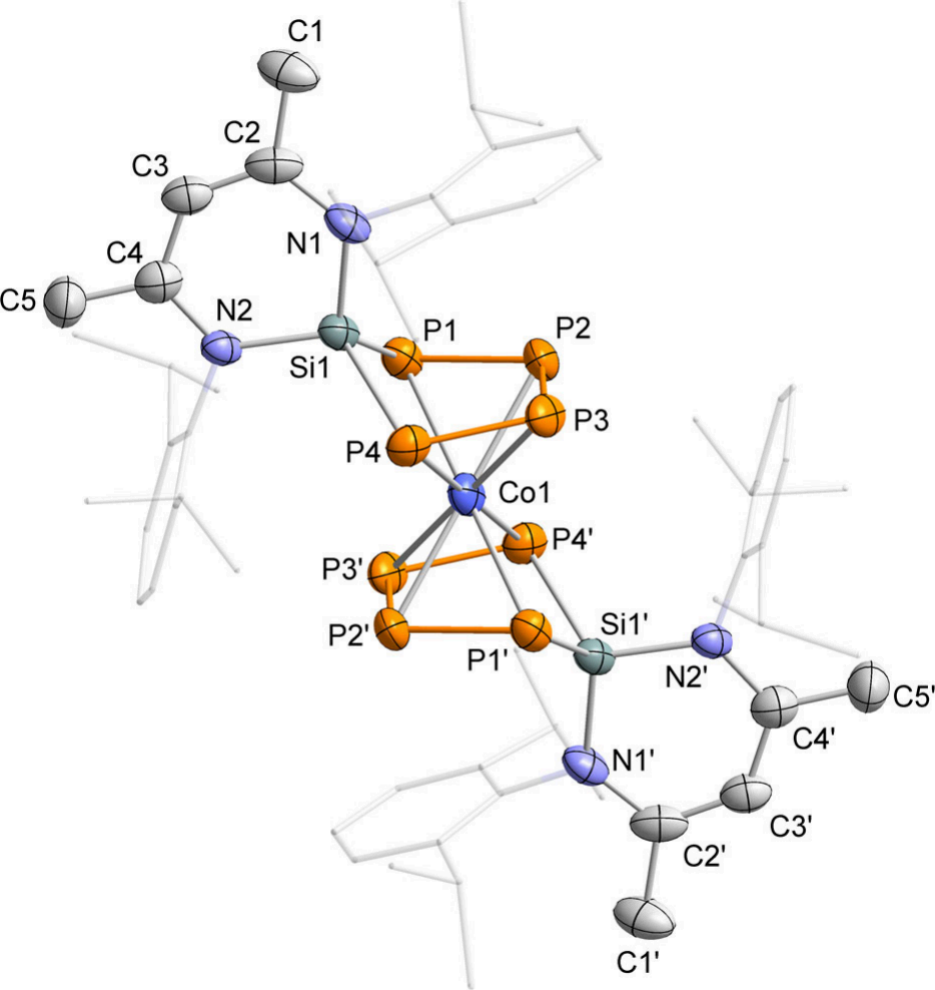
Solid-state molecular structure of [K­(DME)_3_]**1**. Hydrogen atoms, counterion, solvate molecules, and disorders are omitted for clarity. Thermal ellipsoids are drawn at the 40% probability level. The crystal contained a second, crystallographically independent molecule with similar structural parameters (not shown). Selected bond lengths [Å] and angles [°] (in case of disorder, bond lengths and angles were derived from the part with the highest occupancy): P1–P2 2.133(2), P1–P4 3.275(2), P2–P3 2.162(2), P3–P4 2.127(2), Si1–P1 2.233(2), Si1–P4 2.222(1), Co1–P1 2.404(1), Co1–P2 2.309(1), Co1–P3 2.332(1), Co1–P4 2.391(1), C1–C2 1.453(6), C4–C5 1.421(7), P1–P2–P3 105.49(7), P2–P3–P4 104.79(6), P3–P4–Si1 100.67(6), P2–P1–Si1 100.40(6), P1–Si1–P4 94.64(6).

Next, we investigated the release of one (nacnac′)Si moiety. Treatment of [K­(THF)]**1** with benzoic acid (2.0 equiv) afforded the complex [(η^4^-P_4_)­Co­{η^4^-P_4_Si­(nacnac)}] (**2**), featuring a *cyclo*-P_4_ ring and an η^4^-coordinated sila­tetra­phospha­cyclo­penta­diene ligand (Scheme 1). The side-product (nacnac′)­Si­(H)­(OOCPh) was detected by multinuclear NMR spectroscopy (see SI). Complex **2** was isolated as a brown powder by precipitation from a THF solution with Et_2_O/*n*-hexane (1:3 *v*:*v*) in 60% yield. Its identity was confirmed by multinuclear NMR and a preliminary scXRD analysis, which confirmed the atom connectivity (see SI). The ^31^P­{^1^H} NMR spectrum shows an A_4_MM′XX′ spin system with a singlet at δ­(P_A_) = 175.0 ppm corresponding to the *cyclo*-P_4_ moiety (Figure S15).

Motivated by a report by Cordaro and Grützmacher on the synthesis of cyaphide (CP^–^) complexes through the desilylation of silylphosphaalkynes R_3_SiCP with NaOPh, we attempted to remove the remaining Si­(nacnac′) unit by reaction with phenoxides MOPh.^34^ Gratifyingly, NaOPh and KOPh (2.0 equiv) selectively react with **2** in the presence of [2.2.2]-cryptand (crypt-222, 2.0 equiv) to generate the target complexes [M­(crypt-222)]­[(η^5^-P_5_)­Co­(η^3^-P_3_)] ([M­(crypt-222)]**4**, M = Na, K) and (nacnac)­SiH­(OPh)_2_ as the presumed byproduct (Scheme 1). Variable-temperature ^31^P­{^1^H} NMR reaction monitoring studies revealed that the reaction proceeds stepwise, initially forming **3**, which is identified by a similar A_4_MM′XX′ spin system as **2** with resonances at δ = 159.3 (P_A_), 108.0 (P_MM′_) and −63.7 (P_XX′_) ppm in an integral ratio of 4:2:2 (Figures S18 and S27). Compound **3** is the only detectable product in the temperature range of 193 to 273 K. At 298 K, the formation of [K­(crypt-222)]**4** becomes visible. After 3 h at room temperature, **3** is fully converted to [K­(crypt-222)]**4**. Compound **3** is the major phosphorus-containing product in the reaction of **2** with an equimolar amount of KOPh (1.0 equiv) and crypt-222 (1.0 equiv, Figure S23) and the reaction of [K­(THF)]**1** with PhOH (2.0 equiv, Figure S18).

The constitution of **4** was initially confirmed by heteronuclear NMR spectroscopy and electrospray ionization mass spectrometry (ESI-MS). ESI-MS of the reaction solution detected the molecular ions of **4** at *m*/*z* = 306.7248 in the negative ion mode and [K­(crypt-222)]^+^ at *m*/*z* = 415.2230 in the positive ion mode. The ^31^P­{^1^H} NMR spectra of [M­(crypt-222)]**4** (M = Na, K) display two resonances (Figure 2a) in a 5:3 integral ratio. The low-field resonance at δ = 239.1 ppm is assigned to the *cyclo*-P_5_ ligand. This signal appears as a singlet at room temperature and resolves into a quartet due to ^31^P–^31^P coupling with the *cyclo*-P_3_ moiety at 193 K (*J*_PP_ = 15.3 Hz). The broad singlet at δ = −225.8 ppm (Δν_1/2_ = 874 Hz) arising from the *cyclo*-P_3_ unit is consistent with the characteristically deshielded ^31^P NMR signals of end-deck *cyclo*-P_3_ transition metal complexes.^23,30^ The chemical shifts of **4** are in good agreement with those calculated by DFT (*vide supra*). Based on variable-temperature NMR studies down to 153 K, the rotation of *cyclo*-P_3_ and *cyclo*-P_5_ ligands is associated with low activation barriers, similar to those observed for the valence-isoelectronic complex [Cp‴Ni­(η^3^-P_3_)] and organic sandwich compounds (see SI).^37,38^ The broadening of the ^31^P NMR signals is due to an additional, unidentified exchange process.

**Figure 2:**
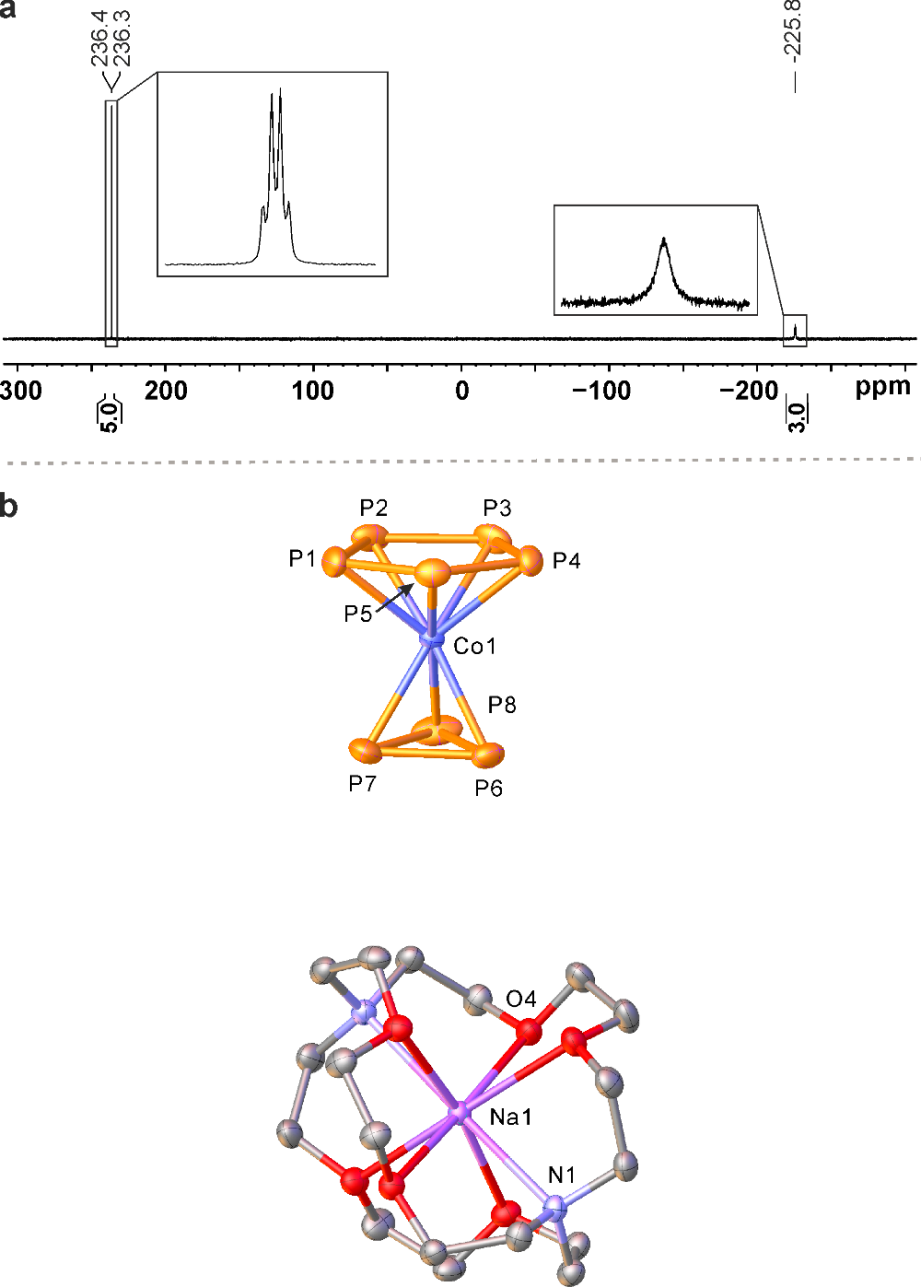
a) ^31^P­{^1^H} NMR spectra (162.04 MHz, THF-*d*_8_) of the reaction mixture of **2** with KOPh (2.0 equiv) and crypt-222 (2.0 equiv) at 193 K. b) Molecular structure of [Na­(crypt-222)]**4** with 40% probability ellipsoids. Selected bond lengths [Å]: Co1–P1 2.3120(5), Co1–P2 2.3378(5), Co1–P3 2.3283(5), Co1–P4 2.3318(5), Co1–P5 2.3690(5), Co1–P6 2.2815(5), Co1–P7 2.2781(5), Co1–P8 2.2803(5), P1–P2 2.1144(7), P1–P5 2.1077(7), P2–P3 2.1283(8), P3–P4 2.1149(7), P4–P5 2.1103(7), P6–P7 2.1382(7), P6–P8 2.1330(7), P7–P8 2.1317(8).

Crystallization of [Na­(crypt-222)]**4** and [K­(crypt-222)]**4** is hindered by the presence of nacnac-containing byproducts. However, [Na­(crypt-222)]**4** can be isolated as an orange-brown solid by filtration of a THF/toluene solution over alumina. This procedure removes byproducts and leads to the exchange of K^+^ for Na^+^ (see SI). Subsequent cooling to −35 °C yielded crystals of [Na­(crypt-222)]**4** suitable for scXRD (Figure 2b). The molecular structure shows the expected heteroleptic complex **4**, featuring a planar η^5^-*cyclo*-P_5_ ligand (P–P 2.1103(7)-2.1283(8) Å) and an η^3^-*cyclo*-P_3_ ring (P–P 2.1317(8)-2.1382(7) Å). The structure is reminiscent of [CpNi­(η^3^-C_3_Ph_3_)], a valence-isoelectronic hydrocarbon-based analogue of **4**.^35,36^ The P–P distances are typical for *cyclo*-P_3_ ligands and *cyclo*-P_5_ ligands and indicate partial multiple bond character. While several *cyclo*-P_3_ cobalt complexes are known,^23,30,39–46^ the complex [(η^5^-P_5_)­Co­{η^2^-P_2_H­(Mes)}]^2–^, prepared from K_3_P_7_ and [Co­(Mes)_2_(PEt_2_Ph)_2_] (Mes = 2,4,6-Me_3_C_6_H_2_) by Goicoechea and co-workers, appears to be the only other crystallographically characterized, mononuclear *cyclo*-P_5_ cobalt complex.^44^

A quantum crystallographic Hirshfeld atom refinement of **4** indicates a typical tetrahedrane-like bonding environment similar to that reported for [Cp‴Ni­(η^3^-P_3_)] and P_4_.^37,38^ Calculations of nucleus-independent chemical shifts and natural resonance theory suggest substantial aromatic character of the *cyclo*-P_3_ ligand (see the SI).

In solution, [K­(crypt-222)]**4** undergoes slow disproportion­ation over several days, leading to a decline in the signal-to-noise ratio in the ^31^P­{^1^H} NMR spectra (Figure S25) and resulting in the formation of the paramagnetic complex [K­(crypt-222)]_2_[(η^4^-P_5_)­Co­(η^3^-P_3_)] ([K­(crypt-222)]_2_ **5**), which was isolated in small amounts. This process additionally generates a dark brown precipitate, presumably a mixture of cobalt phosphides of unknown constitution. The nature of this solid is currently under investigation. Cyclic voltammetry shows that [Na­(crypt-222)]**4** is reduced at a cathodic peak potential of *E*_p,c_ = −2.8 V vs Fc/Fc^+^, probably to **5** (Figure S31). Crystals of [K­(crypt-222)]_2_ **5** were obtained by extraction of the crude reaction mixture of **2**, KOPh (2.0 equiv) and crypt-222 (2.0 equiv) with toluene/THF (1:1, *v*:*v*) and *o*-DFB (1,2-F_2_C_6_H_4_). An scXRD study revealed that [K­(crypt-222)]_2_ **5** crystallizes as a separated ion triple with a [(η^4^-P_5_)­Co­(η^3^-P_3_)]^2–^ dianion and two [K­(crypt-222)]^+^ cations in the asymmetric unit (Figure 3). The anion features an η^3^-coordinated *cyclo*-P_3_ ring and an envelope-shaped η^4^-ccordinated *cyclo*-P_5_ moiety. One of the P atoms (P5) is bent away from the cobalt atom. This arrangement maintains an 18-valence-electron count at cobalt.^45^ As a result, the Co–P and P–P bond lengths are similar to those in [K­(crypt-222)]**4**.

**Figure 3:**
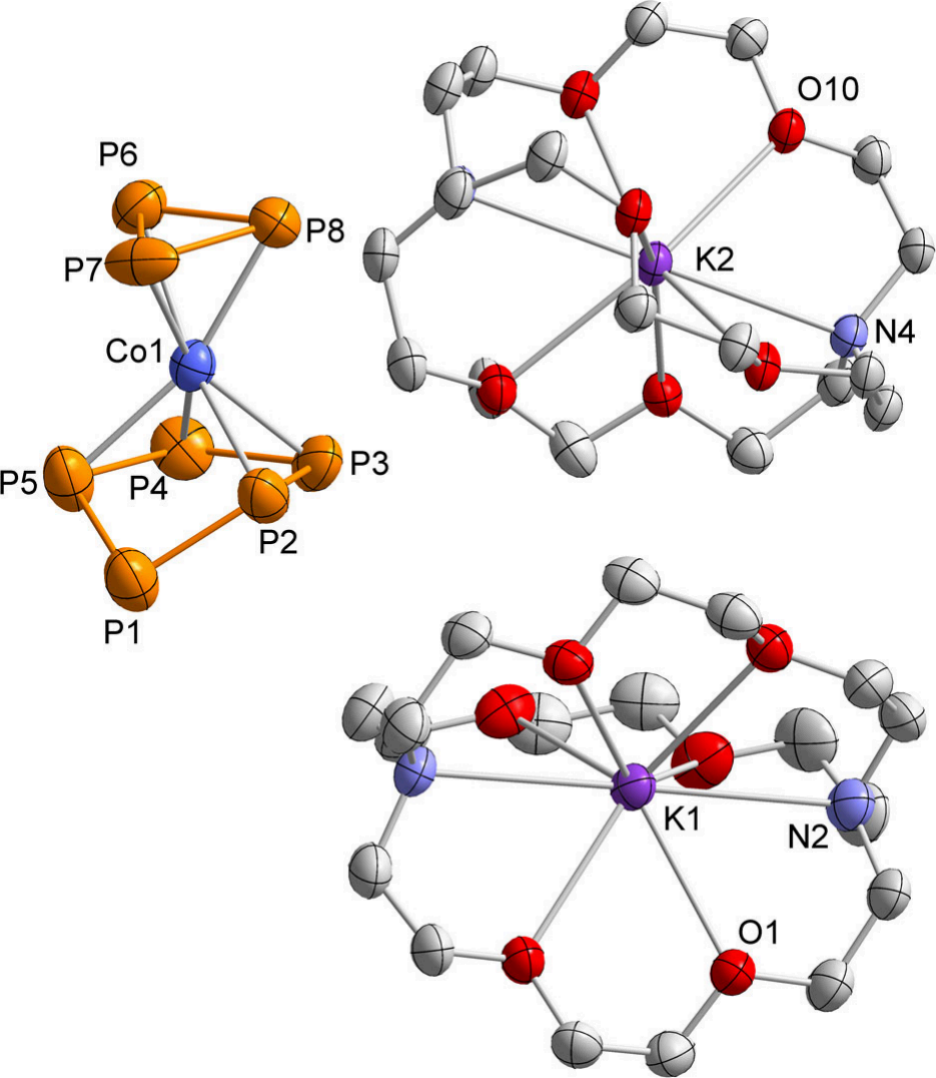
Molecular structure of [K­(crypt-222)]_2_ **5** with 40% probability ellipsoids. Hydrogen atoms are omitted for clarity. Disorder in the *cyclo*-P_3_ moiety is omitted for clarity. Selected bond lengths [Å] of the major disordered part: Co1–P2 2.298(2), Co1–P3 2.299(2), Co1–P4 2.273(2), Co1–P5 2.317(2), Co1–P6 2.309(2), Co1–P7 2.233(3), Co1–P8 2.303(3), P2–P1 2.120(3), P5–P1 2.144(3), P3–P2 2.150(3), P3–P4 2.151(3), P4–P5 2.179(3), P6–P8 2.137(3), P6–P7 2.221(4), P8–P7 2.125(4).

The composition of [K­(crypt-222)]_2_ **5** was confirmed by elemental analysis and powder X-ray diffraction of the bulk solid (see SI). Compound **5** is likely formed from the disproportionation of **4** into **5** and neutral, unidentified polyphosphorus compounds. In agreement with the expected paramagnetic behavior, an X-band EPR spectrum on a solid sample of [K­(crypt-222)]_2_ **5** at 10 K shows a nearly axial, broad signal with *g*_*iso*_ = 2.03 (Figure S28). Hyperfine couplings to the Co or P nuclei are not resolved. After dissolving a solid sample of [K­(crypt-222)]_2_ **5** in a mixture of 2-methyltetrahydrofuran and *o*-DFB, freezing the solution immediately after dissolution and recording the EPR spectrum at 10 K, a weak and broad signal can be detected, which is similar to the signal of solid [K­(crypt-222)]_2_ **5** (Figure S29). This reveals an *S* = 1/2 species consistent with the solid-state sample. DFT calculations reproduce well the experimental *g*-values and predict a rather large hyperfine coupling to Co and P. However, the magnitude of the hyperfine coupling is considerably lower than the line width (Table S3). According to the DFT calculations, the spin density in **5** is mainly located at the out-of-plane phosphorus atom and in a lesser extent on the cobalt center (Figure S30).

In conclusion, we have presented a new synthetic strategy for carbon-free transition metal polyphosphido complexes. Using the compound (nacnac′)­SiP_4_ originating from P_4_ activation and the silylene (nacnac′)­Si, this approach has led to the isolation of the remarkable complexes [K­(crypt-222)]**4** and [K­(crypt-222)]_2_ **5**, which are the first inorganic sandwich compounds isolated in two different oxidation states and with two different *cyclo*-P_n_ ligands. Notably, the stepwise “protect–activate–unmask” logic enables controlled access to heteroleptic, carbon-free sandwich architectures that are otherwise difficult to obtain from direct P_4_ activation. These results demonstrate that the stepwise activation of P_4_ constitutes a promising approach for accessing unprecedented transition-metal compounds. Efforts to synthesize additional inorganic sandwich compounds using this methodology and further investigations into the properties and reactivity of [M­(crypt-222)]**4** and [K­(crypt-222)]_2_ **5** are underway.

## Supplementary Material



## Data Availability

The NMR data for this study is available on Radar4Chem (https://radar4chem.radar-service.eu/) and can be accessed via the DOI: 10.22000/5cgrgd61z69yybj4. Computational data are available under the DOI: 10.19061/iochem-bd-1-411.

## Notes

Synthetic procedures, NMR and EPR spectroscopic data, ESI-MS and elemental analyses, crystallographic data, additional experiments, and computational details (PDF)

## §

Max-Planck-Institut für Kohlenforschung, 45470 Mülheim an der Ruhr, Germany

## Notes

After this contribution was accepted, a related manuscript describing cobalt-centered inorganic sandwich complexes was published by Sun and co-workers.^47^

## References

[1] Kealy T. J., Pauson P. L. (1951). A New Type of Organo-Iron
Compound. Nature.

[2] Goodwin C. A. P. (2024). What
Is a Sandwich Complex?. Organometallics.

[3] Long, N. J. Metallocenes: An Introduction to Sandwich Complexes; Blackwell Science Ltd, 1998.

[4] Metallocenes, Synthesis, Reactivity, Applications; Togni, A. , Halterman, R. L. , Eds.; Wiley VCH: Weinheim, 1998. 10.1002/9783527619542.

[5] Beer P. D., Hayes E. J. (2003). Transition Metal and Organometallic Anion Complexation
Agents. Coord. Chem. Rev..

[6] Dai L.-X., Tu T., You S.-L., Deng W.-P., Hou X.-L. (2003). Asymmetric Catalysis
with Chiral Ferrocene Ligands. Acc. Chem. Res..

[7] van
Staveren D. R., Metzler-Nolte N. (2004). Bioorganometallic Chemistry of Ferrocene. Chem. Rev..

[8] Astruc D. (2017). Why Is Ferrocene
so Exceptional?. Eur. J. Inorg. Chem..

[9] Patra M., Gasser G. (2017). The Medicinal Chemistry
of Ferrocene and Its Derivatives. Nat. Rev.
Chem..

[10] Malischewski M., Adelhardt M., Sutter J., Meyer K., Seppelt K. (2016). Isolation
and Structural and Electronic Characterization of Salts of the Decamethylferrocene
Dication. Science.

[11] Rall J. M., Lapersonne M., Schorpp M., Krossing I. (2023). Synthesis and Characterization
of a Stable Nickelocenium Dication Salt. Angew.
Chem., Int. Ed..

[12] Goodwin C. A. P., Giansiracusa M. J., Greer S. M., Nicholas H. M., Evans P., Vonci M., Hill S., Chilton N. F., Mills D. P. (2021). Isolation and Electronic Structures of Derivatized
Manganocene, Ferrocene and Cobaltocene Anions. Nat. Chem..

[13] Kub N. G., Sievers R., Reimann M., Streit T.-N., Steinhauer S., Schlögl J., Kaupp M., Malischewski M. (2025). Coexistence
of Metallocene Cations and Anions. J. Am. Chem.
Soc..

[14] Dillon, K. B. ; Mathey, F. ; Nixon, J. F. Phosphorus: The Carbon Copy; from Organophosphorus to Phospha-Organic Chemistry; Wiley: Chichester, 1998.

[15] Baudler M., Akpapoglou S., Ouzounis D., Wasgestian F., Meinigke B., Budzikiewicz H., Münster H. (1988). On the Pentaphosphacyclopentadienide
Ion, P_5_
^⊖^. Angew.
Chem., Int. Ed..

[16] Ernst M. J., Petrov A., Schröder M., Corzilius B., Müller C. (2025). Cyclo-P_5_
^–^ Revisited: The
Surprisingly Stable Uncoordinated Pentaphospholide Anion. Angew. Chem., Int. Ed..

[17] Chahib O., Yin Y., Liu J.-C., Li C., Glatzel T., Ding F., Yuan Q., Meyer E., Pawlak R. (2024). Probing Charge Redistribution
at the Interface of Self-Assembled Cyclo-P_5_ Pentamers on
Ag(111). Nat. Commun..

[18] Scherer O. J., Brück T. (1987). [(η^5^-P_5_)­Fe­(η^5^-C_5_Me_5_)], a Pentaphosphaferrocene
Derivative. Angew. Chem., Int. Ed..

[19] Virovets A. V., Peresypkina E., Scheer M. (2021). Structural Chemistry of Giant Metal
Based Supramolecules. Chem. Rev..

[20] Urnezius E., Brennessel W., Cramer C., Ellis J., Schleyer P. (2002). A Carbon-Free
Sandwich Complex [(P_5_)_2_Ti]^2–^. Science.

[21] Wang Z.-C., Qiao L., Sun Z.-M., Scheer M. (2022). Inorganic Ferrocene
Analogue [Fe­(P_4_)_2_]^2–^. J. Am. Chem. Soc..

[22] Yue X.-H., Chen W.-X., Yang T., Muñoz-Castro A., Frenking G., Sun Z.-M. (2023). Carbon-free sandwich compounds based
on arsenic and antimony with icosahedral metal cores. Nat. Synth..

[23] Di
Vaira M., Ghilardi C. A., Midollini S., Sacconi L. (1978). Cyclo-Triphosphorus (δ-P_3_) as a Ligand
in Cobalt and Nickel Complexes with 1,1,1-Tris­(Diphenylphosphinomethyl)­Ethane.
Formation and Structures. J. Am. Chem. Soc..

[24] Scherer O. J., Swarowsky M., Wolmershäuser G. (1989). Synthesis and Structure
of the Cobaltatetraphosphatricycloalkanes [(η^5^-C_5_Me_5_)­(CO)­CoP_4_] and [(η^5^-C_5_Me_5_)_2_(CO)_2_Co_2_P_4_]. Organometallics.

[25] Dürr S., Ertler D., Radius U. (2012). Symmetrical
P_4_ Cleavage
at Cobalt: Characterization of Intermediates on the Way from P_4_ to Coordinated P_2_ Units. Inorg. Chem..

[26] Yao S., Lindenmaier N., Xiong Y., Inoue S., Szilvási T., Adelhardt M., Sutter J., Meyer K., Driess M. (2015). A Neutral
Tetraphosphacyclobutadiene Ligand in Cobalt­(I) Complexes. Angew. Chem., Int. Ed..

[27] Jonas K., Mynott R., Krüger C., Sekutowski J. C., Tsay Y.-H. (1976). Bis­(η^4^-1,5-Cyclooctadien)­Cobaltlithium. Angew. Chem..

[28] Brennessel W. W., Young V. G., Ellis J. E. (2002). Bis­(1,2,3,4-η^4^-Anthracene)­Cobaltate­(1−). Angew.
Chem., Int. Ed..

[29] Landaeta V. R., Horsley Downie T. M., Wolf R. (2024). Low-Valent Transition Metalate Anions
in Synthesis, Small Molecule Activation, and Catalysis. Chem. Rev..

[30] Giusti L., Landaeta V. R., Vanni M., Kelly J. A., Wolf R., Caporali M. (2021). Coordination Chemistry
of Elemental Phosphorus. Coord. Chem. Rev..

[31] Xiong Y., Yao S., Brym M., Driess M. (2007). Consecutive Insertion of a Silylene
into the P_4_ Tetrahedron: Facile Access to Strained SiP_4_ and Si_2_P_4_ Cage Compounds. Angew. Chem., Int. Ed..

[32] Pelties S., Maier T., Herrmann D., de Bruin B., Rebreyend C., Gärtner S., Shenderovich I. G., Wolf R. (2017). Selective P_4_ Activation by a Highly Reduced Cobaltate: Synthesis of Dicobalt
Tetraphosphido Complexes. Chem.—Eur.
J..

[33] Yadav R., Simler T., Reichl S., Goswami B., Schoo C., Köppe R., Scheer M., Roesky P. W. (2020). Highly Selective
Substitution and Insertion Reactions of Silylenes in a Metal-Coordinated
Polyphosphide. J. Am. Chem. Soc..

[34] Cordaro J. G., Stein D., Rüegger H., Grützmacher H. (2006). Making the
True “CP” Ligand. Angew. Chem.,
Int. Ed..

[37] Meurer F., Kleemiss F., Riesinger C., Balázs G., Vuković V., Shenderovich I. G., Jelsch C., Bodensteiner M. (2024). Probing the
Isolobal Relation between Cp‴NiP_3_ and White Phosphorus
by Experimental Charge Density Analysis. Chem.—Eur.
J..

[38] Riesinger C., Meurer F., Zimmermann L., Dütsch L., Scheer M. (2025). Strain-Release Driven Arsenium Ion Bond Insertion. Angew. Chem., Int. Ed..

[35] Rausch M. D., Tuggle R. M., Weaver D. L. (1970). Formation
and Structure of a New
Mixed Sandwich Complex, (π-C_5_H_5_)­Ni­(π-C_3_Ph_3_) [π-Cyclopentadienyl-π-Triphenylcyclopropenylnickel]. J. Am. Chem. Soc..

[36] Weaver D. L., Tuggle R. M. (1971). Crystal and Molecular Structure of the Mixed Sandwich
Complex π-Cyclopentadienyl-π-Triphenylcyclopropenylnickel,
(π-C_5_H_5_)­Ni­(π-C_3_(C_6_H_5_)_3_). Inorg.
Chem..

[39] Cecconi F., Dapporto P., Midollini S., Sacconi L. (1978). Synthesis, Characterization,
and Structure of the Complex (η^3^-Cyclo-Triphosphorus)­(Tris­(2-Diphenylphosphinoethyl)­Amine)­Cobalt. Inorg. Chem..

[40] Ghilardi C. A., Midollini S., Orlandini A., Sacconi L. (1980). Single- and Mixed-Metal
Complexes with Cyclo-Triphosphorus and 1,1,1-Tris­((Diphenylphosphino)­Methyl)­Ethane,
Triphos. Synthesis and Structural Characterization of [(Triphos)­Co­(η^3^-P_3_)] and [(Triphos)­Co­(μ-(η^3^-P_3_))­{Cr_2_(CO)_10_}]. Inorg. Chem..

[41] Hoidn C. M., Maier T. M., Trabitsch K., Weigand J. J., Wolf R. (2019). [3 + 2] Fragmentation
of a Pentaphosphido Ligand by Cyanide. Angew.
Chem., Int. Ed..

[42] Piesch M., Reichl S., Seidl M., Balázs G., Scheer M. (2019). Ring Contraction by NHC-Induced Pnictogen
Abstraction. Angew. Chem., Int. Ed..

[43] Piesch M., Reichl S., Seidl M., Balázs G., Scheer M. (2021). Synthesis and Multiple Subsequent
Reactivity of Anionic *Cyclo*-E_3_ Ligand
Complexes (E = P, As). Angew. Chem., Int. Ed..

[46] Chan C., Carpenter A. E., Gembicky M., Moore C. E., Rheingold A. L., Figueroa J. S. (2019). Associative Ligand Exchange and Substrate Activation
Reactions by a Zero-Valent Cobalt Tetraisocyanide Complex. Organometallics.

[44] Knapp C. M., Westcott B. H., Raybould M. A. C., McGrady J. E., Goicoechea J. M. (2012). [Co­(η^5^-P_5_)­η^2^-P_2_H­(Mes)]^2–^: A Phospha-Organometallic
Complex Obtained by the
Transition-Metal-Mediated Activation of the Heptaphosphide Trianion. Angew. Chem., Int. Ed..

[45] Butovskiy M. V., Balázs G., Bodensteiner M., Peresypkina E. V., Virovets A. V., Sutter J., Scheer M. (2013). Ferrocene
and Pentaphosphaferrocene: A Comparative Study Regarding Redox Chemistry. Angew. Chem., Int. Ed..

[47] Wang Z.-C., Du X.-M., Wang S., Sun J.-Y., Scheer M., Sun Z.-M. (2026). Synthesis of Cobalt-Centered Inorganic Sandwich Complexes. J. Am. Chem. Soc..

