# Supplementary material for: Inorganic Cobalt Sandwich Complex [(η5‑P5)Co(η3‑P3)]−
Source: J Am Chem Soc. 2026 Mar 13;148(11):11467–72. doi: 10.1021/jacs.6c00419 (PMC13022881; doi:10.1021/jacs.6c00419)
Supplement: Supplementary file 1 [file ja6c00419_si_001.pdf]

Supporting Information

The Inorganic Cobalt Sandwich Complex $[(\eta^5\text{-}P_5)Co(\eta^3\text{-}P_3)]^-$

Karolina Trabitsch,[a‡] Christoph G. P. Ziegler,[a‡] Lukas Prock,[a] Gábor Balázs,[a] Kai Schwedtmann,[b] Eduardo García-Padilla,[a†] Florian Meurer,[a] Demi D. Snabilié,[c] Bas de Bruin,[c] Jan J. Weigand,[b]* and Robert Wolf[a]*

‡ These authors contributed equally to this manuscript.

a University of Regensburg, Institute of Inorganic Chemistry, 93040 Regensburg, Germany

b Technische Universität Dresden, Department of Chemistry and Food Chemistry, 01062 Dresden, Germany

c University of Amsterdam, Van 't Hoff Institute for Molecular Sciences), Science Park 904, 1098 XH Amsterdam, The Netherlands

*Corresponding authors e-mail: jan.weigand@tu-dresden.de, robert.wolf@ur.de

Table of Contents

1. Experimental Procedures 3

1.1 General Procedures ... 3

1.2 Synthesis of [K(THF)][Co{η^4-P_4Si(nacnac′)}$_2$] ([K(THF)]**1**) ... 5

1.3 Synthesis of [(η^4-P_4)Co{η^4-P_4Si(nacnac)}] (**2**) ... 9

1.4 Synthesis of [M(crypt-222)][(η^5-P_5)Co(η^3-P_3)] ([M(crypt-222)]**4**, M = Na, K) ... 12

1.5 Synthesis of [K(crypt-222)]$_2$[(η^4-P_5)Co(η^3-P_3)] ([K(crypt-222)]$_2$**5**) ... 16

2. Additional Experiments ... 18

2.1 Reaction of [K(THF)]**1** with two equivalents PhOH ... 18

2.2 Reaction of [(nacnac′)Si] with PhOH (2.0 equiv.) ... 21

2.3 Reaction of [K(THF)]**1** with PhCOOH (2.0 equiv.) ... 22

2.4 Reaction of [(nacnac′)Si] with PhCOOH ... 24

2.5 Reaction of [(η^4-P_4)Co{η^4-P_4Si(nacnac)}] (**2**) with KOPh (1.0 equiv.) and crypt-222 (1.0 equiv.) ... 25

2.6 Reaction of [(η^4-P_4)Co{η^4-P_4Si(nacnac)}] (**2**) with KOPh (2.0 equiv.) and crypt-222 (2.0 equiv.) ... 26

2.7 Variable-temperature ^{31}P{^{1}H} NMR reaction monitoring of the reaction of [(η^4-P_4)Co{η^4-P_4Si(nacnac)}] (**2**) with KOPh (2.0 equiv.) and crypt-222 (2.0 equiv.) ... 29

3. EPR Spectroscopic Investigations ... 30

4. Cyclic Voltammetry ... 33

5. DFT calculations ... 35

6. X-ray Crystallography ... 39

6.1 Refinement of the solid-state structure of [Na(crypt-222)][(η^5-P_5)Co(η^3-P_3)] ([Na(crypt-222)]**4**) ... 39

6.2 Refinement of the solid-state molecular structure of [K(crypt-222)]$_2$[(η^4-P_5)Co(η^3-P_3)] ([K(crypt-222)]$_2$**5**) ... 40

6.3 Refinement of the solid-state molecular structure of [K(DME)$_3$][Co{η^4-P_4Si(nacnac′)}$_2$] ([K(DME)$_3$]**1**) ... 42

6.4 Refinement of the solid-state structure of [(η^4-P_4)Co{η^4-P_4Si(nacnac)}] (**2**) ... 43

7. References ... 46

1. Experimental Procedures

1.1 General Procedures

All manipulations were performed under an atmosphere of dry argon using standard Schlenk techniques or a MBraun UniLab glovebox (maintained at <0.1 ppm H_2O and <0.1 ppm O_2). Solvents were dried and degassed with an MBraun SPS800 solvent-purification system. Diethyl ether, toluene and THF were stored over molecular sieves (3 Å). *n*-Hexane was stored over a potassium mirror. 1,2-dimethoxyethane (DME) was stirred over K/benzophenone, distilled, and stored over molecular sieves (3 Å). 1,2-*ortho*-difluorobenzene (*o*-DFB) was stirred over Na/benzophenone, distilled, and stored over molecular sieves. *n*-Pentane and 1,4-dioxane were stirred over sodium, distilled, and stored over a potassium mirror. MeCN was stirred over CaH_2, distilled, and stored over molecular sieves (3 Å). Deuterated solvents (C_6D_6, tol-d_8, THF-d_8, MeCN-d_3, DMF-d_7) were stirred over potassium, distilled, degassed, and stored over molecular sieves (3 Å). The starting materials [K(thf)$_{0.2}$][Co(η^4-1,5-cod)$_2$][1] and [(nacnac′)Si(P_4)],[2,3] were prepared according to previously reported procedures. Benzoic acid, crypt-222 and LiOPh were purchased from Sigma Aldrich and used as delivered.

NMR Spectroscopy: NMR spectra were measured on a Bruker AVANCE III HD Nanobay [^{1}H (400.13 MHz), ^{13}C (100.61 MHz), ^{29}Si (79.50 MHz), ^{31}P (161.98 MHz)] 400 MHz UltraShield or on a Bruker AVANCE III HDX, 500 MHz Ascend [^{1}H (500.13 MHz), ^{13}C (125.75 MHz), ^{19}F (470.59 MHz), ^{29}Si (99.36 MHz), ^{31}P (202.45 MHz)]. All ^{13}C NMR spectra were exclusively recorded with composite pulse decoupling. Reported numbers assigning atoms in the ^{13}C spectra were indirectly deduced from the cross-peaks in 2D correlation experiments (HMBC, HSQC). Chemical shifts were referenced to δ_{TMS} = 0.00 ppm (^{1}H, ^{13}C), δ_{CFCl3} = 0.00 ppm (^{19}F), δ_{TMS} = 0.00 ppm (^{29}Si) and $\delta_{H3PO4(85\%)}$ = 0.00 ppm (^{31}P). Chemical shifts (δ) are reported in ppm. Coupling constants (J) are reported in Hz.

For compounds which give rise to a higher order spin system in the ^{31}P{^{1}H} NMR spectrum, the resolution-enhanced ^{31}P{^{1}H} NMR spectrum was transferred to the software gNMR, version 5.0, by Cherwell Scientific.[4] The full line shape iteration procedure of gNMR was applied to obtain the best match of the fitted to the experimental spectrum. $^1J(^{31}P^{31}P)$ coupling constants were set to negative values and all other signs of the coupling constants were obtained accordingly. The primary NMR data for this study is available on Radar4Chem and can be accessed *via* DOI: 10.22000/5cgrgd61z69yybj4.

Elemental analysis: Elemental analyses were determined by the analytical department of the University of Regensburg with a Micro Vario Cube (Elementar) elemental analyzer or by the Microanalytical Laboratory Kolbe in Oberhausen.

Mass spectrometry: The ESI-MS spectra were recorded by the analytical department of the University of Regensburg with an Agilent Q-TOF 6540 UHD mass spectrometer.

EPR spectroscopy: EPR spectra were recorded on a Bruker EMX Plus X-band spectrometer equipped with an ER 4112HV-CF100 He cryostat. MeTHF and *o*-DFB were dried over basic alumina and degassed by three freeze-pump-thaw cycles.

Powder X-ray diffraction: Samples were prepared in sealed capillaries (Ø 0.3 mm, mark tube). The data was collected on a STOE STADI P diffractometer (STOE, Darmstadt, Germany) equipped with a Dectris Mythen 1K detector using monochromatic CuKα radiation

(1.54060 Å). The refinement was carried out using the software WinXPow - Powder Diffraction Software, Version 3.10, STOE&Cie GmbH, Darmstadt, Germany, 2016.

1.2 Synthesis of [K(THF)][Co{η^4-P_4Si(nacnac′)}$_2$] ([K(THF)]1)

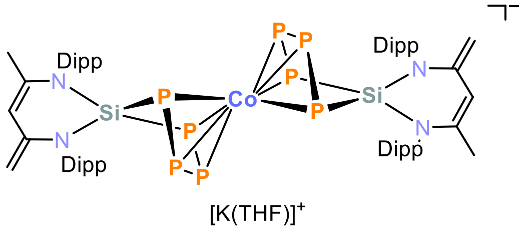

A yellow solution of [K(THF)$_{0.2}$][Co(η^4-1,5-cod)$_2$] (433.7 mg, 1.32 mmol, 1.0 equiv.) in THF (30 mL) cooled to −80 °C was added dropwise to a precooled (−80 °C) yellow solution of [(nacnac′)SiP_4] (1.50 g, 2.64 mmol, 2.0 equiv.) in THF (50 mL). The reaction mixture was stirred for 3 h at −80 °C. Subsequently, the reaction mixture was left in the cooling bath and allowed to warm to room temperature over 16 h. The resulting brownish-yellow solution was filtered through a P4 frit, concentrated in *vacuo* and layered with with *n*-hexane. Storage of the solution at room temperature gave brown block-shaped crystals of [K(THF)]**1**. The ^{1}H NMR spectrum of the isolated product contains 1.0 THF molecules per formula unit after drying in *vacuo* (10^{-3} mbar) for 5 h. Multinuclear NMR spectra reveal the presence of two diastereomers of [K(THF)]**1** (labeled isomer **A** and **B**), which are present in a 1:1 ratio. Yield: 760 mg (44%).

^{1}H NMR (400.13 MHz, 300 K, THF-*d*$_8$): δ / ppm = 0.95 (m, 12 H, Dipp: CH*Me*$_2$), 1.18–1.24 (m, 14 H, Dipp: Dipp: CH*Me*$_2$ overlapping with ^{Dipp}nacnac′: NC*Me*), 1.30-1.34 (m, 12H, Dipp: CH*Me*$_2$ overlapping with ^{Dipp}nacnac′: NC*Me*), 1.46-1.61 (m, 16H, Dipp: CH*Me*$_2$ overlapping with ^{Dipp}nacnac′: NC*Me*), 2.35 (s, 1 H, ^{Dipp}nacnac′: NCC*H*$_2$), 2.69-2.82 (m, 3 H, Dipp: C*H*Me$_2$ overlapping with ^{Dipp}nacnac′: NCC*H*$_2$), 2.87-2.96 (m, 2 H, Dipp: C*H*Me$_2$), 3.19 (s, 1 H, ^{Dipp}nacnac′: NCC*H*$_2$), 3.42 (s, 1 H, ^{Dipp}nacnac′: NCC*H*$_2$), 3.71-3.85 (m, 4 H, Dipp: C*H*Me$_2$), 4.87-4.92 (m, 2 H, ^{Dipp}nacnac′: γ-C*H*), 6.88-6.92 (m, 2 H, Dipp: 2,6-*i*Pr$_2$C$_6$*H*$_3$), 6.99-7.12 (m, 4 H, Dipp: 2,6-*i*Pr$_2$C$_6$*H*$_3$), 7.16-7.35 (m, 6 H, Dipp: 2,6-*i*Pr$_2$C$_6$*H*$_3$).

^{13}C{^{1}H} NMR (100.61 MHz, 300 K, THF-*d*$_8$): δ / ppm = 22.9–29.6 (Dipp: CH*Me*$_2$, ^{Dipp}nacnac′: NC*Me*), 85.0, 85.1, 85.3, 85.5 (4 signals, 2 for each isomer, ^{Dipp}nacnac′: NC*C*H$_2$), 103.4, 103.5, 103.6, 104.2 (4 signals, 2 for each isomer, ^{Dipp}nacnac′: γ-*C*H), 124.6–127.8 (Dipp: *C*HMe$_2$), 139.2–148.8 (Dipp: *i*Pr$_2$*C*$_6$H$_3$, ^{Dipp}nacnac′: N*C*Me), 148.3, 148.4, 149.7, 149.8 (4 signals, 2 for each isomer, ^{Dipp}nacnac′: N*C*CH$_2$).

^{31}P{^{1}H} NMR (161.98 MHz, 300 K, THF-*d*$_8$): δ / ppm = –77.8 (m, 4 P; isomer **A** and **B**), 127.7 (m, 2 P; isomer **A**), 140.8 (m, 2 P; isomer **B**).

^{29}Si NMR (99.36 MHz, 300 K, THF-*d*$_8$): δ / ppm = 19.5 (t, $^1J(^{31}P^{29}Si)$ = 112 Hz, isomer **A**), 16.0 (t, $^1J(^{31}P^{29}Si)$ = 107 Hz, isomer **B**), 25.7 (t, $^1J(^{31}P^{29}Si)$ = 119 Hz, isomer **A**).

Elemental analysis (%) calcd. for $C_{62}H_{68}CoKN_4OP_8Si_2$ (Mw = 1307.40 g·mol^{-1}): C 56.96, H 6.48, N 4.20; found C 56.95, H 6.78, N 4.20.

The possible stereoisomers of [K(THF)]**1** are shown in Figure S1.

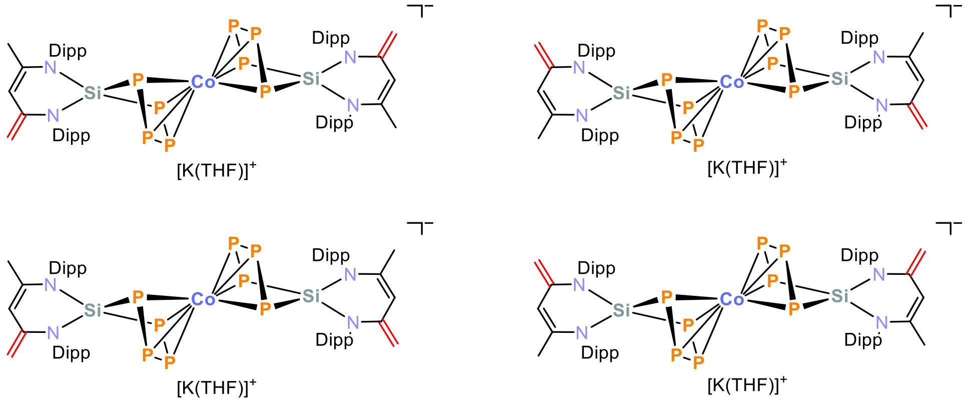

Figure S1. Conceivable stereoisomers of [K(THF)]**1**.

The presence of the dianionic nacnac′ ligands is confirmed by ^{1}H NMR spectra of the isolated complex [K(THF)]**1**, showing four singlet resonances arising from the magnetically inequivalent H-atoms of the exocyclic CH_2 groups in the two β-diketiminate backbones (Figure S2). For the γ-H atoms of the nacnac′ backbone, three singlet resonances were detected, indicating the presence of three magnetically inequivalent nacnac′ ligands. The $C{=}CH_2$ double bonds can be oriented *syn* and *anti* with respect to the central P_4CoP_4 core, as well as in *endo* or *exo* position with respect to the SiP_4 unit leading to different isomers. This was also found in an unusual $Ni_2Si_2P_8$ cluster capped by two [Si(nacnac′)] fragments.[3] The ^{29}Si NMR spectrum of [K(THF)]**1** displays three triplet resonances with a 1:2:1 integral ratio (Figure S5). The $^{31}P\{^{1}H\}$ NMR spectrum of [K(THF)]**1** reveals three multiplets of two, partly overlapping AA'A"A'"XX'X"X'" spin systems in a 2:2:4 integral ratio (Figure S4). The correct elemental analysis (*vide supra*) of the isolated solid proves that the species in solution have the same elemental composition.

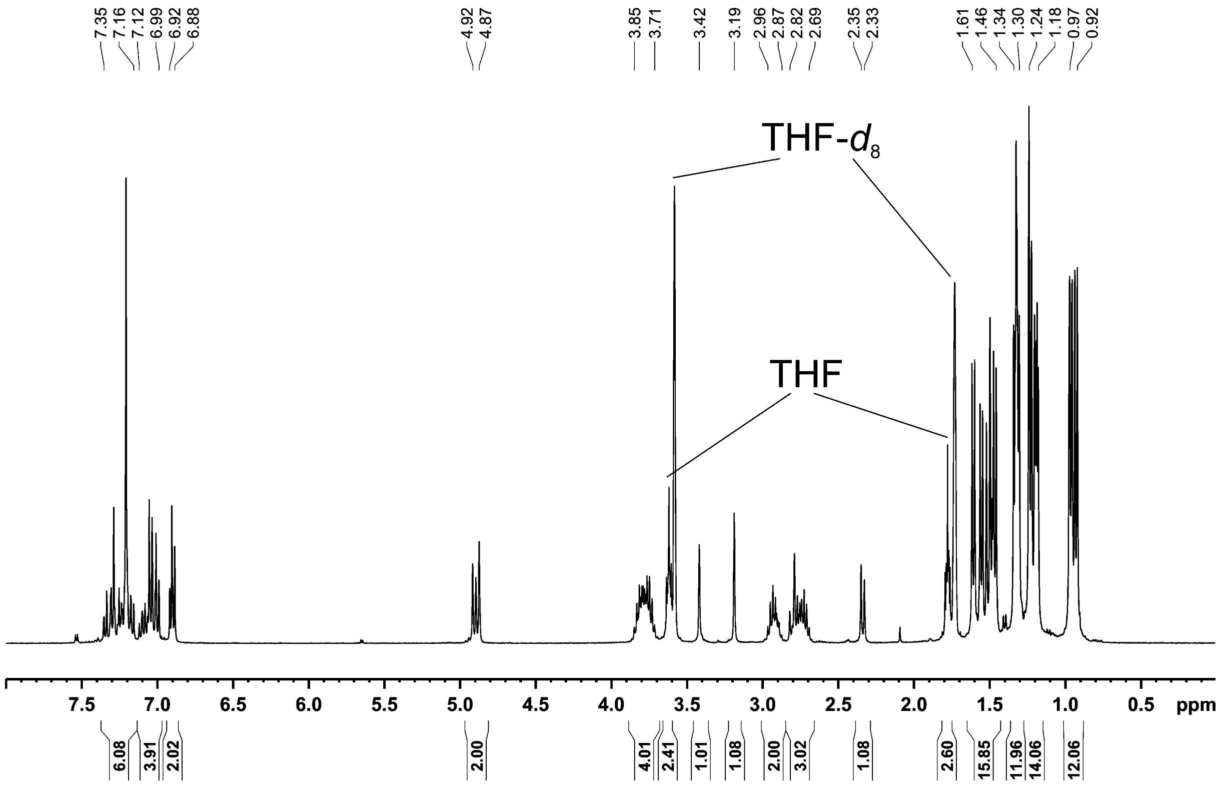

Figure S2. ^{1}H NMR spectrum (400 MHz, 300 K, THF-d_8) of [K(THF)][Co{η[4]-P_4Si(nacnac′)}$_2$] ([K(THF)]**1**).

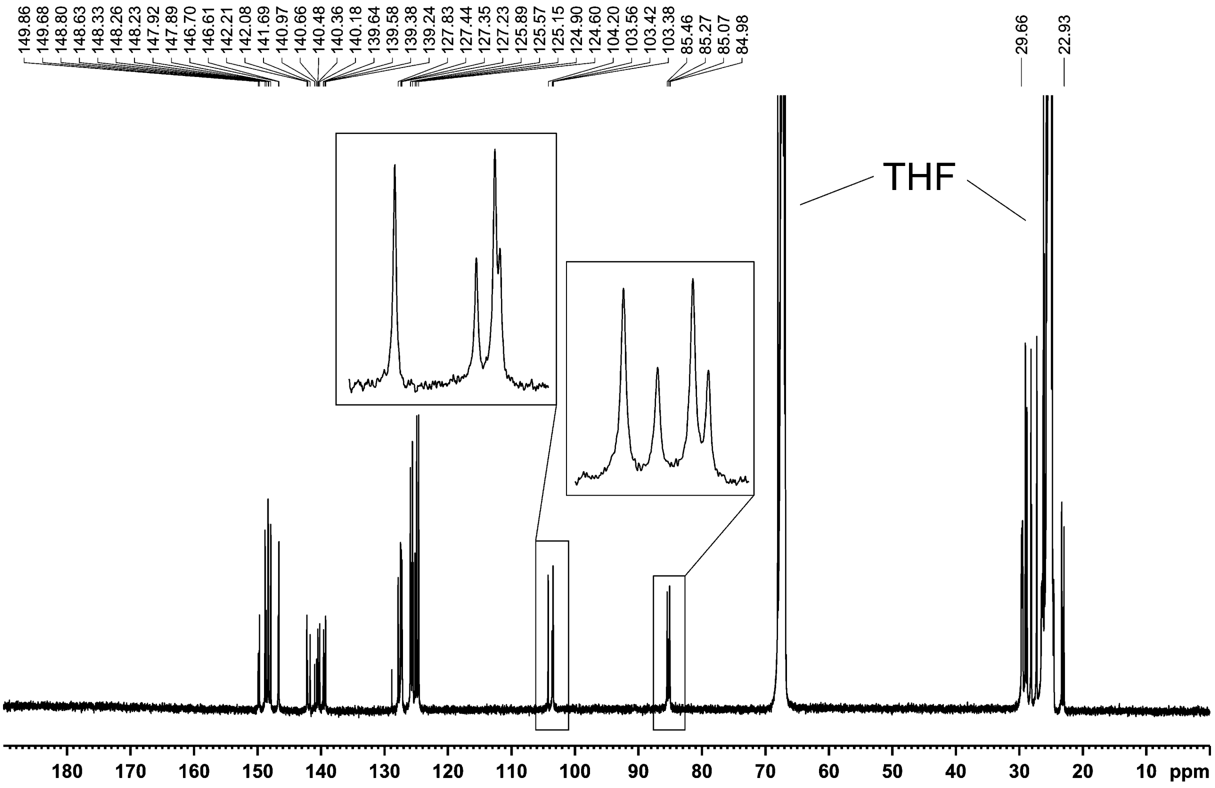

Figure S3. ^{13}C{^{1}H} NMR spectrum (100.61 MHz, 300 K, THF-d_8) of [K(THF)][Co{η[4]-P_4Si(nacnac′)}$_2$] ([K(THF)]**1**).

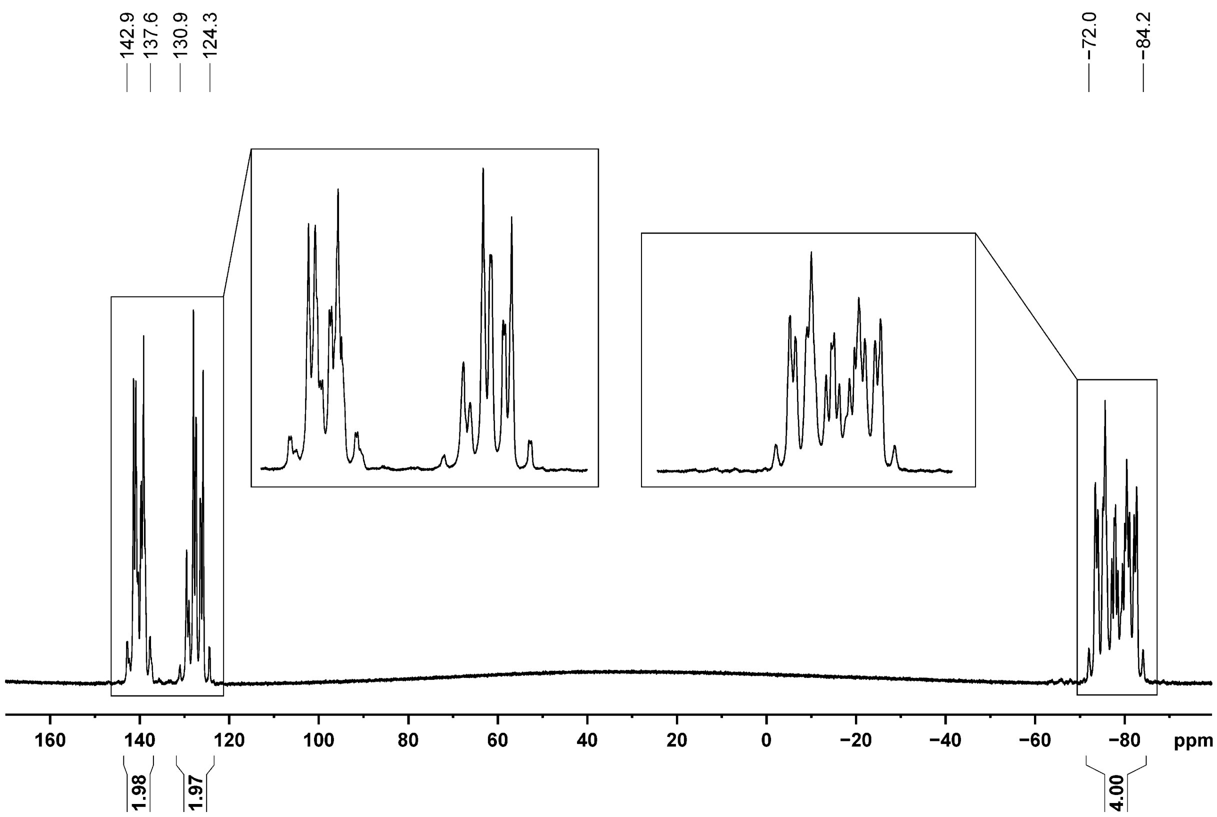

Figure S4. $^{31}P\{^{1}H\}$ NMR spectrum (161.98 MHz, 300 K, THF-d_8) of [K(THF)][Co{η^4-P_4Si(nacnac′)}$_2$] ([K(THF)]**1**).

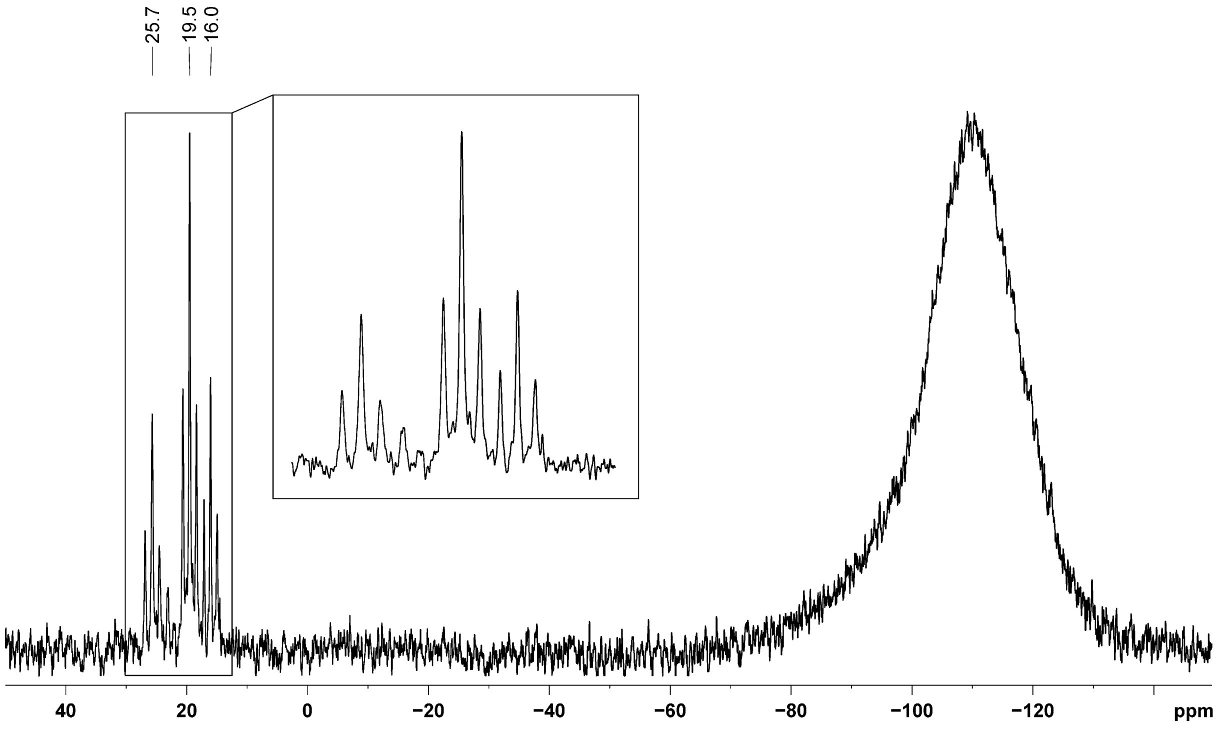

Figure S5. ^{29}Si NMR spectrum (99.36 MHz, 300 K, THF-d_8) of [K(THF)][Co{η^4-P_4Si(nacnac′)}$_2$] ([K(THF)]**1**).

1.3 Synthesis of [(η^4-P_4)Co{η^4-P_4Si(nacnac)}] (2)

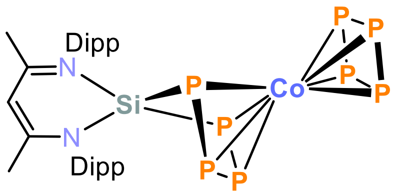

A colorless THF solution (5 mL) of benzoic acid (45.4 mg, 0.372 mmol, 2.0 equiv.) was added to a brownish-yellow solution of [K(THF)]**1** (230 mg, 0.186 mmol, 1.0 equiv.) in THF (10 mL) at room temperature. The mixture was stirred for 3 h. Subsequently, diethyl ether (20 mL) and *n*-hexane (60 mL) were added while stirring, whereupon a brown powder precipitated. The precipitate was collected on a P4 frit. The crude product was washed with a mixture of Et_2O/*n*-hexane (10 mL:20 mL *v*:*v*) and with a mixture of Et_2O/*n*-hexane (20 mL:20 mL *v*:*v*). The brown solid was dried in *vacuo* (10^{-3} mbar) for 3 h. ^{1}H NMR spectrum of the isolated product contains 0.21 Et_2O and 0.17 *n*-hexane solvate molecules per formula unit. Yield: 88 mg (60%).

Plate-like orange single crystals of the CH_2Cl_2 solvate **2**·2CH_2Cl_2 suitable for XRD analysis were obtained by storing a CH_2Cl_2 solution layered with *n*-hexane at −35°C. The CH_2Cl_2 solvate molecules can be removed by drying the crystals under vacuum (10^{-3} mbar). Note that **2** is unstable in CH_2Cl_2, showing significant decomposition after 1 d at room temperature.

^{1}H NMR (400.13 MHz, 300 K, THF-d_8): δ / ppm = 1.09 (d, $^3J_{HH}$ = 6.7 Hz, 6 H, Dipp: CH*Me*$_2$), 1.26 (d, $^3J_{HH}$ = 6.7 Hz, 6 H, Dipp: CH*Me*$_2$), 1.47 (d, $^3J_{HH}$ = 6.7 Hz, 6 H, Dipp: CH*Me*$_2$), 1.67 (d, $^3J_{HH}$ = 6.7 Hz, 6 H, Dipp: CH*Me*$_2$), 1.99 (s, 3 H, ^{Dipp}nacnac: NCCH_3), 2.17 (s, 3 H, ^{Dipp}nacnac: NCCH_3), 2.90 (sept, $^3J_{HH}$ = 6.7 Hz, 2 H, Dipp: C*H*Me$_2$), 3.70 (sept, $^3J_{HH}$ = 6.7 Hz, 4 H, Dipp: C*H*Me$_2$), 5.87 (s, 1 H, ^{Dipp}nacnac: γ-C*H*), 7.35 – 7.54 (m, 6 H, Dipp: 2,6-*i*Pr$_2$C$_6$*H*$_3$).

^{13}C{^{1}H} NMR (100.61 MHz, 300 K, THF-d_8): δ / ppm = 25.1 (Dipp: CH*Me*$_2$), 25.1 (^{Dipp}nacnac: NCCH_3), 25.3 (^{Dipp}nacnac: NCCH_3), 25.6 (Dipp: CH*Me*$_2$), 25.9 (Dipp: CH*Me*$_2$), 26.2 (Dipp: CH*Me*$_2$), 102.0 (^{Dipp}nacnac: γ-*C*H), 123.6 (Dipp, *C*H), 125.9 (Dipp), 126.9 (Dipp, *C*H), 127.1 (Dipp, *C*H), 130.7(Dipp, *C*H), 130.9 (Dipp, *C*H), 136.0 (Dipp), 138.6 (Dipp), 145.1 (Dipp,), 146.1 (Dipp,), 170.1 (^{Dipp}nacnac: N*C*CH$_2$), 174.9 (^{Dipp}nacnac: N*C*CH$_2$).

^{31}P{^{1}H} NMR (161.98 MHz, 300 K, THF-d_8, AMM'XX' spin system): δ / ppm = 175.0 (s, 4P, P_A), 111.3 (m, 2P, $P_{MM'}$), –86.0 (m, 2P, $P_{XX'}$), for parameters obtained by simulation, see Table S1.

^{29}Si NMR (79.49 MHz, 300 K, THF-d_8): δ / ppm = 23.4 (t, br, ^1J(P,Si) = 144 Hz).

Elemental analysis calcd. for $C_{29}H_{41}CoN_2P_8Si$ · 0.21 $C_4H_{10}O$ · 0.17 C_6H_{14} (Mw = 782.69 g·mol^{-1}): C 47.36, H 5.86, N 3.58; found C 48.06, H 4.98, N 2.80. The small deviation in the CHN analysis is attributed to minor impurities, presumably nacnac-derived species, which are also observed in the ^{1}H NMR spectrum (Figure S6).

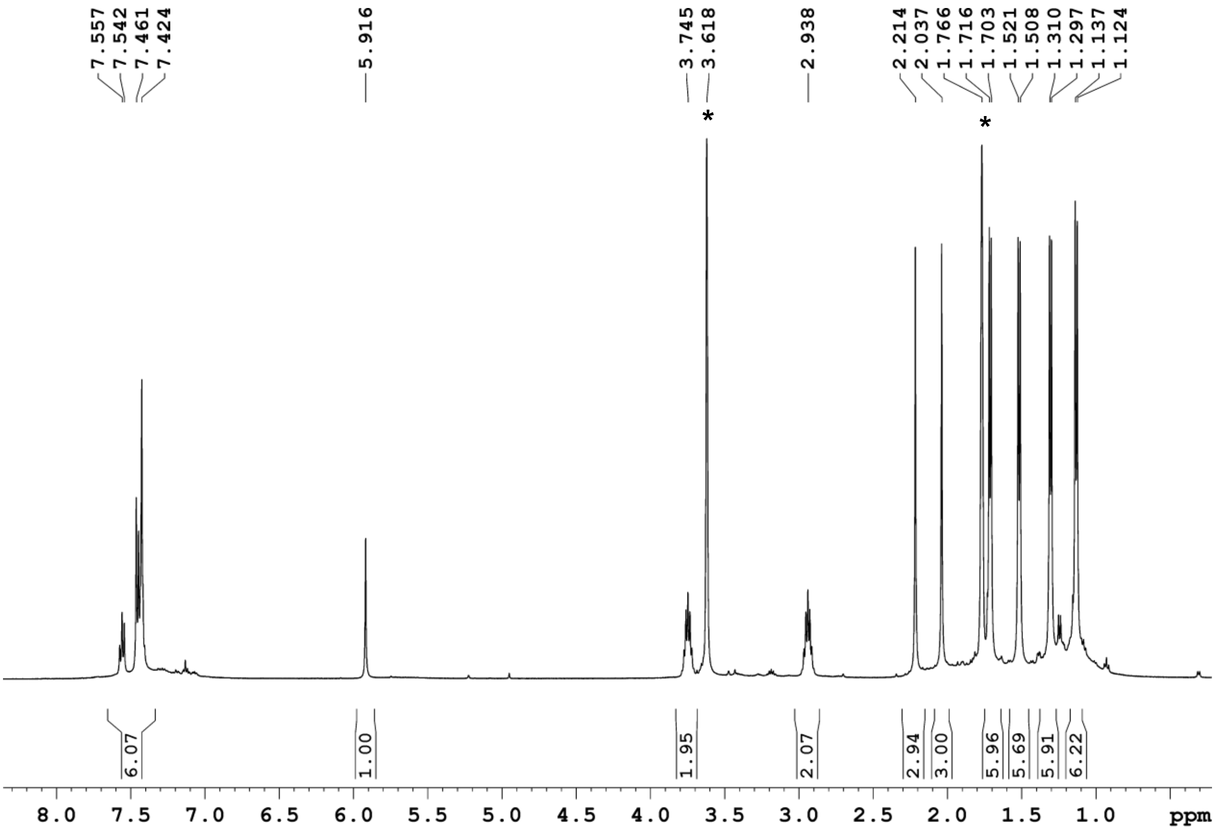

Figure S6. ^{1}H NMR spectrum (500 MHz, 300 K, THF-d_8) of [(η^4-P_4)Co{η^4-P_4Si(nacnac)}] (**2**).
* = residual solvent (THF).

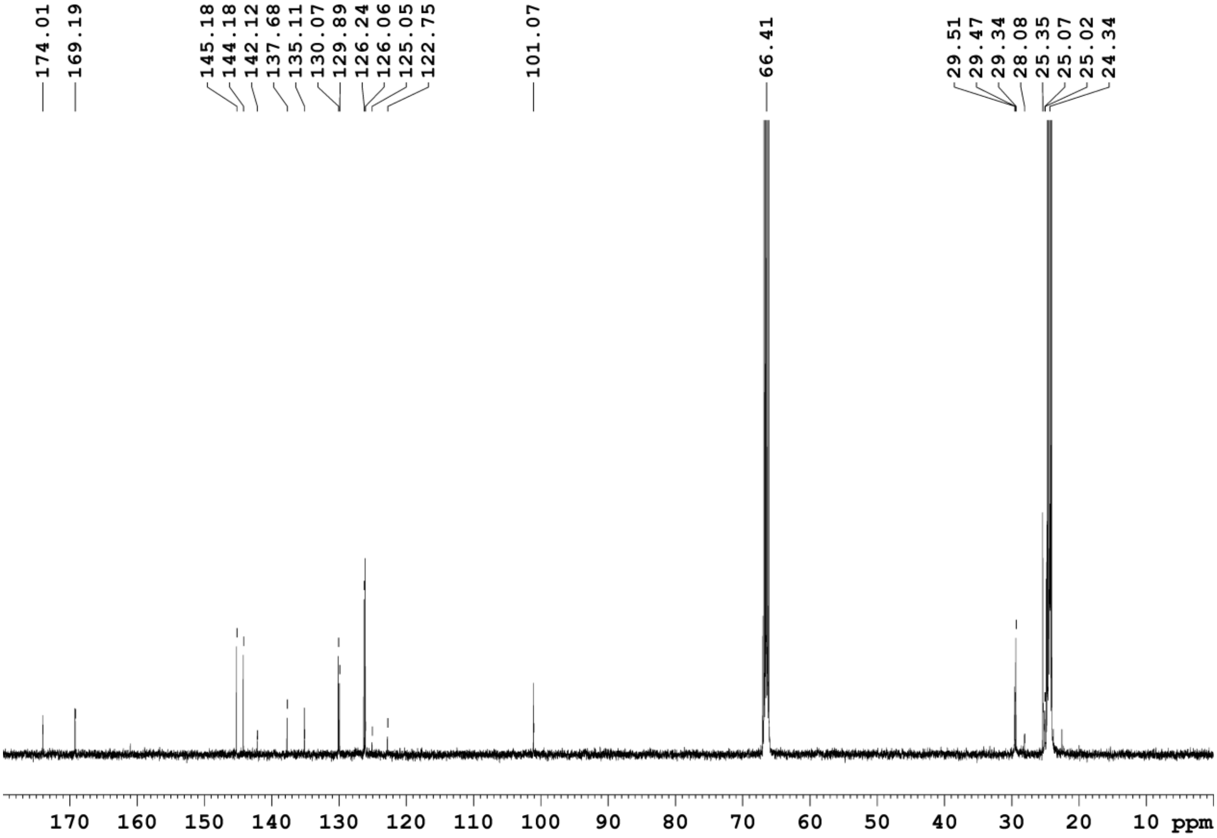

Figure S7. ^{13}C{^{1}H} NMR spectrum (125.78 MHz, 300 K, THF-d_8) of [(η^4-P_4)Co{η^4-P_4Si(nacnac)}] (**2**).

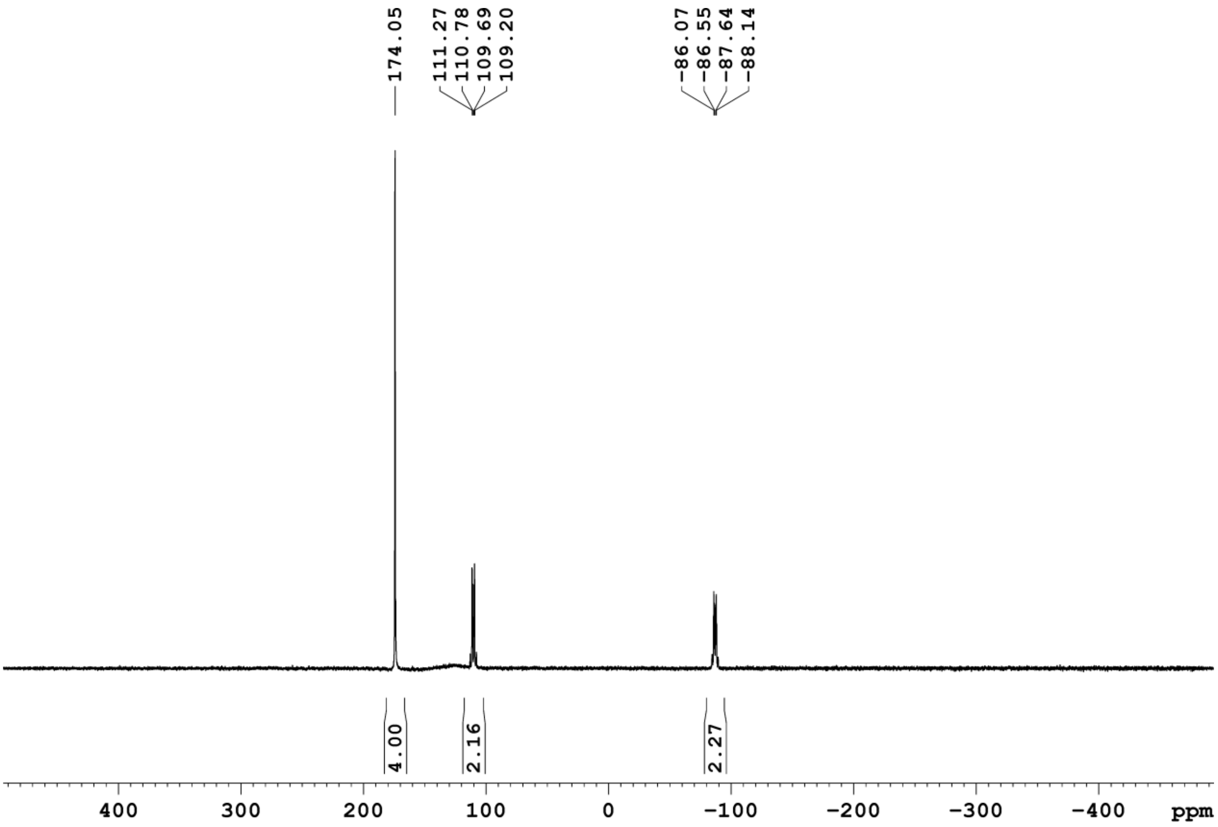

Figure S8. $^{31}P\{^{1}H\}$ NMR spectrum (202.48 MHz, 300 K, THF-d_8) of [(η^4-P_4)Co{η^4-P_4Si(nacnac)}] (**2**).

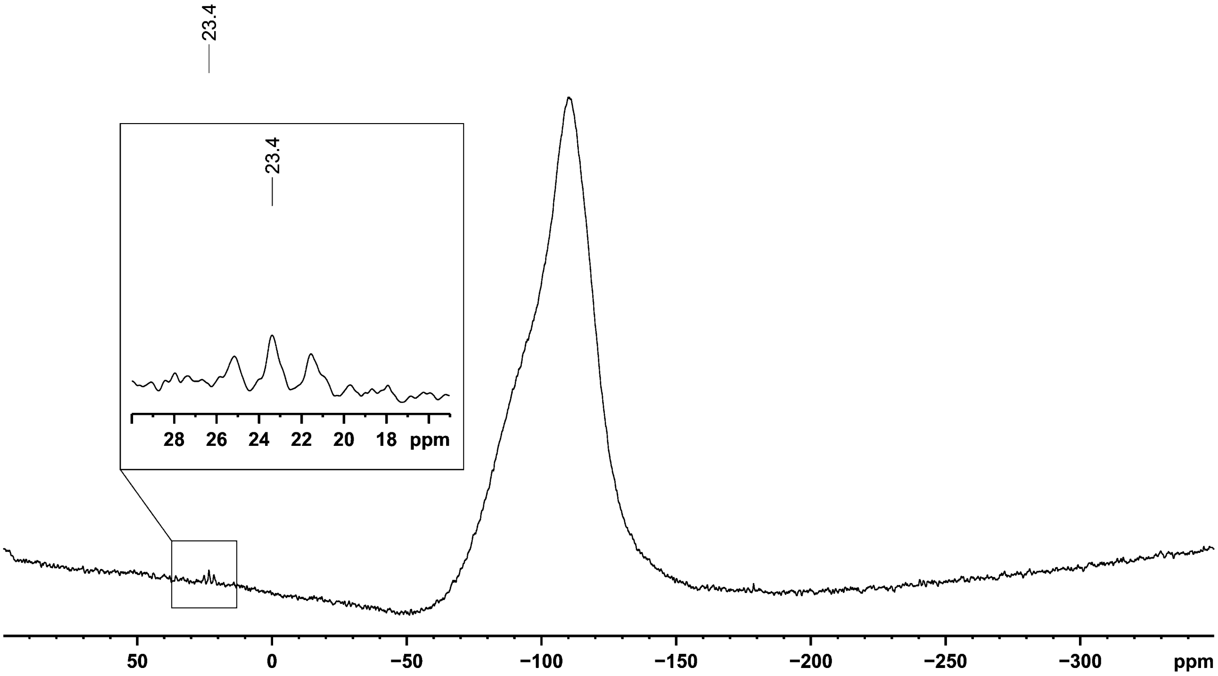

Figure S9. ^{29}Si NMR spectrum (79.49 MHz, 5120 scans, 300 K, THF-d_8) of [(η^4-P_4)Co{η^4-P_4Si(nacnac)}] (**2**).

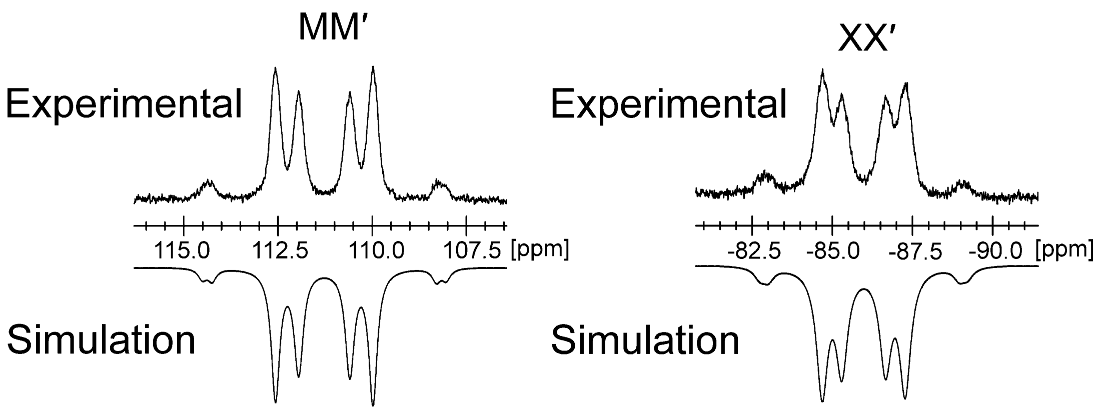

Figure S10. Section of the $^{31}P\{^1H\}$ NMR (161.98 MHz, 300 K, THF-d_8) spectrum of [(η^4-P_4)Co{η^4-P_4Si(nacnac)}] (**2**) experimental (upwards) and simulation (downwards).

Table S1. $J(^{31}P,^{31}P)$ coupling constants from the iterative fit of the $A_4MM'XX'$ spin system and schematic representation of the [(*cyclo*-P_4)CoP_4Si] core of [(η^4-P_4)Co{η^4-P_4Si(nacnac)}] (**2**); [Si] = (nacnac)Si.

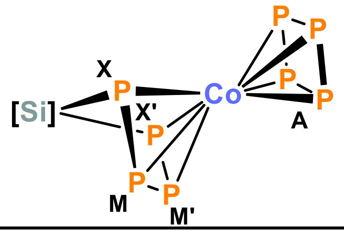

$^1J_{MM'} = {}^1J_{M'M}$	−392.6 Hz		
$^1J_{XM} = {}^1J_{X'M'}$	−444.4 Hz	δ(P_A)	175.0 ppm
$^2J_{XM'} = {}^2J_{X'M}$	24.6 Hz	δ($P_{MM'}$)	111.3 ppm
$^nJ_{XX'} = {}^nJ_{X'X}$	−24.1 Hz	δ($P_{XX'}$)	−86.0 ppm

1.4 Synthesis of [M(crypt-222)][(η^5-P_5)Co(η^3-P_3)] ([M(crypt-222)]4, M = Na, K)

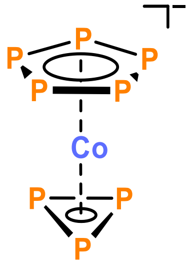

[M(crypt-222)]$^+$

KOPh (64.4 mg, 0.487 mmol, 2.0 equiv.) was dissolved in a mixture of crypt-222 (183.3 mg, 0.487 mmol, 2.0 equiv.) in THF (3 mL). The clear colorless solution was added dropwise to a brown solution of [(η^4-P_4)Co{η^4-P_4Si(nacnac)}] (**2**, 183.2 mg, 0.244 mmol, 1.0 equiv.) in THF (5 mL) while stirring. After stirring for 3 h at room temperature, a $^{31}P\{^1H\}$ NMR spectrum of the solution was recorded, revealing selective formation of **4** (Figure S11). Quantitative $^{31}P\{^1H\}$ NMR spectroscopy using Ph_3PO as an internal standard indicated that **4** was formed in 50% yield.

Synthesis of [Na(crypt-222)]4

Method A:

[Na(crypt-222)]**4** was synthesized by filtering a THF/toluene solution (1:1 v/v) of [K(crypt-222)]**4** over alumina (0.4 x 3 cm). The sodium cation originates from alumina, which contains 0.4% Na. Storage of the resulting orange solution at −35 °C gave single crystals of [Na(crypt-222)]**4** suitable for XRD analysis, which were isolated, washed with pentane and dried in *vacuo*.

Method B:

NaOPh (24.7 mg, 0.213 mmol, 2.0 equiv.) was dissolved in a mixture containing crypt-222 (80.1 mg, 0.213 mmol, 2.0 equiv.) in THF (4 mL). The resulting clear, colorless solution was added dropwise to a brown solution of [(η^4-P_4)Co{η^4-P_4Si(nacnac)}] (**2**, 80 mg, 0.106 mmol, 1.0 equiv.) in THF (4 mL) under stirring. After stirring for 4 h at room temperature, an equal volume of toluene was added, and the solution was passed through an alumina pad (1.2 × 2 cm). The alumina was subsequently washed with the same solvent mixture until the eluent became colorless. The resulting orange solution was collected, and the solvent was evaporated under reduced pressure to afford an orange, oily solid, which was washed with toluene, Et_2O, and pentane. After drying in vacuo an orange-brown solid was obtained. Yield: 30 mg (0.043 mmol, 40%).

Crystalline [Na(crypt-222)]**4** can be obtained by layering the orange solution resulting from the filtration over alumina with an equal amount of toluene and storing at −35 °C.

Data for [K(crypt-222)]4:

^{31}P{^{1}H} NMR (202.48 MHz, 300 K, THF-*d*$_8$): δ / ppm = 238.4 (s, 5P, *cyclo*-P_5), −226.0 (br s, 3P, *cyclo*-P_3).

ESI-MS (negative-ion mode, *o*-DFB): *m/z* = 306.7248 [(η^5-P_5)Co(η^3-P_3)]$^-$

ESI-MS (positive-ion mode, *o*-DFB): *m/z* = 415.2230 [K(crypt-222)]$^+$

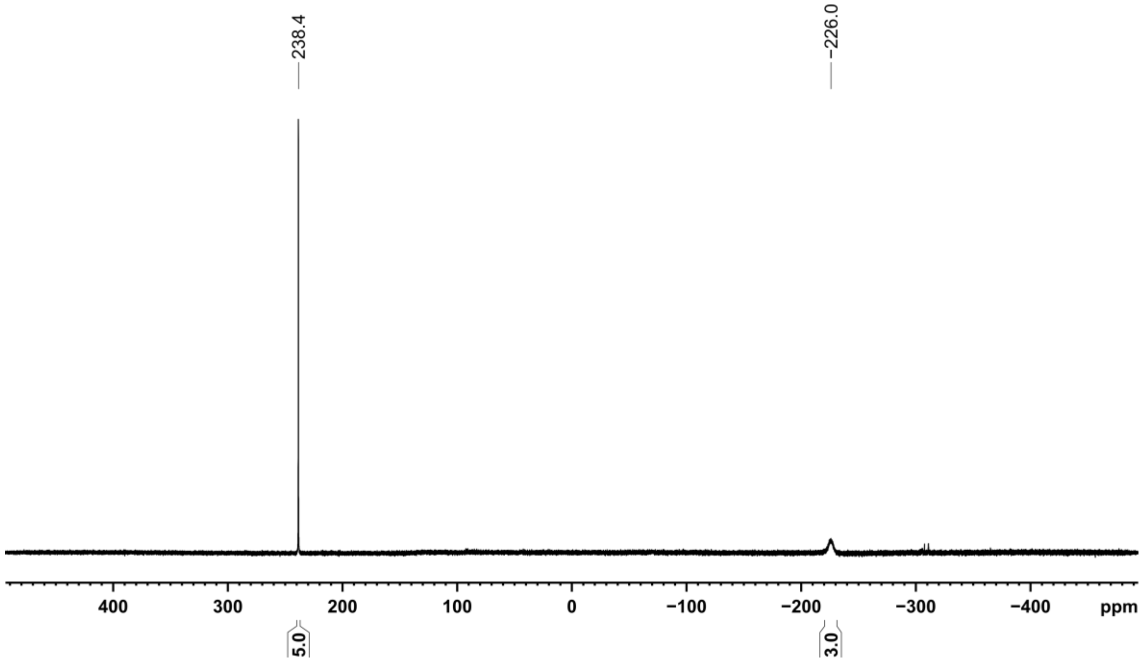

Figure S11. ^{31}P{^{1}H} NMR spectrum (202.48 MHz, 300 K, THF/C_6D_6-capillary) of the reaction solution of [K(crypt-222)][(η^5-P_5)Co(η^3-P_3)] ([K(crypt-222)]**4**).

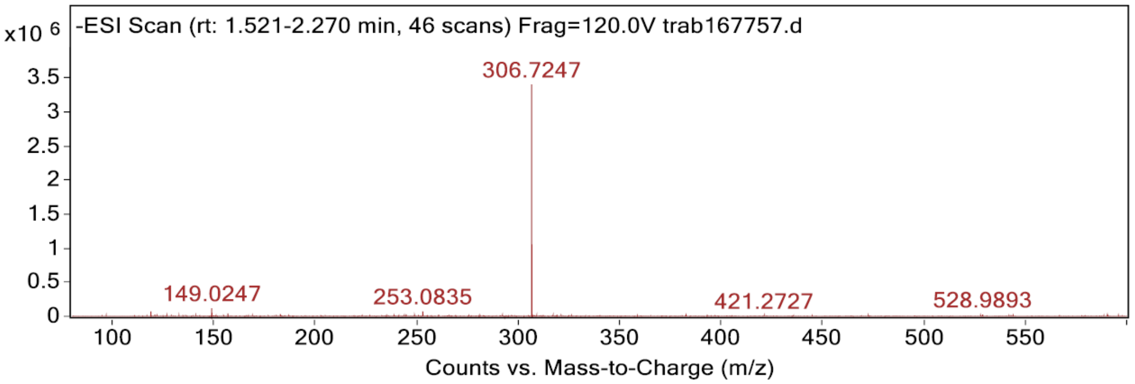

Figure S12. Negative ion ESI-MS spectrum of [K(crypt-222)][(η^5-P_5)Co(η^3-P_3)] ([K(crypt-222)]**4**) (in *o*-DFB).

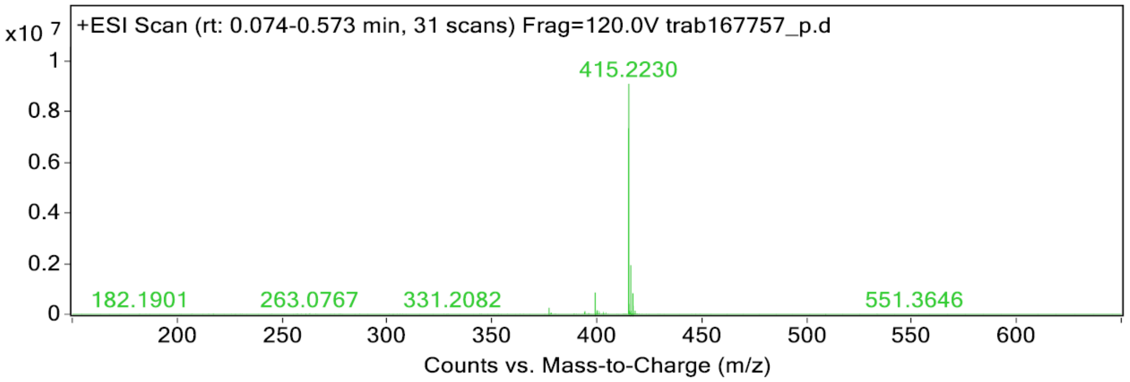

Figure S13. Positive ion ESI-MS spectrum of [K(crypt-222)][(η^5-P_5)Co(η^3-P_3)] ([K(crypt-222)]**4**) (in *o*-DFB).

Data for [Na(crypt-222)]4:

^{1}H NMR (500 MHz, 300 K, THF-d_8): δ/ppm = 2.67 (m, 12 H, *CH_2*-crypt), 3.60 (m, 12 H, *CH_2*-crypt), 3.64 (s, 12 H, *CH_2*-crypt).

^{31}P{^{1}H} NMR (202.48 MHz, 300 K, THF-d_8): δ/ppm = 237.8 (s, 5 P; *cyclo*-P_5), –226.6 (s, br., 3 P; *cyclo*-P_3).

Elemental analysis (%) calcd. for $C_{18}H_{36}CoN_2NaO_6P_8$ (M_w = 762.67 g mol^{-1}): C 30.61, H 5.14, N 3.97; found: C 32.59, H 5.31, N 4.02. The analytical value for carbon and hydrogen are higher than the calculated value for [Na(crypt-222)]$_2$**4**. This discrepancy can be attributed to unidentified impurities.

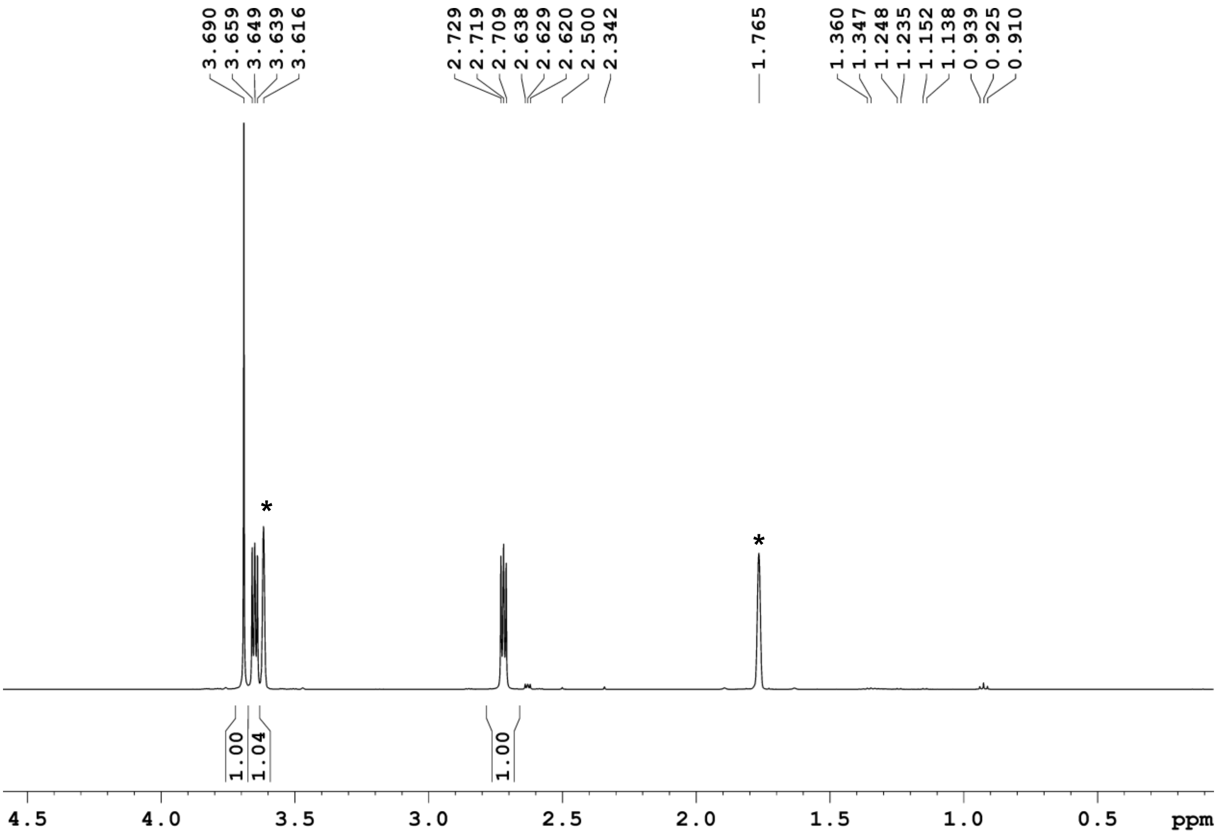

Figure S14. ^{1}H NMR spectrum (500 MHz, 300 K, THF-d_8) of [Na(crypt-222)][(η^5-P_5)Co(η^3-P_3)] ([Na(crypt-222)]**4**). * = residual deuterated solvent (THF).

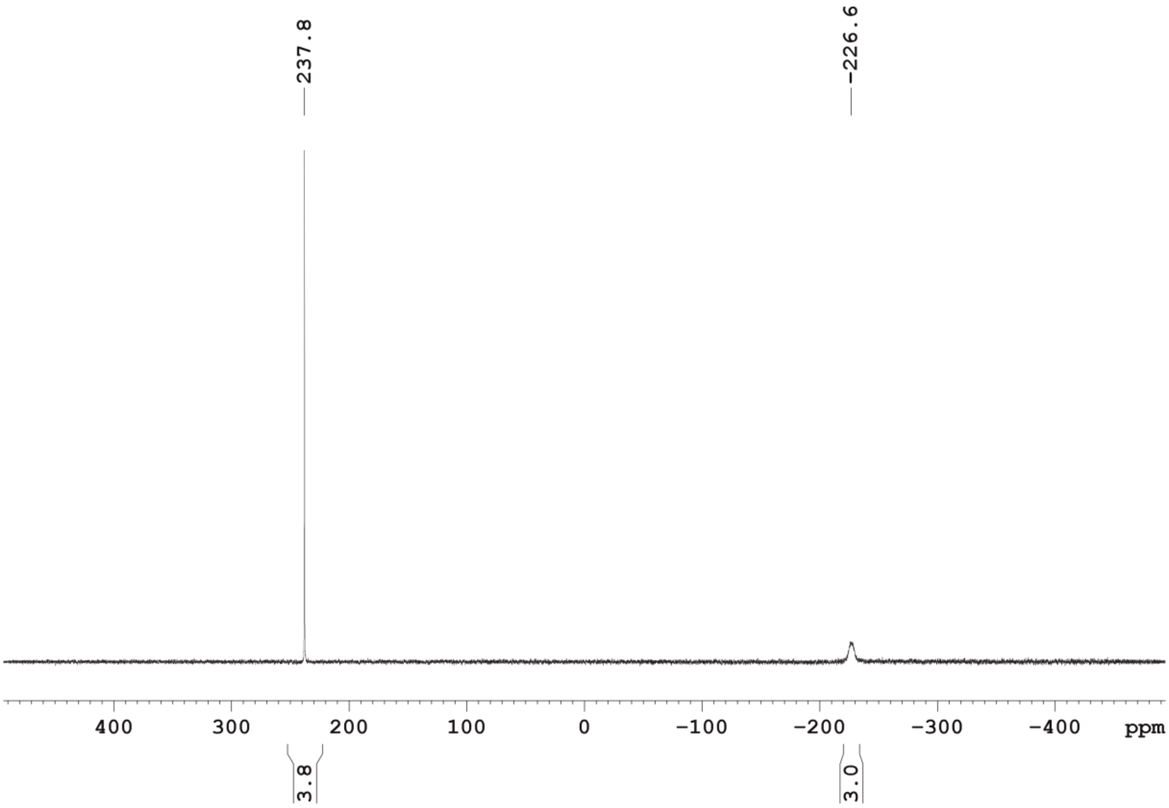

Figure S15. $^{31}P\{^{1}H\}$ NMR spectrum (202.48 MHz, 300 K, THF-d$_8$) of [Na(crypt-222)][(η^5-P_5)Co(η^3-P_3)]([Na(crypt-222)]**4**).

1.5 Synthesis of [K(crypt-222)]$_2$[(η^4-P_5)Co(η^3-P_3)] ([K(crypt-222)]$_2$5)

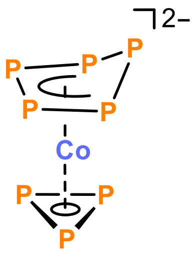

2 [K(crypt-222)]$^+$

KOPh (64.4 mg, 0.487 mmol, 2.0 equiv.) was dissolved in a mixture of crypt-222 (183.3 mg, 0.487 mmol, 2.0 equiv.) and THF (3 mL). The clear colorless solution was added dropwise to a solution of [(η^4-P_4)Co{η^4-P_4Si(nacnac)}] (**2**, 183.2 mg, 0.244 mmol, 1.0 equiv.) in THF (5 mL) while stirring. The mixture was stirred for 3 h. Subsequently, the brown reaction mixture was filtered through a Whatman glass microfiber filter. The solvent was removed in *vacuo*, and the remaining dark brown residue was washed with *n*-hexane (3 mL), Et_2O (15 x 2 mL) and toluene (10 x 2 mL). The remaining brown, oily residue was extracted with a toluene/THF mixture (1:1 *v*:*v*, 2 x 3 mL, fraction 1). Filtration through a Whatman microfiber filter yielded a brown solid on the filter, which was extracted with *o*-DFB (4 mL, fraction 2). The combined brown toluene/THF filtrates (fraction 1) were stored at room temperature for 5 d, yielding dark orange crystals of [K(crypt-222)]$_2$**5**. The mother liquor was decanted, and the crystals were sequentially washed with cold (−30 °C) Et_2O (1 mL), toluene (1 mL), and *n*-hexane (1 mL), then dried *in vacuo*. Yield (fraction 1): 23.6 mg (0.021 mmol, 9%).

The *o*-DFB solution (fraction 2) was layered with toluene (3 mL). Slow diffusion of Et_2O into this *o*-DFB/toluene solution for six days at room temperature yielded additional dark orange crystals of [K(crypt-222)]$_2$**5** suitable for XRD, which were isolated by the same procedure as fraction 1. Yield (fraction 2): 74.8 mg (0.066 mmol, 27%).

Notes: [K(crypt-222)]$_2$**5** is insoluble in nonpolar hydrocarbon solvents, but dissolves in THF, ^{Me}THF, MeCN and ethylenediamine (en) with decomposition. The instability of **5** is expected given the high reactivity of the related complex [Cp*Fe(η^4-P_5)]$^{2-}$, which is capable of deprotonating MeCN.[5] The successful crystallization of [K(crypt-222)]$_2$**5** relies on maintaining the conditions described above. If the toluene/THF (1:1 *v*:*v*) mixture is too concentrated or stored at room temperature, it will form an oil, leaving a clear supernatant.

Combined yield (two fractions): 98.4 mg (0.087 mmol, 36%)

Elemental analysis (%) calcd. for $C_{36}H_{72}CoK_2N_4O_{12}P_8$ (Mw = 1137.91 g·mol^{-1}) C 38.00, H 6.38, N 4.92, Co 5.18, P 21.78, K 6.87, O 16.87; found C 37.40, H 6.41, N 4.81, Co 5.11, P 21.62, K 6.64. The analytical value for carbon is slightly higher than the calculated value for [K(crypt-222)]$_2$**5**. This discrepancy can be attributed to impurities that we could not eliminate by recrystallization, due to the low stability in polar solvents and the insolubility in nonpolar hydrocarbon solvents.

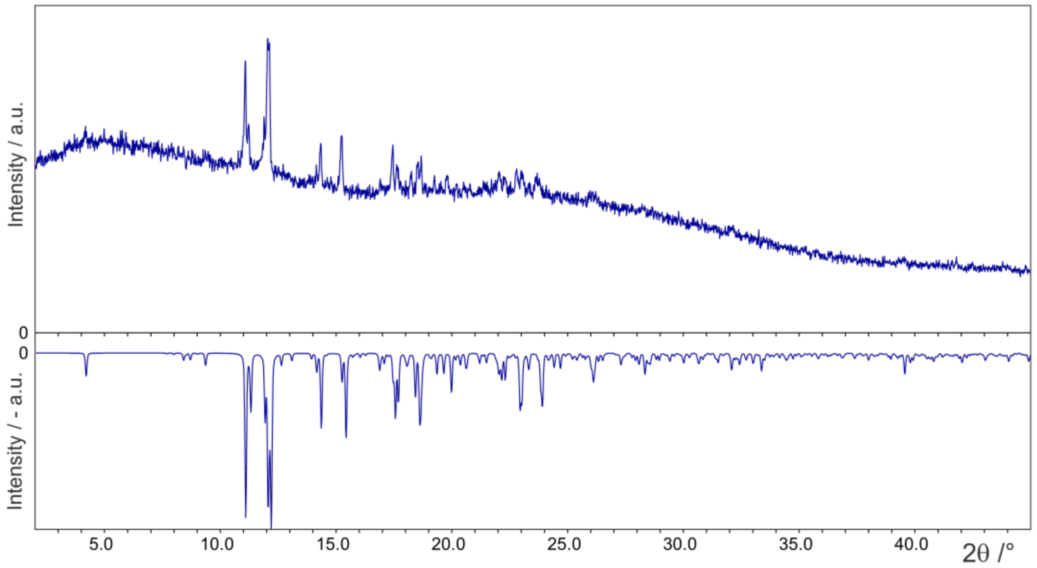

Figure S16. Experimental (upwards) and calculated (downwards) powder X-ray diffractogram of $[K(crypt\text{-}222)]_2$**5**.

2. Additional Experiments

2.1 Reaction of [K(THF)]1 with two equivalents PhOH

Solid PhOH (2.1 mg, 0.02 mmol, 2.0 equiv.) was added at room temperature to a brown solution of [K(THF)][Co{η^4-P_4Si(nacnac′)}$_2$] ([K(THF)]**1**, 14.5 mg, 0.01 mmol, 1.0 equiv.) dissolved in THF-d_8. The reaction mixture was transferred to a J. Young NMR tube. After 2.5 h, ^{1}H, ^{13}C{^{1}H}, ^{31}P{^{1}H} and 2D NMR spectra of the reaction mixture were recorded.

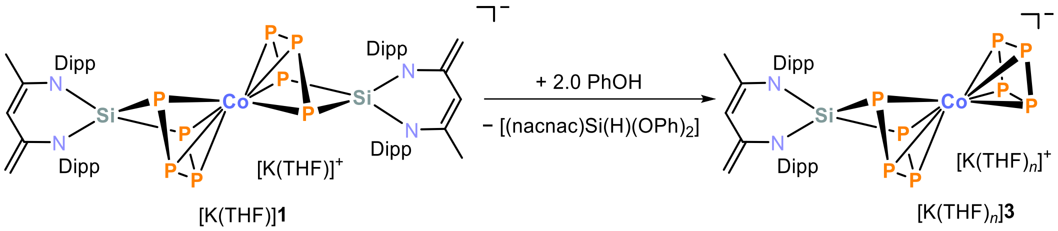

Scheme S1. Reaction of [K(THF)]**1** with two equiv. PhOH in THF-d_8 at room temperature for 2.5 h.

The proposed reaction mechanism for the desilylation of [K(THF)]**1** with two equiv. PhOH is shown in Scheme S2. The ^{31}P{^{1}H} NMR spectrum of the reaction mixture shows the selective formation of a new phosphorus-containing species arising as an $A_4MM'XX'$ spin system with an integral ratio of 4:2:2 at δ = 159.3 (P_A), 108.0 ($P_{MM'}$) and –63.7 ($P_{XX'}$) ppm (Figure S18). This spin system is very similar to the spin system observed for compound **4** (*vide supra*), which allows for the assignment of this spin system to [K(THF)$_n$][(η^4-P_4)Co{(η^4-P_4Si(nacnac′)}] ([K(THF)$_n$]**3**). Due to the lower acidity of PhOH (pK_a ≈ 10) in comparison to benzoic acid (pK_a ≈ 4), protonation occurs only at one nacnac′ ligand of [K(THF)]**1**. The phenolate anion (PhO^-) released in this process attacks at the Si atom, forming the silicon(II) compound [(nacnac)Si(OPh)]. A subsequent oxidative addition of PhOH to the Si atom releases the silicon(IV) species [(nacnac)Si(H)(OPh)$_2$].

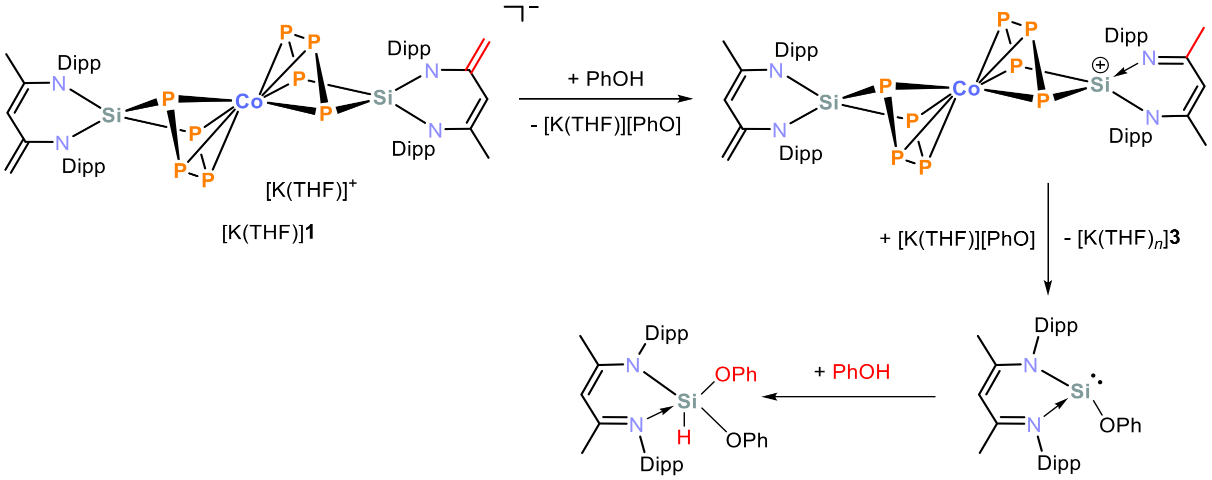

Scheme S2. Proposed reaction mechanism for the reaction of [K(THF)]**1** with two equiv. PhOH in THF-d_8 at room temperature.

The presence of the nacnac′ ligand in **3** in its deprotonated, dianionic form is clearly revealed by the ^{1}H NMR spectrum of the reaction mixture, which shows two stereoisomers in a 1:1.2 integral ratio (Figure S17), as confirmed by ^{1}H,^{1}H-COSY and ^{1}H,^{1}H-NOESY NMR experiments. The product labeled with ■ is assigned to **3**, based on the singlet resonances at δ = 1.70 ppm (3H), 2.56 ppm (1H), 3.38 ppm (1H) and 5.07 ppm (1H) for the exocyclic CH_3,

CH_2 and CH groups of the β-diketiminate backbone. The second product labeled with ● is assigned to [(nacnac)Si(H)(OPh)$_2$]. This is evidenced by the singlet resonances at δ = 1.86 ppm assigned to the NCCH_3 groups (six hydrogen atoms) and δ = 5.61 ppm assigned to the NCH group of the β-diketiminate backbone. No signal corresponding to a methylene group (NCCH_2) was observed. A singlet resonating at δ = 5.02 ppm is assigned to the a Si-H unit. Roesky and co-workers reported the formation of the related compound [(nacnac)Si(H)(OC_6F_5)$_2$] showing similar ^{1}H NMR resonances through the reaction of [(nacnac′)Si(η^2-P_4)] with two equivalents C_6F_5OH.[6] The proposed [(nacnac)Si(H)(OPh)$_2$] also forms in the reaction of [(nacnac′)Si] with two equivalents PhOH (*vide infra*).

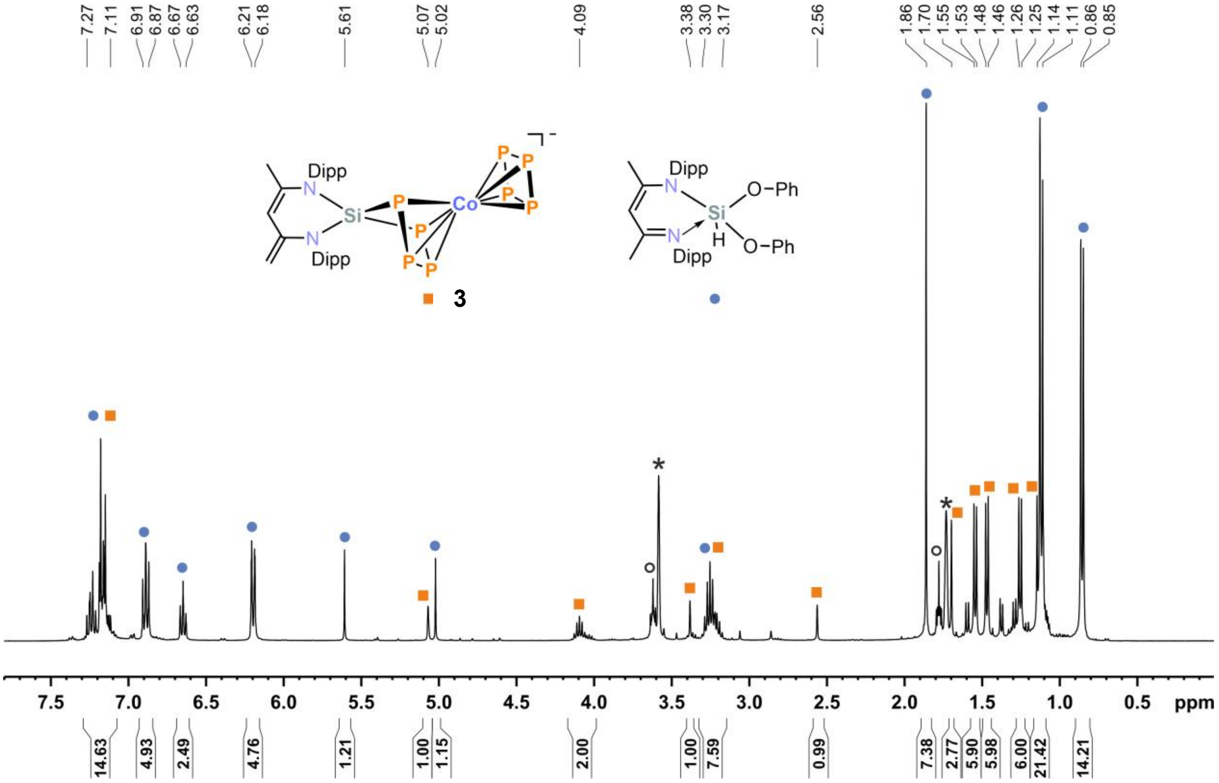

Figure S17. ^{1}H NMR spectrum (400.30 MHz, 300 K, THF-d_8) of the reaction of [K(THF)][Co{η^4-P_4Si(nacnac′)}$_2$] ([K(THF)]**1**) with two equivalents PhOH. Compound ■: [(η^4-P_4)Co{η^4-P_4Si(nacnac′)}] (**2**). Compound ●: [(nacnac)Si(H)(OPh)$_2$]. * = partially deuterated THF, ○ = THF.

^{1}H NMR (400.30 MHz, 300 K, THF-d_8): δ / ppm = 0.86 (d, $^3J_{HH}$ = 6.7 Hz, 12H, Dipp: CH*Me₂* of ●), 1.11-1.14 (m, 18H, Dipp: CHMe_2 of ● overlapping with Dipp: CHMe_2 of ■), 1.26 (d, $^3J_{HH}$ = 6.7 Hz, 6H, Dipp: CHMe_2 of ■), 1.48 (d, $^3J_{HH}$ = 6.7 Hz, 6H, Dipp: CHMe_2 of ■), 1.54 (d, $^3J_{HH}$ = 6.7 Hz, 6H, Dipp: CHMe_2 of ■), 1.70 (s, 3H, ^{Dipp}nacnac′: NCCH_3 of ■), 1.86 (s, 6H, ^{Dipp}nacnac: NCCH_3 of ●), 2.56 (s, 1H, ^{Dipp}nacnac′: NCCH_2 of ■), 3.17-3.30 (m, 6H, Dipp: C*H*Me$_2$ of ■ overlapping with Dipp: C*H*Me$_2$ of ●), 3.38 (s, 1H, ^{Dipp}nacnac′: NCCH_2 of ■), 4.09 (sept, $^3J_{HH}$ = 6.7 Hz, 2H, Dipp: C*H*Me$_2$ of ■), 5.02 (s, 1H, Si*H* of ●), 5,07 (s, 1H, γ-C*H* of ■), 5.61 (s, 1H, γ-C*H* of ●), 6.18-6.21 (m, 4H, –OC_6H_5 of ●), 6.63-6.67 (m, 2H, –OC_6H_5 of ●), 6.87-6.91 (m, 4H, –OC_6H_5 of ●), 7.11-7.27 (m, 12H, Dipp: 2,6-*i*Pr$_2C_6H_3$).

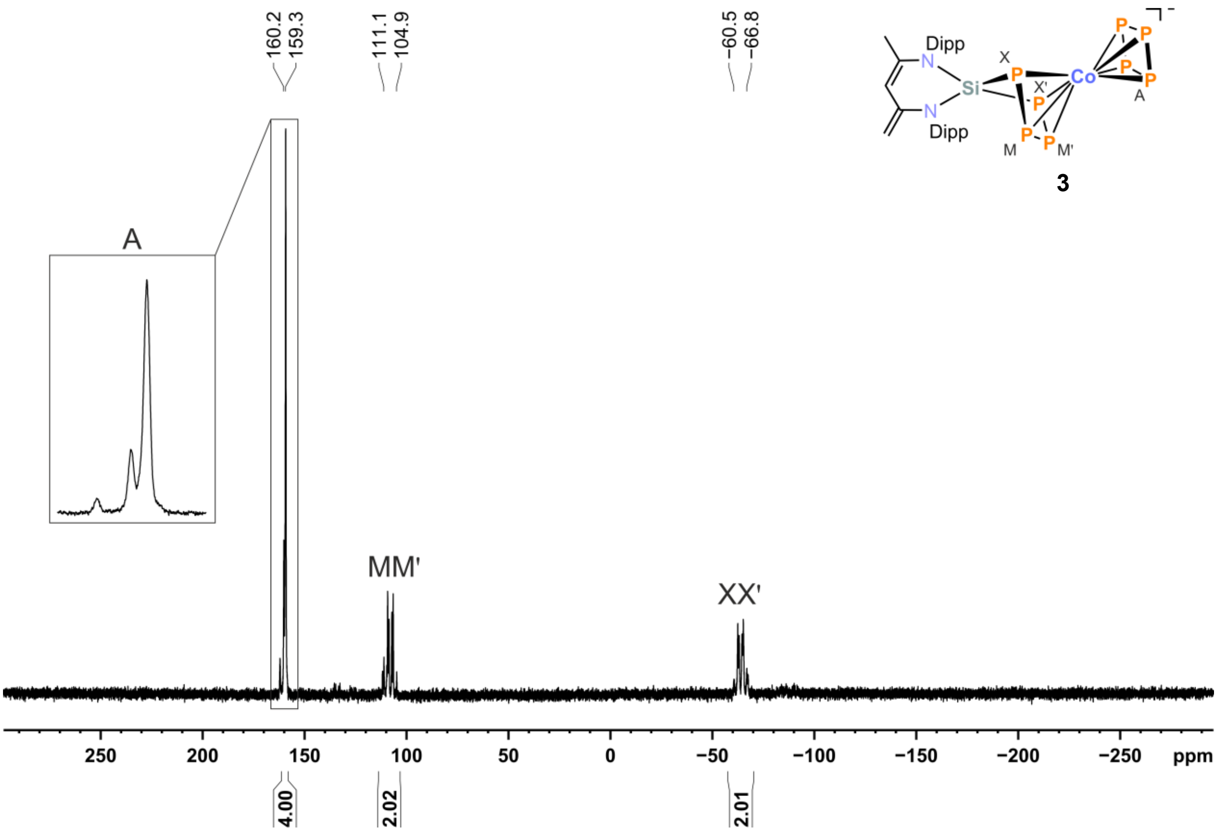

Figure S18. $^{31}P\{^{1}H\}$ NMR spectrum (162.04 MHz, 300 K, THF-d_8) of the reaction of [K(THF)][Co{(η^4-P_4)Si(nacnac′)}$_2$] ([K(THF)]**1**) with two equivalents PhOH. Inset: extended signal arising from the *cyclo*-P_4 unit of **3** indicating the presence of two isomers in solution based on the relative orientation of the C=C double bond of the nacnac′ β-diketiminate ligand.

$^{31}P\{^{1}H\}$ NMR (162.04 MHz, 300 K, THF-d_8): δ / ppm = 159.3 (m, 4 P, P_A), 108.0 (m, 2 P, $P_{MM'}$), –63.7 (m, 2 P, $P_{XX'}$).

2.2 Reaction of [(nacnac′)Si] with PhOH (2.0 equiv.)

A J. Young NMR tube was charged with [(nacnac′)Si] (27.2 mg, 0.06 mmol, 1.0 equiv.) and PhOH (11.6 mg, 0.12 mmol, 2.0 equiv.) and dissolved in THF-d_8 (0.5 mL) was added. After 3 h, the ^{1}H NMR spectrum showed a mixture of two products in a 1:1 integral ratio, which were identified *via* ^{1}H-^{1}H COSY and NOESY NMR spectroscopy as [(nacnac)Si(H)(OPh)$_2$] (labeled ●) and the free (nacnac)H ligand (labeled ●).

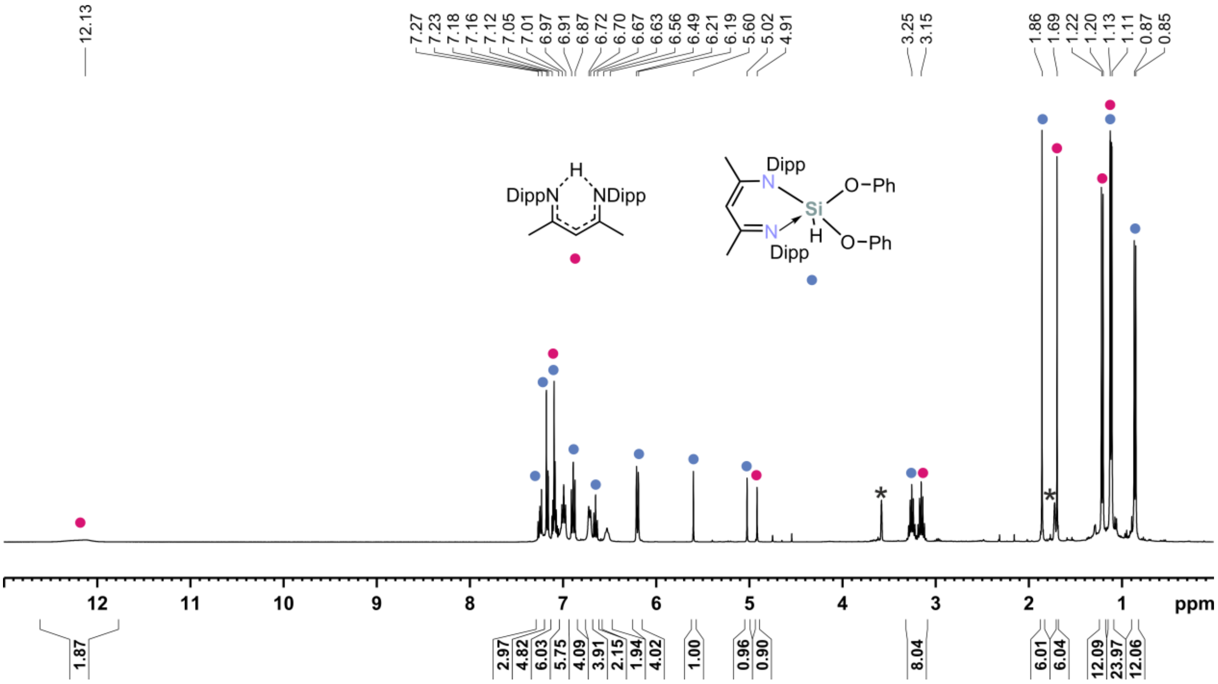

Figure S19. ^{1}H NMR spectrum (400.30 MHz, 300 K, THF-d_8) of the reaction of [(nacnac′)Si] with two equivalents PhOH. Compound ●: [(nacnac)Si(H)(OPh)$_2$]. Compound ●: (nacnac)H. * partially deuterated THF.

^{1}H NMR (400.30 MHz, 300 K, THF-d_8): δ / ppm = 0.86 (d, $^3J_{HH}$ = 6.7 Hz, 12H, Dipp: CH*Me₂* of ●), 1.11-1.13 (m, 24H, Dipp: CH*Me₂* of ● overlapping with Dipp: CH*Me₂* of ●), 1.21 (d, $^3J_{HH}$ = 7.0 Hz, 12H, Dipp: CH*Me₂* of ●), 1.69 (s, 6H, ^{Dipp}nacnac: NCC*H₃* of ●), 1.86 (s, 6H, ^{Dipp}nacnac: NCC*H₃* of ●), 3.15-3.25 (m, 8H, Dipp: C*H*Me₂ of ● overlapping with Dipp: C*H*Me₂ of ●), 4.91 (s, 1H, , γ-C*H* of ●), 5.02 (s, 1H, Si*H* of ●), 5.60 (s, 1H, γ-C*H* of ●), 6.19-6.21 (m, 4H, −OC$_6$*H₅* of ●), 6.49-6.56 (m, 2H, −OC$_6$*H₅*), 6.63-6.67 (m, 2H, −OC$_6$*H₅* of ●), 6.70-6.72 (m, 4H, −OC$_6$*H₅*), 6.87-6.91 (m, 4H, −OC$_6$*H₅* of ●), 6.97-7.01 (m, 6H, −OC$_6$*H₅*), 7.05-7.12 (m, 6H, Dipp: 2,6-*i*Pr$_2$C$_6$*H₃* of ● overlapping with Dipp: 2,6-*i*Pr$_2$C$_6$*H₃* of ●), 7.16-7.18 27 (m, 5H, Dipp: 2,6-*i*Pr$_2$C$_6$*H₃* of ●), 7.23-7.27 (m, 3H, Dipp: 2,6-*i*Pr$_2$C$_6$*H₃* of ●), 12.13 (br m, 2H, nacnac*H* of ●).

2.3 Reaction of [K(THF)]1 with PhCOOH (2.0 equiv.)

[K(THF)][Co{η^4-P_4Si(nacnac′)}$_2$] ([K(THF)]**1**, 13.8 mg, 0.01 mmol, 1.0 equiv.) was dissolved in THF-d_8 (0.4 mL) in a J. Young NMR tube. Solid PhCOOH (2.5 mg, 0.02 mmol, 2.0 equiv.) was added to the brown solution of at room temperature. After 5 h, ^{1}H, ^{31}P{^{1}H} and 2D NMR spectra of the reaction mixture were recorded.

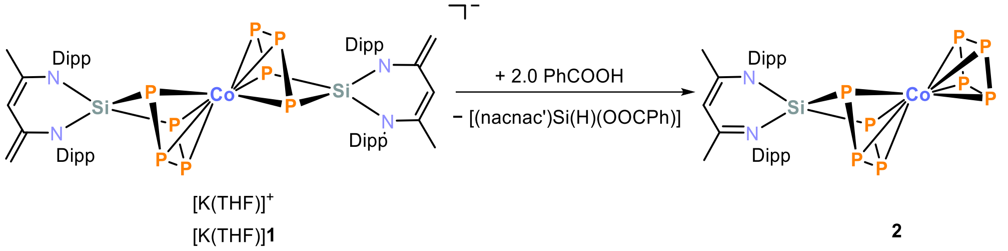

2

Scheme S3. Reaction of [K(THF)]**1** with two equiv. PhCOOH in THF-d_8 at room temperature for 5 h.

The ^{31}P{^{1}H} NMR spectrum of the reaction mixture shows the selective formation of compound **2**, which is identified by an $A_4MM'XX'$ spin system with an integral ratio of 4:2:2 at δ = 174.1 (P_A), 110.3 ($P_{MM'}$) and –87.0 ($P_{XX'}$) ppm (Figure S21).[7] The ^{1}H NMR spectrum of the reaction mixture (Figure S20) shows the formation of two main products, as confirmed by ^{1}H,^{1}H-COSY and ^{1}H,^{1}H-NOESY NMR experiments. One is identified as compound **2** (labeled with ■), whereas the second compound is assigned to [(nacnac′)Si(H)(OOCPh)] (labeled with ●). The nacnac′ ligand in [(nacnac′)Si(H)(OOCPh)] is clearly identified by the singlet resonances at δ = 1.62 ppm (3H), 3.08 ppm (1H), 3.78 ppm (1H) and 5.41 ppm (1H) for the exocyclic CH_3, CH_2 and CH groups of the β-diketiminate backbone. A singlet resonance at δ = 5.09 ppm is assigned to the Si-H unit. [(nacnac′)Si(H)(OOCPh)] is also observed in the reaction of [(nacnac′)Si] with one equivalent PhCOOH (*vide infra*). Additional resonances in the aromatic region of the ^{1}H NMR spectrum (δ = 7.12-7.69 ppm) are presumably assigned to the by-product KOOCPh.

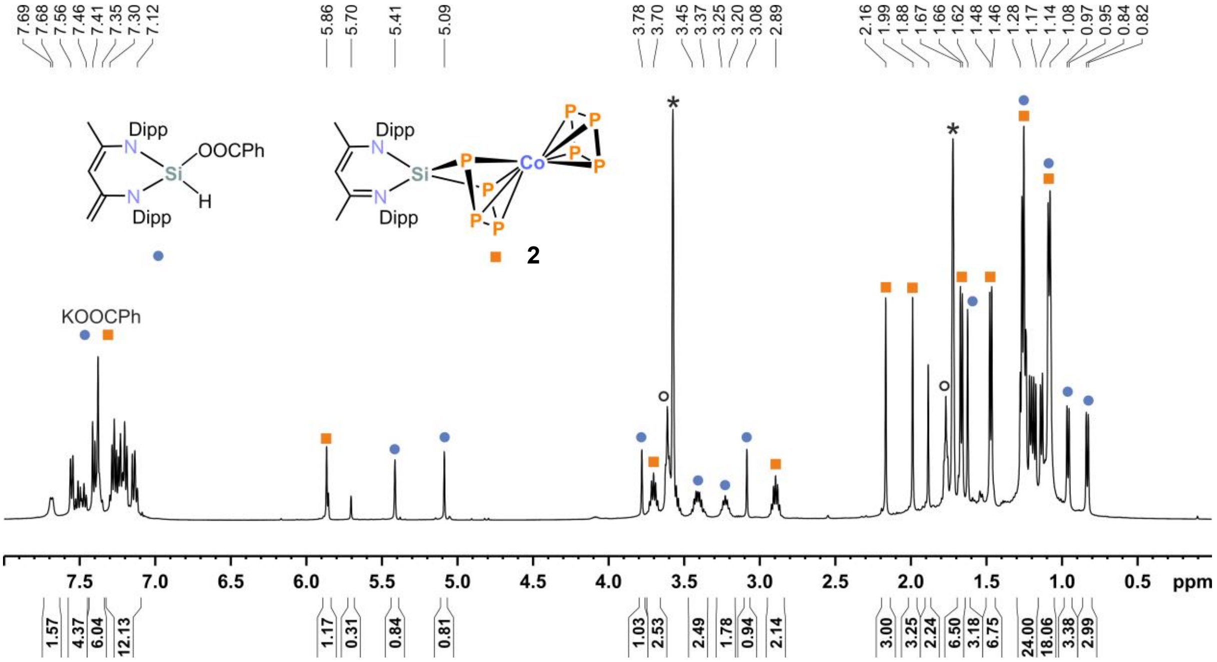

Figure S20. ^{1}H NMR spectrum (500.18 MHz, 300 K, THF-d_8) of the reaction of [K(THF)][Co{η^4-P_4Si(nacnac′)}$_2$] ([K(THF)]**1**) with two equivalents PhCOOH. Compound ■: [(η^4-P_4)Co{η^4-P_4Si(nacnac)}] (**2**). Compound ●: [(nacnac′)Si(H)(OOCPh)]. * = partially deuterated THF, ○ = THF.

^{1}H NMR (500.18 MHz, 300 K, THF-d_8): δ / ppm = 0.83 (d, $^{3}J_{HH}$ = 6.8 Hz, 3H, Dipp: CH*Me*$_2$ of ●), 0.96 (d, $^{3}J_{HH}$ = 6.8 Hz, 3H, Dipp: CH*Me*$_2$ of ●), 1.08-1.14 (m, 18H, Dipp: of ● overlapping with Dipp: CH*Me*$_2$ of ■), 1.17-1.28 (m, 24H, Dipp: CH*Me*$_2$ of ● overlapping with Dipp: CH*Me*$_2$ of ■), 1.47 (d, $^{3}J_{HH}$ = 6.6 Hz, 6H, Dipp: CH*Me*$_2$ of ■), 1.62 (s, 3H, ^{Dipp}nacnac′: NCC*H*$_3$ of ●), 1.67 (d, $^{3}J_{HH}$ = 6.6 Hz, 6H, Dipp: CH*Me*$_2$ of ■), 1.99 (s, 3H, ^{Dipp}nacnac: NCC*H*$_3$ of ■), 2.16 (s, 3H, ^{Dipp}nacnac: NCC*H*$_3$ of ■), 2.89 (sept, 2H, Dipp: C*H*Me$_2$ of ■), 3.08 (s, 1H, ^{Dipp}nacnac′: NCC*H*$_2$ of ●), 3.20-3.25 (m, 2H, Dipp: C*H*Me$_2$ of ●), 3.37-3.45 (m, 2H, Dipp: C*H*Me$_2$ of ●), 3.70 (sept, 2H, Dipp: C*H*Me$_2$ of ■), 3.78 (s, 1H, ^{Dipp}nacnac′: NCC*H*$_2$ of ●), 5.09 (s, 1H, Si*H* of ●), 5.41 (s, 1H, γ-C*H* of ●), 5.86 (s, 1H, γ-C*H* of ■), 7.12-7.30 (m, 12H, Dipp: 2,6-*i*Pr$_2$C$_6$*H*$_3$ or –OOC$_6$*H*$_5$), 7.35-7.41 (m, 6H, Dipp: 2,6-*i*Pr$_2$C$_6$*H*$_3$ or –OOC$_6$*H*$_5$), 7.46-7.56 (m, 4H, Dipp: 2,6-*i*Pr$_2$C$_6$*H*$_3$ or –OOC$_6$*H*$_5$), 7.68-7.69(m, 2H, Dipp: 2,6-*i*Pr$_2$C$_6$*H*$_3$ or –OOC$_6$*H*$_5$).

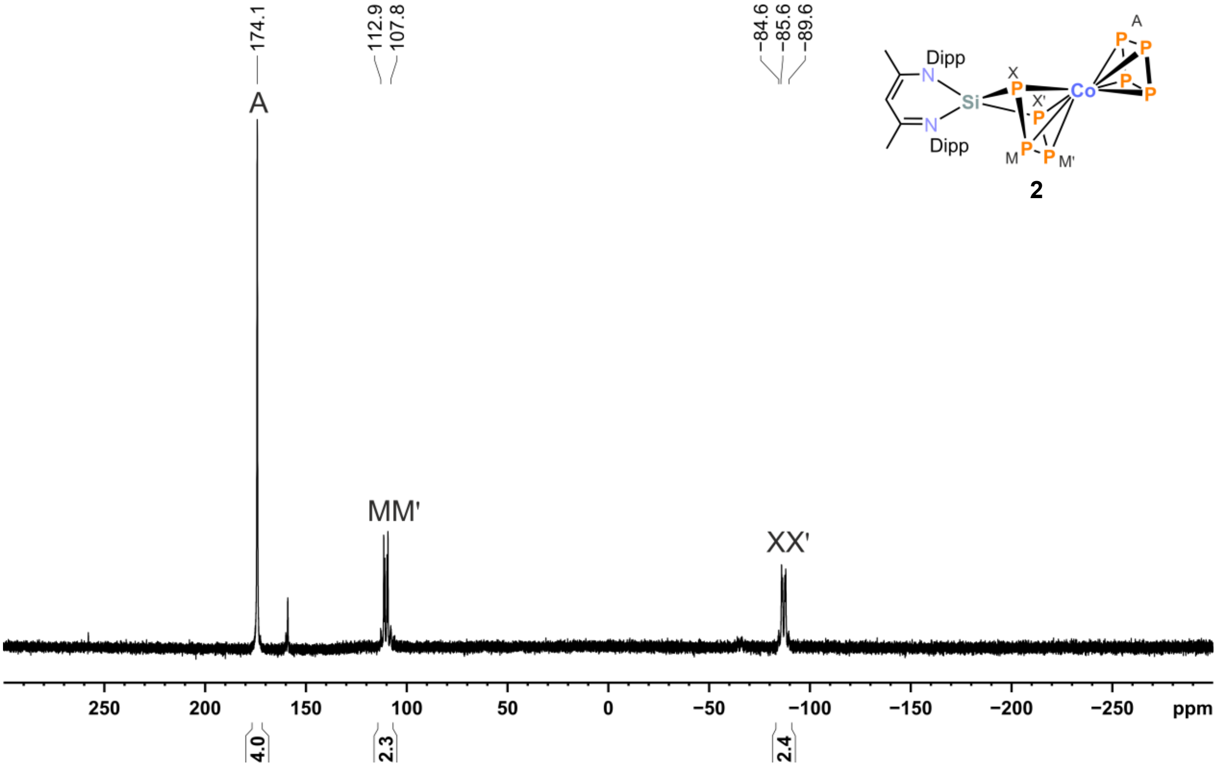

Figure S21. ^{31}P{^{1}H} NMR spectrum (202.48 MHz, 300 K, THF-d_8) of the reaction of [K(THF)][Co{η^{4}-P$_4$Si(nacnac′)}$_2$] ([K(THF)]**1**) with two equivalents PhCOOH.

^{31}P{^{1}H} NMR (202.48 MHz, 300 K, THF-d_8): δ / ppm = 174.1 (s, 4 P, P$_A$), 110.3 (m, 2 P, P$_{MM'}$), –87.0 (m, 2 P, P$_{XX'}$).

2.4 Reaction of [(nacnac′)Si] with PhCOOH

Solid [(nacnac′)Si] (18.3 mg, 0.04 mmol, 1.0 equiv.) and PhCOOH (5.0 mg, 0.04 mmol, 1.0 equiv.) were submitted to a J. Young NMR tube and dissolved in THF-d_8 (0.4 mL). After 5 h, ^{1}H, ^{1}H-^{1}H COSY and ^{1}H-^{1}H NOESY NMR spectra were recorded. Three products can be observed in the ^{1}H NMR spectrum of the reaction mixture (Figure S22a), which can be assigned to [(nacnac′)Si(H)(OOCPh)] (labeled with ●), (nacnac)H ligand (labeled with ●)and [(nacnac)Si(H)(OOCPh)$_2$], as indicated by the characteristic singlet resonances at δ = 1.89 ppm (NCCH_3 groups for six hydrogen atoms) and δ = 5.71 ppm (NCH group) of the β-diketiminate backbone and the SiH resonance at δ = 5.85 ppm. After the addition of a second equivalent PhCOOH, the signals assigned to [(nacnac′)Si(H)(OOCPh)] disappear, while the signals assigned to (nacnac)H and [(nacnac)Si(H)(OOCPh)$_2$] remain (Figure S22b).

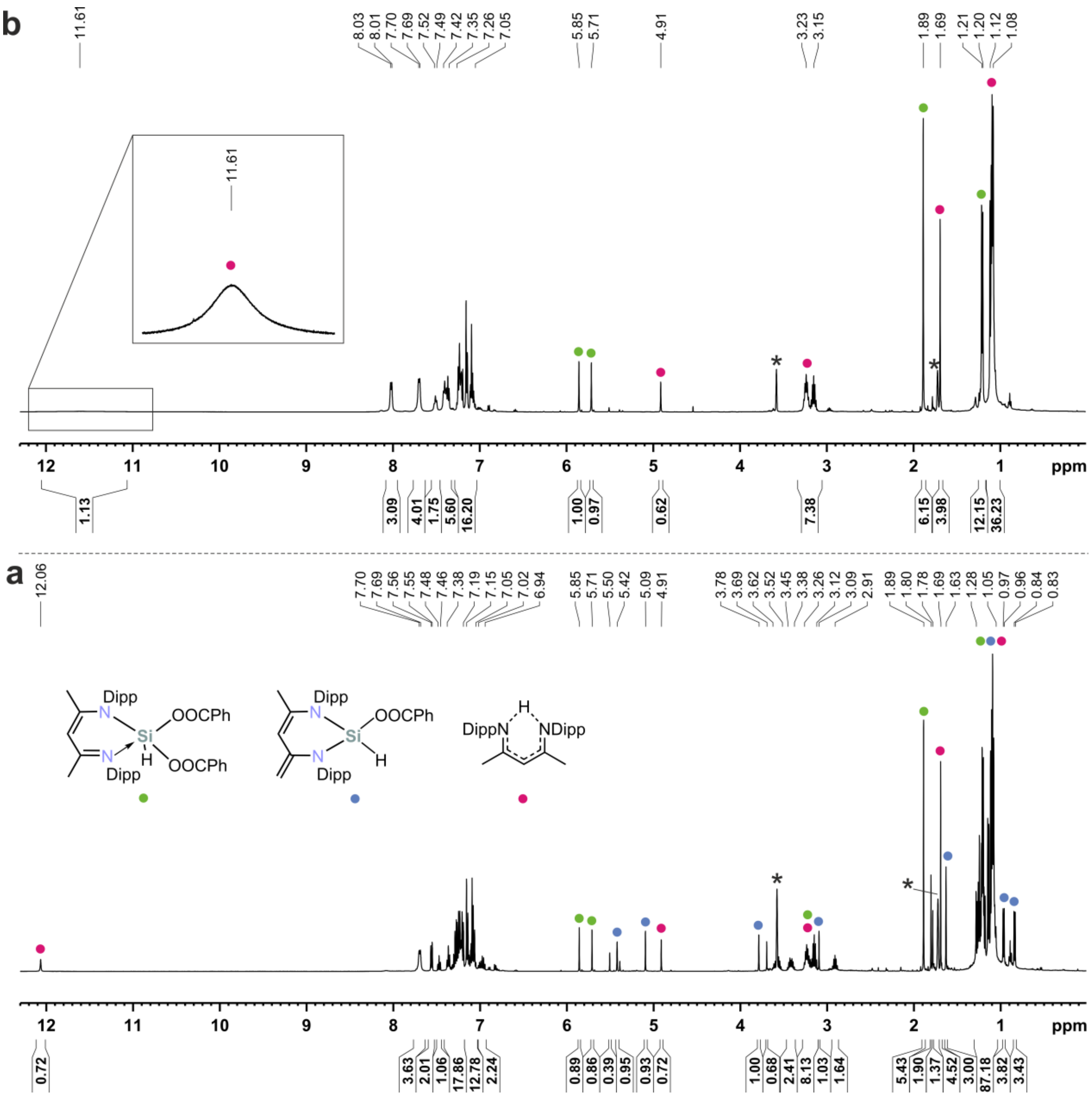

Figure S22. ^{1}H NMR spectrum (500.18 MHz, 300 K, THF-d_8) of the reaction of [(nacnac′)Si] with (a) one equiv. PhCOOH and (b) two equiv. PhCOOH. Compound ●: [(nacnac′)Si(H)(OOCPh)]. Compound ●: [(nacnac)Si(H)(OOCPh)$_2$]. Compound ●: (nacnac)H * = partially deuterated THF.

2.5 Reaction of [(η⁴-P_4)Co{η⁴-P_4Si(nacnac)}] (2) with KOPh (1.0 equiv.) and crypt-222 (1.0 equiv.)

KOPh (5.9 mg, 0.045 mmol, 1.0 equiv.) was dissolved in THF-d_8 (1 mL) and added to solid crypt-222 (16.8 mg, 0.045 mmol, 1.0 equiv.). The resulting clear colorless solution was added dropwise to [(η^4-P_4)Co{η^4-P_4Si(nacnac)}] (**2**, 33.6 mg, 0.045 mmol, 1.0 equiv.) suspended in THF-d_8 (1 mL) at room temperature. The brown reaction mixture was stirred at room temperature. ^{31}P{^{1}H} NMR spectrum of the reaction mixture was recorded after 2 d and 3 d, respectively (Figure S23).

The 1:1 reaction of **2** and KOPh does not fully consume the starting material. After 2 d reaction time, the A_4MM'XX' spin system assigned to **3** (*vide supra*, labeled with ■) and the singlets assigned to **4** (*vide supra*, labeled with ●) are present in a 1:1 integral ratio. After 3 d reaction time (Figure S23), the signal intensities of [K(THF)$_n$]**3** decrease relative to **1** and signals associated with an unidentified side product appear (labeled with ○).

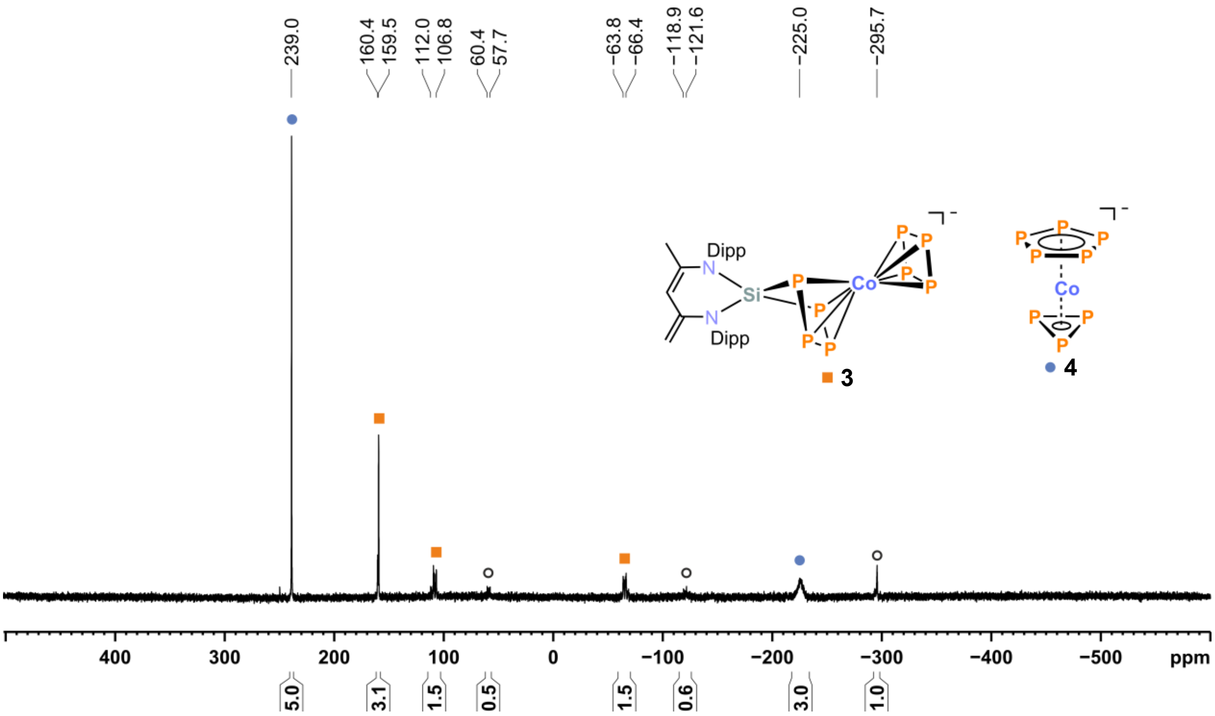

Figure S23. ^{31}P{^{1}H} NMR spectrum (162.04 MHz, 300 K, THF-d_8) of the reaction mixture of [K(THF)]**1** with 1.0 equiv. KOPh and 1.0 equiv. crypt-222 after 3 d. ○ = unidentified side product.

2.6 Reaction of [(η[4]-P_4)Co{η[4]-P_4Si(nacnac)}] (2) with KOPh (2.0 equiv.) and crypt-222 (2.0 equiv.)

Crypt-222 (14.5 mg, 0.039 mmol, 2.0 equiv.) was dissolved in THF-d_8 (0.8 mL) and added to solid KOPh (5.1 mg, 0.039 mmol, 2.0 equiv.). A J. Young valve NMR tube was charged with [(η^4-P_4)Co{η^4-P_4Si(nacnac)}] (**2**, 14.5 mg, 0.019 mmol, 1.0 equiv.), and the clear colorless solution of KOPh and crypt-222 was added dropwise at room temperature. After a reaction time of 12 h, $^{31}P\{^1H\}$ NMR spectra were recorded in a temperature range from 300 K to 193 K in 20 K steps.

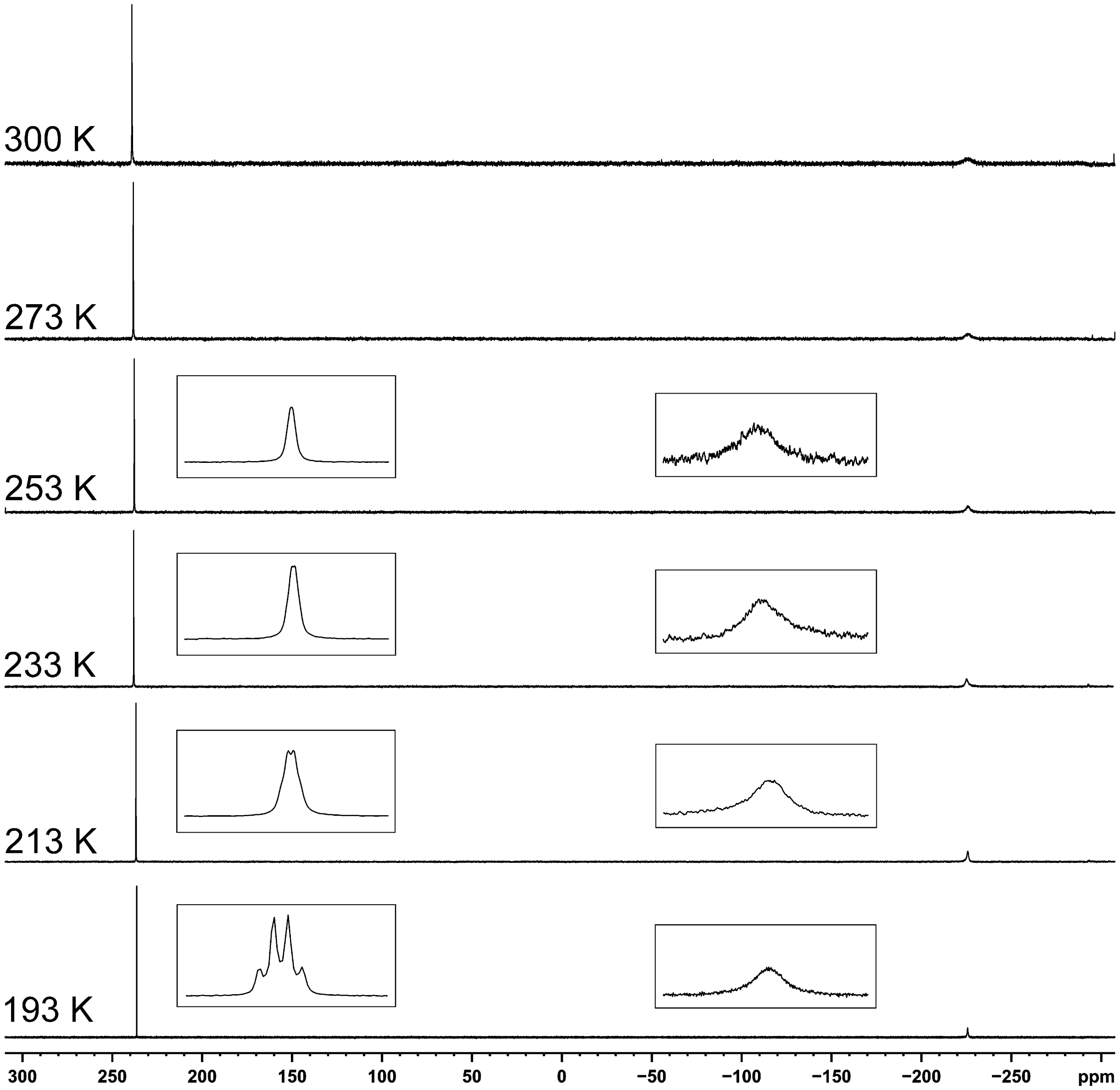

Figure S24. $^{31}P\{^1H\}$ NMR spectra (161.98 MHz, THF-d_8) of the reaction mixture of **2** with 2.0 equiv. KOPh and 2.0 equiv. crypt-222 recorded at variable temperature. Inlets: Zoomed in signals.

The stability of **4** in the reaction solution was monitored for 9 d. The decrease of the signal/noise ratio was accompanied by the formation of a brown precipitate that did not give any signals in the $^{31}P\{^{1}H\}$ NMR spectrum when dissolved in DMF-d_7.

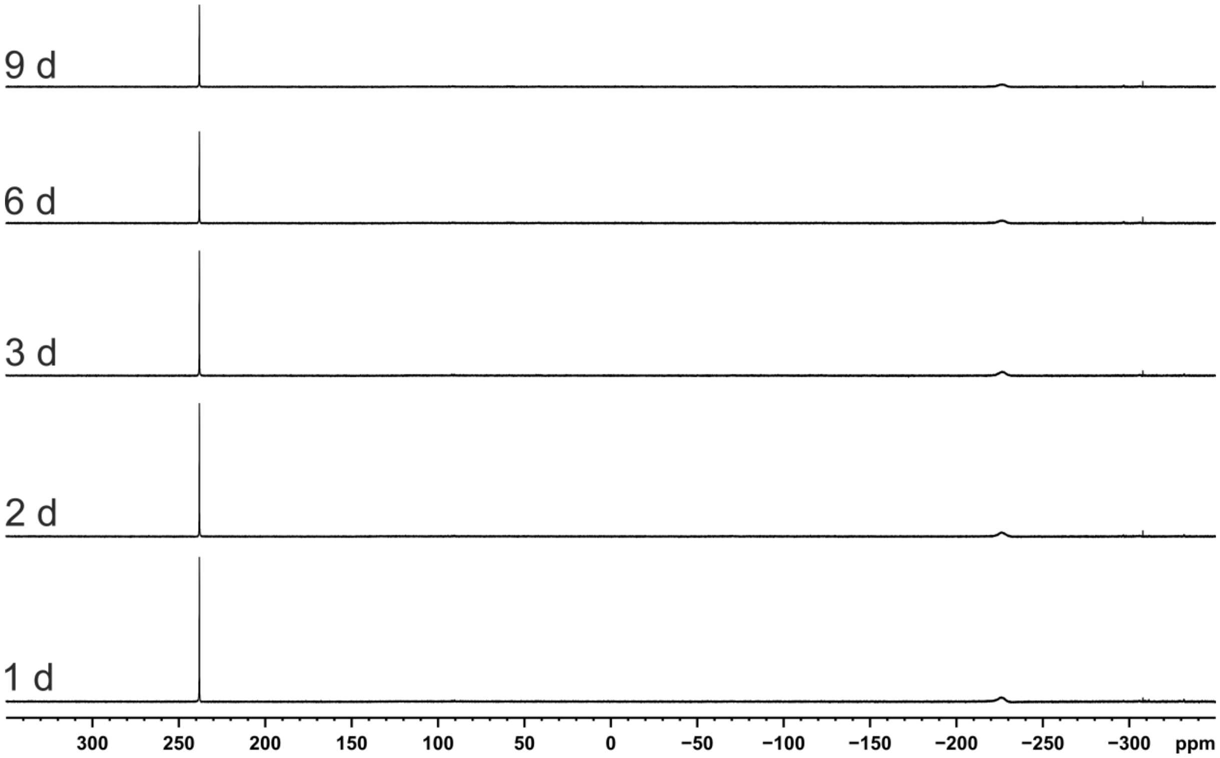

Figure S25. $^{31}P\{^{1}H\}$ NMR spectra (202.48 MHz, THF-d_8) of the reaction mixture of **2** with 2.0 equiv. KOPh and 2.0 equiv. crypt-222 recorded over the course of 9 d.

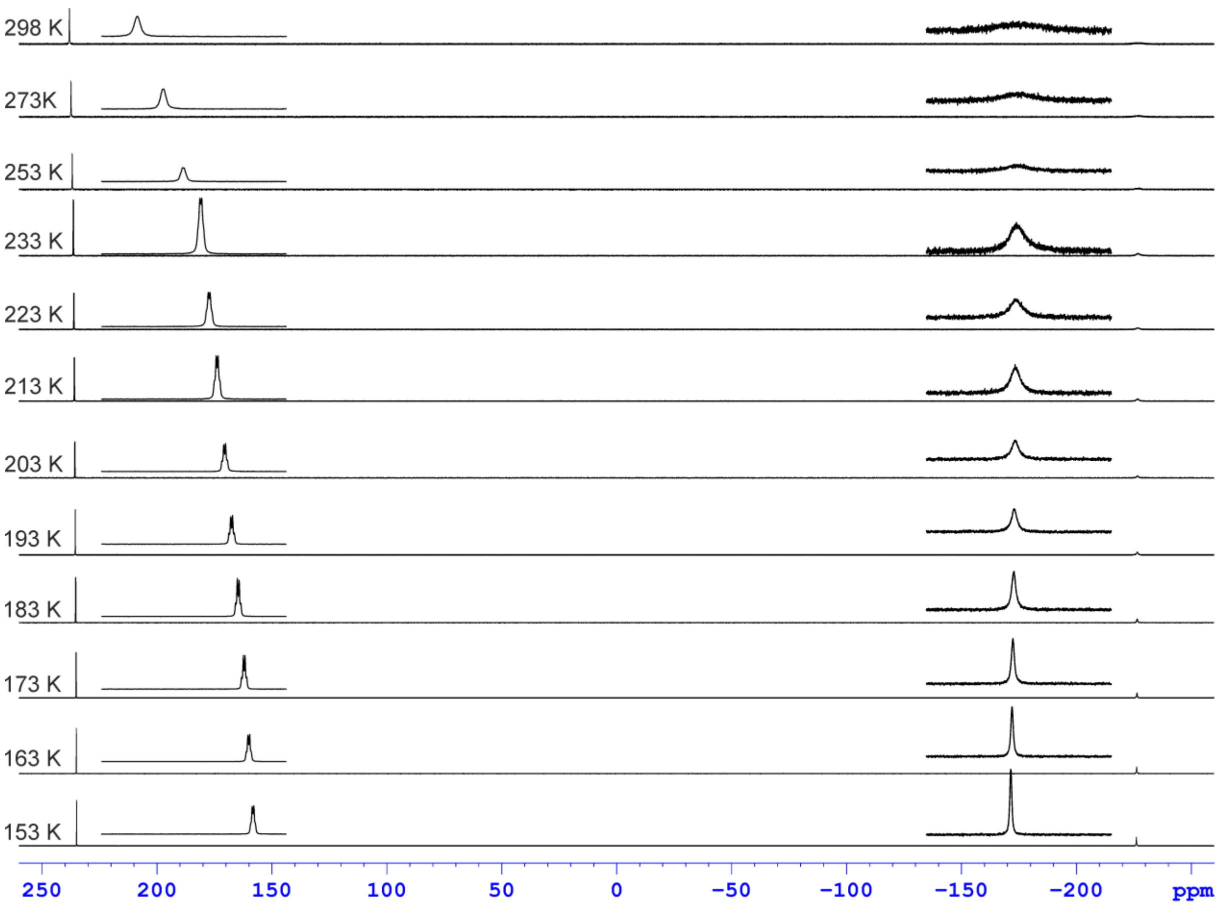

Figure S 26. $^{31}P\{^{1}H\}$ NMR spectra (161.98 MHz, THF-d_8/2-Me-THF) of isolated [Na(crypt-222)]**4** recorded at variable temperature. Inlets: Zoomed in signals.

Table S 2. Line withs and thermodynamic parameters derived from the VT NMR data for [Na(crypt-222)]**4**. The thermodynamic parameters were calculated by the Eyring equation using Microsoft Copilot (GPT-5), February 2026

**Parameter**	**δ ≈ 238 ppm**	**δ ≈ −227 ppm**
**T / K**	**Line width / Hz** [a)]	**Line with / Hz**
300	32.29	20062.4
273	29	11309.5
253	26.34	686.32
233	23.22	298.759
223	19.78	295.275
213	16.8	180.37
203	14.97	148.65
193	13.85	116.45
183	13.06	83.66
173	12.71	62.43
163	13.3	49.04
153	13.75	42.1
Intrinsic line with (Hz)	12.71	42.1
$\Delta H^{\ddagger}$ (kJ/mol) [a)]	8.92 ± 1.74	20.19 ± 1.77
$\Delta S^{\ddagger}$ (J/mol K) [a)]	-179.68 ± 8.48	-92.58 ± 7.82
$\Delta G^{\ddagger}$ (298 K; kJ/mol) [a)]	62.47 ± 3.05	47.77 ± 2.92

a) The line with of the whole multiplet has been considered (peakw command in Topspin 4.5).

2.7 Variable-temperature $^{31}P\{^1H\}$ NMR reaction monitoring of the reaction of [(η^4-P_4)Co{η^4-P_4Si(nacnac)}] (2) with KOPh (2.0 equiv.) and crypt-222 (2.0 equiv.)

[(η^4-P_4)Co{η^4-P_4Si(nacnac)}] (**2**, 14.8 mg, 0.020 mmol, 1.0 equiv.) suspended in THF-*d*$_8$ (0.3 mL) in a J. Young valve NMR tube and cooled to −80 °C. A solution of crypt-222 (14.8 mg, 0.039 mmol, 2.0 equiv.) and KOPh (5.2 mg, 0.039 mmol, 2.0 equiv.) in THF-*d*$_8$ (0.3 mL) at −80 °C was added. The $^{31}P\{^1H\}$ NMR measurements were performed in a temperature range from 193 K to 298 K in 20 K steps (Figure S27).

Initially, **2** is quickly deprotonated to form **3** (*vide supra*), which is the only phosphorus-containing product in the temperature range of 193 K to 273 K (Figure S27). At 298 K, the formation of **4** is visible. No other reaction intermediates were observed on the NMR time scale. After 3 h at room temperature, the reaction is complete, and **4** is observed as the sole phosphorus-containing product (Figure S27).

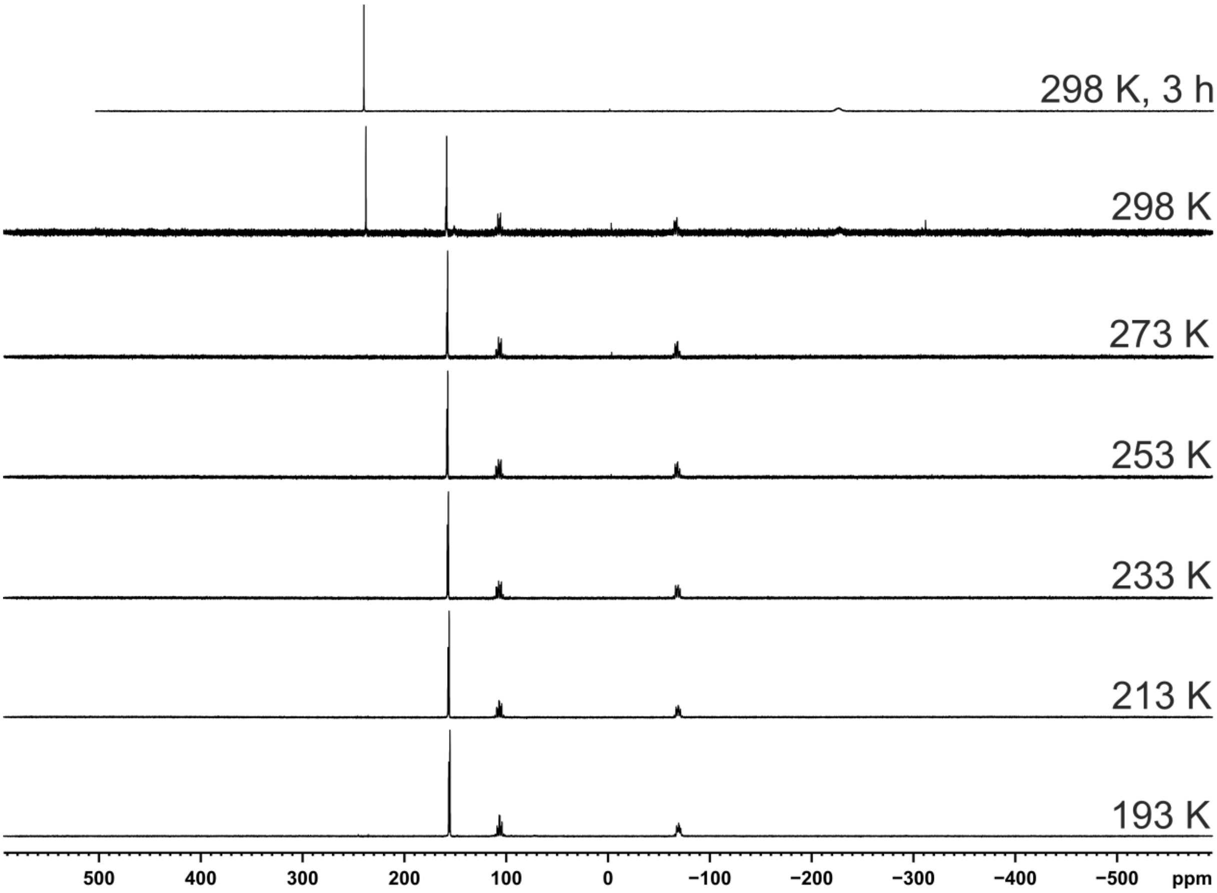

Figure S27. $^{31}P\{^1H\}$ VT NMR spectra (161.98 MHz, THF-*d*$_8$) of the reaction mixture of **2** with 2.0 equiv. KOPh and 2.0 equiv. [2.2.2]-cryptand. Note: The spectrum of the reaction mixture at 298 K after 3 h was recorded on a different 400 MHz spectrometer (162.04 MHz).

3. EPR Spectroscopic Investigations

Inside a N_2-filled glovebox, the [K(crypt-222)]$_2$**5** was added to a *J*-young quartz EPR tube. The samples were frozen in liquid N_2, before the EPR spectra were measured. At 77 K, no signal could be detected. The X-band EPR spectrum of a solid sample of [K(crypt-222)]$_2$**5**, as well as a sample prepared by dissolving solid [K(crypt-222)]$_2$**5** in a mixture of 2-MeTHF:*o*-DFB were recorded at 10 K. After addition of the solvent, the sample was frozen immediately in liquid N_2.

The EPR spectrum of solid [K(crypt-222)]$_2$**5** (powder) showed a (nearly) axial spectrum with g_{iso} = 2.03 (Figure S27). The broad signal at 120 mT (*g*-value = 5.3) stems from a minor unidentified impurity.

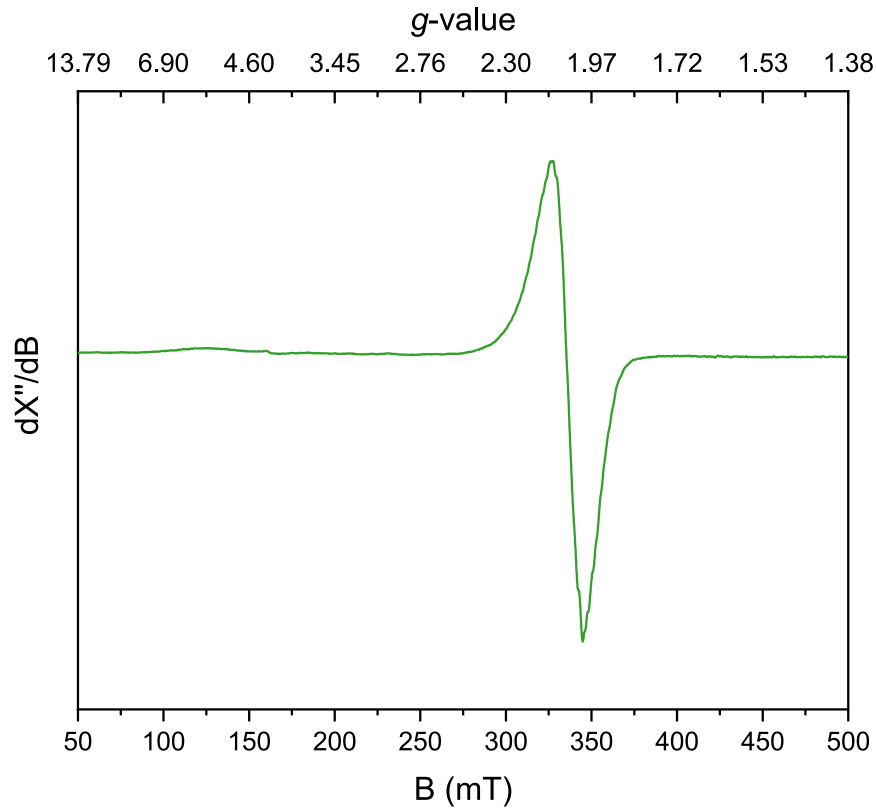

Figure S28. X-band EPR spectrum of [K(crypt-222)]$_2$**5**. Conditions: solid state (10 K). Settings: 9.651557 MHz, 6.325 mW, modulation amplitude: 8 G.

The X-band EPR spectrum of [K(crypt-222)]$_2$**5** in frozen 2-MeTHF:*o*-DFB showed a weak signal due to the low solubility of the compound. Therefore, the sample was briefly warmed up to dissolve more of the compound (brown suspension) and then frozen immediately again (Figure S28).

The obtained EPR spectrum was simulated with Easyspin,[8] using the cwEPR plugin.[9] The coupling of the radical with the cobalt and phosphorus nuclei disappear due to broadening of the signal (Figure S28). DFT calculations of the *g*-values of [K(crypt-222)]$_2$**5** using the PBE0[10,11,12]/aug-cc-pVTZ-J[13,14] level of theory revealed satisfactory matching values, indicating a satisfactory correlation to the values obtained by simulation (Table S3). The hyperfine couplings used in the simulation are not resolved. They are based on a comparison

with the DFT calculated values in combination with line shape fitting of the experimental spectrum.

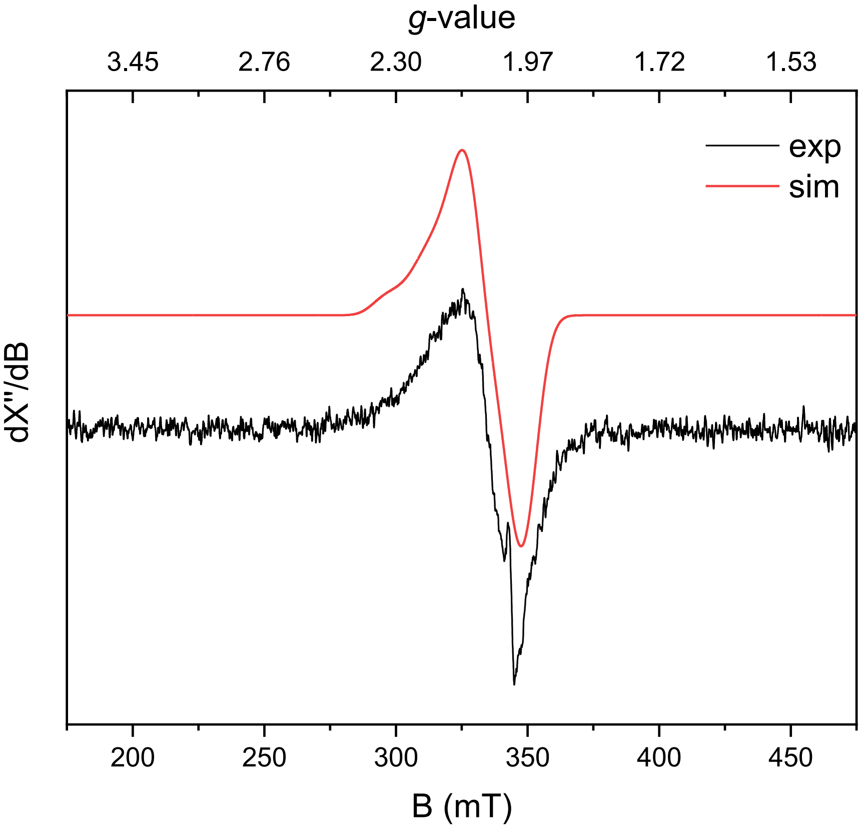

Figure S29. X-band EPR spectrum of compound [K(crypt-222)]$_2$**5** suspended in 2-MeTHF:*o*-DFB (1:1 *v*/*v*) at 10 K (black) and simulated EPR spectrum (red). Settings: 9.643337 MHz, 6.325 mW, modulation amplitude: 8 G. By using the calculated hyperfine coupling constants in the simulation, a very similar signal is obtained.

Table S3. Parameters obtained from the simulation of the X-band EPR spectrum of [K(crypt-222)]$_2$**5** in 2-MeTHF:*o*-DFB (1:1 *v*/*v*) and DFT calculations using the PBE0/aug-cc-pVTZ-J level of theory.

		**Calculated**		
	**Simulation**	**PBE0/aug-cc-pVTZ-J**	**„Turbomole-1c“**[b)]	**„Tubomole-2c“**[c)]
g_{11}	1.9841	1.9652	1.975	1.417
g_{22}	2.0832	2.0036	2.007	2.096
g_{33}	2.17	2.0227	2.036	2.349
S	0.5			
lwpp	9.0992			
A^{Co} (MHz)	−1.4, 29, 139	14.6, 39.6, 144.7 a)		
A^{P} (MHz)	−86, −93, 475	−128.6, −139.1, 361.8		

a) The same line shape is obtained when these coupling constants are used in the simulation, without fitting.
b) Turbomole relativistic ($x2c, $rlocal, $finnuc, $snso) 1-component EPR calculations (b3-lyp functional, x2c-TZVPall-2c basis). See Turbomole 7.9.0 manual for details.
c) Turbomole relativistic ($x2c, $rlocal, $finnuc) 2-component EPR calculations (b3-lyp functional, x2c-TZVPall-2c basis). See Turbomole 7.9.0 manual (gtensprep.sh -msnso -hfc -efg) for details.

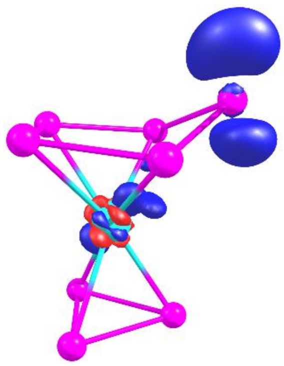

Figure S30. Spin density distribution of **5** in the doublet spin state calculated at the PBE0/aug-cc-pVTZ-J level of theory.

4. Cyclic Voltammetry

Cyclic voltammetry was performed in a borosilicate glass single-compartment cell inside a nitrogen-filled glovebox using a CH Instruments CHI600E potentiostat. A platinum disc working electrode (1.6 mm diameter) was polished with 0.1 µm diamond paste, rinsed with acetone and water, then polished with 0.05 µm alumina paste, rinsed, and gently dried with a hot-air blower. The platinum wire counter electrode was rinsed with water and acetone before use and then annealed with a propane torch until no orange flame tail was observed, indicating complete combustion of organic residues. The silver wire pseudo-reference electrode was polished sequentially with 7000- and 10000-grit sandpaper, wiped with a lint-free tissue to remove residual solids, and rinsed with water and acetone. Residual solvent on the electrodes was removed under vacuum in the glovebox antechamber. The working and counter electrodes shared the same compartment, and the applied voltage was not corrected for IR drop. The supporting electrolyte, [$^{n}Bu_4N$]PF_6 [(TBA)PF_6], was dried under vacuum (10^{-3} mbar) at 110 °C for three days. All redox potentials are referenced to the ferrocene/ferrocenium (Fc/Fc^+) couple. CV scans were recorded at a scan rate of 100 mV/s.

A solution of [Na(crypt-222)]**4** (100 µL, 10 mM in THF) was added to a three electrode CV cell containing a THF solution of the supporting electrolyte (500µL, (TBA)PF_6, 0.2 M in THF). The initial potential was set to 0.00 V and scanned in the reductive direction (Figure S31). At $E_{p,c}$ = −2.8 V, an irreversible process occurs, which we attribute to the reduction of **4** to **5**. A similar reduction at a cathodic peak potential of $E_{p,c}$ = −2.05 V was reported for [Cp*Fe(η5-P_5)].[15,16] At an anodic peak potential of $E_{p,a}$ = −1.45 V, an oxidation wave is observed, which is attributed to the oxidation of species formed during the reduction of **4** (*i.e.* the reduction event at $E_{p,c}$ = −2.8 V). Scanning only the range from −1.0 V to −2.0 V does not show the wave at $E_{p,a}$ = −1.45 V. The irreversible oxidation process at $E_{p,a}$ = −0.55 V likely corresponds to the oxidation of **4**, since it is independent of both the reduction at $E_{p,c}$ = −2.8 V and the oxidation process at $E_{p,a}$ = −1.45 V. Note that dissolving solid [K(crypt-222)]$_2$**5** in THF or in a mixture of THF/*o*-$C_6F_2H_4$ results in fast decomposition (*c.f.* EPR spectroscopic investigations).

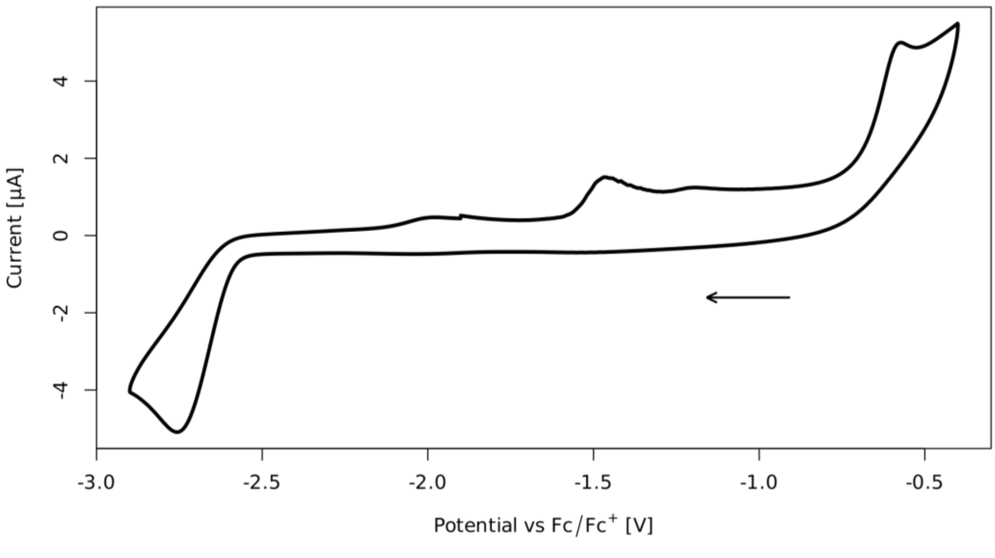

Figure S31. Cyclic voltamogramm of [Na(crypt-222)]**4** (1 mM) recorded in THF containing 0.2 M (TBA)PF_6 as supporting electrolyte at a scan rate of 0.1 mV s^{-1}. The arrow indicates the starting potential and the scan direction.

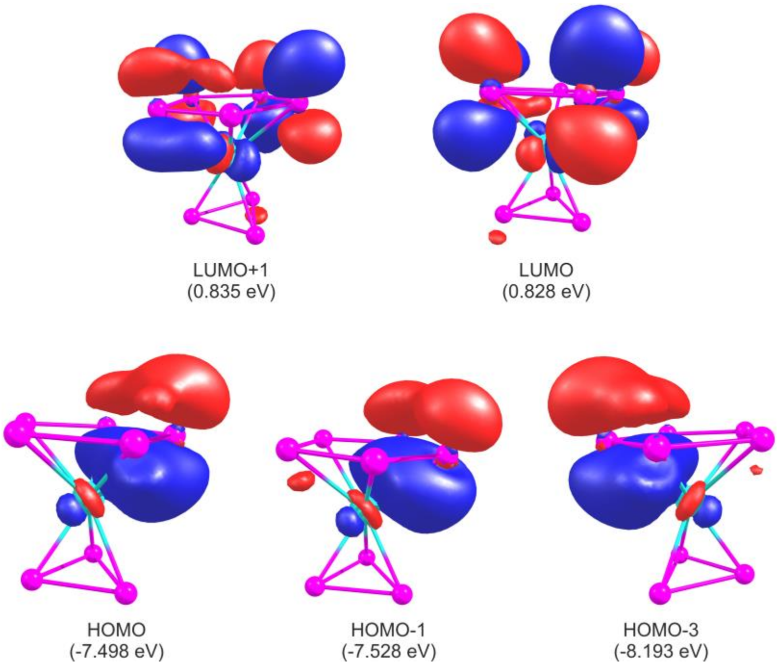

Figure S32. Frontier canonical molecular orbitals of **4**, calculated at the ωB97X-D4/def2-TZVP level of theory.

5. DFT calculations

Magnetic Shielding

All calculations were carried out on Gaussian09.[17] The geometry optimizations of the complexes were performed with B3LYP using Grimme's D3 dispersion correction[18] with the 6-31+G(d,p) basis set for all non-metal atoms and Ahlrich's def2-TZVP[19] basis set for cobalt. The implicit polarisable continuum model (PCM) for the relevant solvent (tetrahydrofuran unless otherwise specified) was used in all calculations, and the anionic complexes were modelled without the countercations. The reported free energies were calculated at 298 K and 1 atm.

The magnetic shielding of the phosphorus atoms was calculated using the PBE0 functional and pcSseg-3[20], a polarization-consistent basis set with segmented contraction, with the GIAO method. This basis set has been used in previous work with ^{31}P NMR.[21] The 6-31G(d) basis set was used for all other non-metal atoms in the reference compounds, and the def2-TZVP basis set was used for cobalt. A dataset with the calculations listed in this manuscript has been made freely available on ioChem-BD[22] with DOI: 10.19061/iochem-bd-1-411.

The chemical shifts of the phosphorus atoms in the complexes were referenced by scaling to a linear regression of the magnetic shielding of five compounds calculated at the same level of theory: PH_3, PMe_2H, PMe_3, PCl_3, and PF_3 (Table S4). Scaling to a linear regression has proven to be a valid way to predict ^{31}P chemical shifts and mitigates the impact of systematic errors compared to a single-point reference.[23] The calculated chemical shifts are displayed in Table S4.

The computational data are available electronically via the following link: https://doi.org/10.19061/iochem-bd-1-411.

Table S4. Experimental chemical shift and shielding of the phosphorus compounds used for linear regression.

	solvent	Experimental ^{31}P δ/ppm		σ_{iso} (shielding)[a]
PH_3	gas phase	−266.1	Ref. [24]	572.0657
PMe_2H	THF	−98.5	Ref. [25]	412.1227
PMe_3	C_6D_6	−62.3	Ref. [26]	367.2047
PCl_3	gas phase	217	Ref. [27]	3.0165
PF_3	CD_3CN	103.1	Ref. [28]	165.2404

[a] Geometry optimisation: B3LYP-D3/6-31+G(d,p); NMR: PBE0-D3/pcSseg-3(P) + 6-31G(d) (C,H,F,Cl). Linear regression: -0.8387σiso + 233.54 (R2 = 0.9929).

Table S5. Calculated and experimental ^{31}P NMR chemical shifts of **4**.

	calcd. ^{31}P δ / ppm	exp ^{31}P δ / ppm (at 298 K)
$\delta(P_A)$	155.2	175.0
$\delta(P_{MM'})$	130.6	111.3
$\delta(P_{XX'})$	−102.1	−86.0

To assess the aromaticity of the P_3 ligand, we performed a NICS scan starting from the ring centroid and along its normal vector (at 0.0 – 3.0 Å), extracting both NICS and $NICS_{ZZ}$ (Figure S33).[29,30] The calculations were performed in ORCA 6.1 with the GIAO method at the

PBE0-D4/def2-QZVPPD level of theory on geometries optimized at TPSSh-D4/def2-TZVPPD,[31–34] in both cases with SMD solvation[35] for tetrahydrofuran and VeryTightSCF convergence criteria. The obtained chemical shielding values were validated by crosschecking the results with TPSSh-D4 and B3LYP-D4 (Tables S6 and S7). The very large NICS(0) value, at –56.0 ppm, is dominated by the out-of-plane component, with the NICS(0)$_{ZZ}$ of –58.8 ppm, although presumably influenced by the proximity of the metal atom. While NICS(1) is lower, at –24.0 ppm, the corresponding NICS(1)$_{ZZ}$ remains very large at –50.5 ppm, suggesting a very strong diatropic field that extends from the P_3 ring and in line with π aromaticity. Local shielding extends also at longer ranges, with a NICS(3)$_{ZZ}$ of –11.5 ppm. As local effects from σ electrons typically decay rapidly with distance, these results are consistent with strong π contributions, which would be related to the delocalized three-center NBO. In order to account for possible confounding effects due to the metal, we also carried out the NICS calculations on the $(P_3)^+$ ring extracted from the complex (Table S8). The corresponding 2π model system showed a NICS(1) of –20.0 ppm only slightly dropping to a NICS(0) of –18.2 ppm, suggesting there is a degree of additional metal-related shielding near the ring centroid, making NICS(1) and NICS$_{ZZ}$ more reliable indicators. The $(P_3)^+$ ring showed a NICS$_{ZZ}$ pattern very similar to that of the cobalt complex (with a large NICS(0)$_{ZZ}$ of –45.5 ppm) and, thus, the observed features appear to be intrinsic to the P_3 ring (Figure S34). These results point to strong magnetic aromaticity and a description of the P_3 ligand as a 2-electron donor LZ-type ligand, consistent both with NBO analysis and with other investigations into the bonding of P_3.[36]

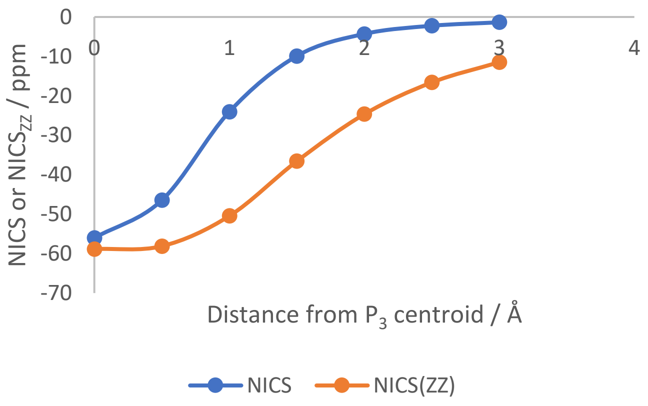

Figure S33. NICS and NICS$_{ZZ}$ values at different distances from the P_3 ring of $[(P_5)Co(P_3)]^-$, PBE0-D4/def2-QZVPPD, SMD (THF).

Table S 6. NICS values at different distances from P_3 ring of $[(P_5)Co(P_3)]^-$, geometry optimised at TPSSh-D4/def2-TZVPPD, SMD (THF), DefGrid3 (VeryTightSCF, VeryTightOpt). Values in ppm reported at functional/def2-QZVPPD, SMD(THF), DefGrid3.

NICS	B3LYP-D4	PBE0-D4	TPSSh-D4
0.0 Å	-54.8	-56.0	-54.4
0.5 Å	-45.4	-46.5	-45.0
1.0 Å	-23.7	-24.0	-23.2
1.5 Å	-9.9	-9.9	-9.6
2.0 Å	-4.4	-4.3	-4.2
2.5 Å	-2.3	-2.2	-2.2
3.0 Å	-1.4	-1.4	-1.3

Table S 7. $NICS_{ZZ}$ values at different distances from P_3 ring of $[(P_5)Co(P_3)]^-$, geometry optimised at TPSSh-D4/def2-TZVPPD, SMD (THF), DefGrid3 (VeryTightSCF, VeryTightOpt). Values in ppm reported at functional/def2-QZVPPD, SMD(THF), DefGrid3.

NICS	B3LYP-D4	PBE0-D4	TPSSh-D4
0.0 Å	-58.0	-58.8	-59.1
0.5 Å	-57.2	-58.1	-57.1
1.0 Å	-49.6	-50.5	-49.1
1.5 Å	-36.0	-36.6	-35.6
2.0 Å	-24.4	-24.7	-24.1
2.5 Å	-16.4	-16.6	-16.3
3.0 Å	-11.4	-11.5	-11.3

The features observed in the NICS calculations of $(P_3)^+$ were essentially identical regardless of whether the same bond lengths as those in the complex were preserved or the $(P_3)^+$ geometry was first optimized on its own at the same level of theory (TPSSh-D4/def2-TZVPPD). The NICS values as well as the side-by side comparison with the optimized geometry are shown in Table S 8.

Table S 8. NICS values at different distances from the $(P_3)^+$ ring, both with the same geometry as in the metal complex (unrelaxed) and re-optimized at TPSSh-D4/def2-TZVPPD, SMD (THF), DefGrid3. Values in ppm reported at PBE0-D4/def2-QZVPPD, SMD(THF), DefGrid3.

Unrelaxed	NICS	$NICS_{ZZ}$	Optimized	NICS	$NICS_{ZZ}$
0.0 Å	-18.2	-45.5	0.0 Å	-18.5	-45.7
0.5 Å	-22.9	-47.0	0.5 Å	-23.6	-47.8
1.0 Å	-20.0	-40.8	1.0 Å	-20.4	-41.6
1.5 Å	-11.9	-28.4	1.5 Å	-11.8	-28.8
2.0 Å	-6.8	-18.2	2.0 Å	-6.6	-18.2
2.5 Å	-4.2	-11.6	2.5 Å	-4.1	-11.6
3.0 Å	-2.8	-7.7	3.0 Å	-2.7	-7.6

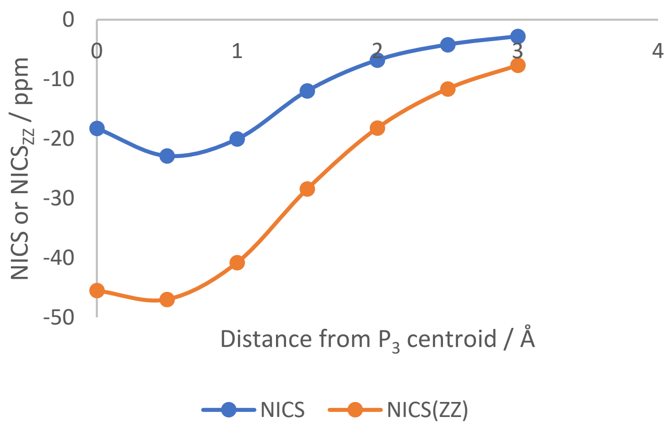

Figure S 34. NICS and $NICS_{ZZ}$ values at different distances from the $(P_3)^+$ ring, PBE0-D4/def2-QZVPPD, SMD (THF).

NBO calculations (TPSSh-D4/def2-QZVPPD, SMD (THF), DefGrid3) on the optimized geometry, with an underlying Lewis structure based on separated $(P_5)^-$, $(P_3)^+$ and Co^-, show that the π electrons of the P_3 ring are shared evenly among the three phosphorus atoms (occupancy 1.81 and >92% p character), the doubly degenerate antibonding combinations having an NBO occupancy of 0.66. The Co d orbital-based NLMOs (62 and 63) showed a 19.7% contribution from the P_3 ring, practically entirely through the π system, and the P3 three-center π NLMO (74) showed a 10.2% contribution from the Co s orbital. From second-order perturbation theory analysis, bonding and backbonding in the π systems of P5 and P3 showed strong donation and backdonation, with particularly strong delocalization of metal orbitals onto the P_3 ring (Table S 9). The absolute values of the totals, while sometimes broadly method-dependent, are dominated by a few strong interactions.

Table S 9. Sum of the delocalization of ligand π system NBOs onto the empty cobalt s orbital and of cobalt d orbitals onto π* NBOs of the ligands. Contributions below 2 kcal mol^{-1} neglected.

	**L (π) to Co (s) / kcal mol^{-1}**	**Co (d) to L(π*) / kcal mol^{-1}**
**P_5**	231.6	45.6
**P_3**	202.0	79.9

6. X-ray Crystallography

Crystallographic data were recorded on a Rigaku XtaLAB Synergy DW system with a HyPix-Arc 150° detector using Cu-K_α radiation (λ = 1.54184 Å). Crystals were selected under mineral oil, mounted on micromount loops and quench-cooled using an Oxford Cryosystems open-flow N_2 cooling device. The diffraction pattern was indexed, and the total number of runs and images was determined using the strategy calculation in the program CrysAlisPro (Rigaku, V1.171.41_64.93a, 2020 or V1.171.42.53a, 2023). The unit cell was refined using the same program. Either semi-empirical multi-scan absorption corrections[37,38] or analytical[39] ones were applied to the data. Using Olex2,[40] the structures were solved with SHELXT[41] using intrinsic phasing and refined with SHELXL,[42] or olex2.refine[43] using least-squares refinement on F^2. The hydrogen atoms were located in idealized positions and refined isotropically with a riding model.

6.1 Refinement of the solid-state structure of [Na(crypt-222)][(η^5-P_5)Co(η^3-P_3)] ([Na(crypt-222)]4)

Single crystals of [Na(crypt 222)]**4** were obtained from a THF/toluene solution at −35°C.

Refinement using NoSpherA2, an implementation of non-spherical atom-form-factors in Olex2.[44] NoSpherA2 implementation of Hirshfeld Atom Refinement (HAR) uses tailor-made aspherical atomic form factors calculated on-the-fly from a Hirshfeld-partitioned electron density (ED) - not from spherical-atom form factors.The electron density is calculated from a Gaussian basis set single determinant SCF wavefunction (either obtained from Hartree-Fock or DFT calculations) for a fragment of the crystal. This fragment can be embedded in an electrostatic crystal field using cluster charges or modelled with implicit solvation models, depending on the software used.

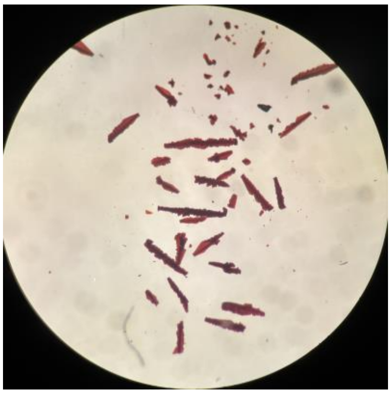

Figure S35. Photograph of crystals of ([Na(crypt-222)]**4** obtained from a toluene:THF (1:1 *v*:*v*) solution at −35°C.

The bonding situation in **4**$^-$ was further analyzed using Hirshfeld atom refinement on the X-ray diffraction data of ([Na(crypt-222)]**4**.[45,46] Non-spherical, structurally individual atomic form factors were calculated by NoSpherA2 in Olex2[43,44] based on a molecular wavefunction on the crystallographic geometry calculated by ORCA (version 5)[47–49] and then used iteratively to update geometry and the wavefunction until a convergence was reached after four cycles. The

molecular wavefunction was based on the asymmetric unit in [Na(crypt-222)]**4** and calculated using the wB97X-V functional,[50] the def2-TZPP basis set for all atoms,[51] "NoSpherA2SCF" as the SCF threshold, a "NormalConv" convergence strategy, and an H_2O implicit solvent model to imitate crystal effects.[52]

The HAR model was analyzed by means of the electron deformation density ($F^{calc(HAR)}$-$F^{calc(IAM)}$), the Laplacian of the total electron density ($\nabla^2\sigma$), and Bader charges, and directly compared to previous studies on [Cp'''Ni(η^3-P_3)],[36,53] sharing a total number of valence electrons and isolobal (*cyclo*-P_5 ↔ Cp''') and identical (*cyclo*-P_3) ligands. The investigation showed remarkable similarities between the deformation density and the Laplacian (Figure S35) with the previously reported experimental charge-density studies and experimental wavefunction fitting for [Cp'''Ni(η^3-P_3)]. The similarities include direct M–P bonding in the cyclo-P_3 moiety, rather than an aromatic multicenter coordination; lone pairs on each P atom in the cyclo-P_3 ring; and P–P bonds bent outward from the *cyclo*-P_3 center.

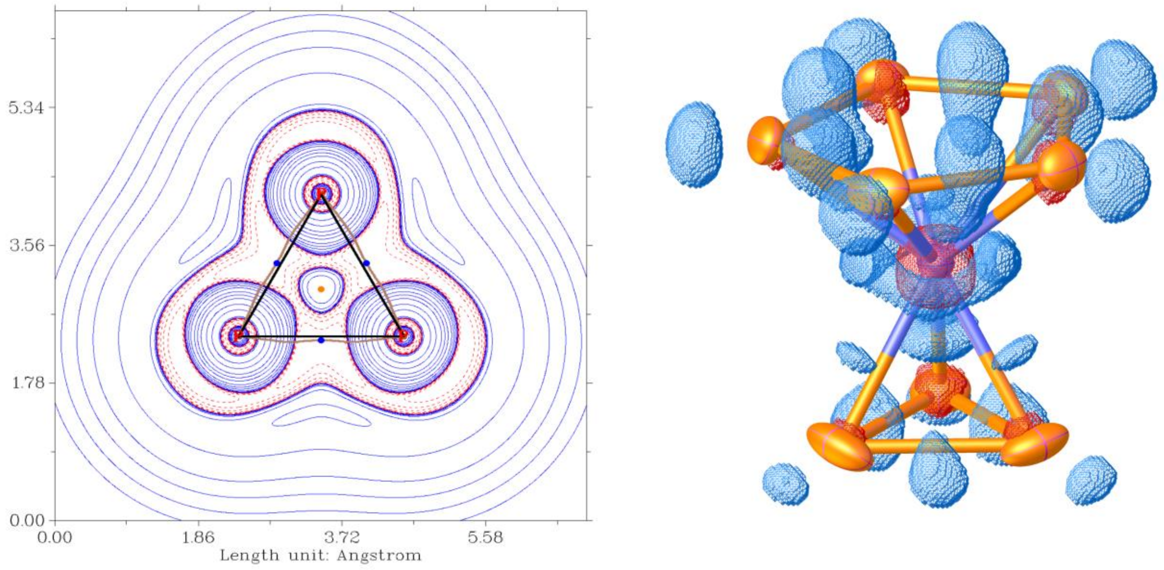

Figure S36. Laplacian of the electron density of the *cyclo*-P_3 ring in **4**$^-$ calculated using the HAR model (left) and electron deformation density (right).

6.2 Refinement of the solid-state molecular structure of [K(crypt-222)]$_2$[(η^4-P_5)Co(η^3-P_3)] ([K(crypt-222)]$_2$**5**)

All crystallization attempts afforded only weakly diffracting yellow to orange plates, usually grown together as red flower-like crystals (for crystals obtained from a Tol:THF (1:1 v/v) mixture, see Figure S31). The measured crystal was obtained from a saturated *o*-DFB solution layered with toluene, with diethyl ether slowly diffusing simultaneously (see Figure S37). The crystal's diffraction limit was determined to be 0.82 Å in CrysAlisPro. Data below this limit was not included in the final refinement. [K(crypt-222)]$_2$**5** crystallizes in the triclinic space group P-1 with two [K(crypt-222)]$^+$ cations and one [Co(η^4-P_5)(η^3-P_3)]$^{2-}$ dianion in the asymmetric unit. The disorder of the *cyclo*-P_3 ring was modeled in an 81:19 ratio. The ISOR 0.02 0.05 restraint was used for the P10 atom. The modeled disorder is presented in Figure S34.

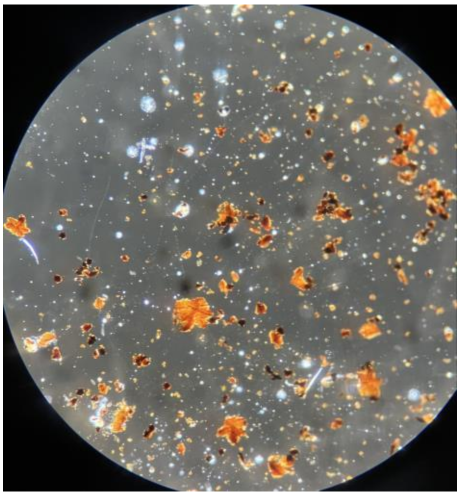

Figure S37. Photograph of crystals of ([K(crypt-222)]$_2$**5** obtained from a toluene:THF (1:1 *v*:*v*) solution.

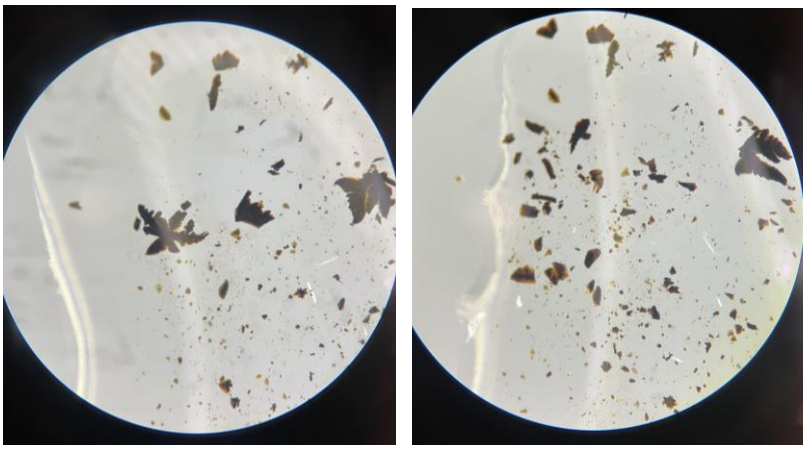

Figure S38. Photograph (without polarization filter) of crystals of ([K(crypt-222)]$_2$**5** obtained from a saturated *o*-DFB solution, layered with toluene and simultaneous diethyl ether slow diffusion. Left: dark red flower-like crystals of ([K(crypt-222)]$_2$**5**. Right: broken crystals down reveal yellow to orange plates.

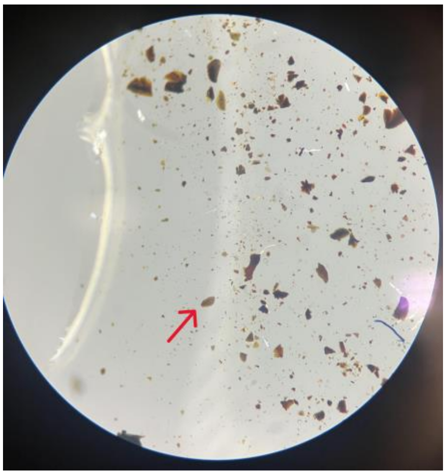

Figure S39. Photograph of the measured crystal of [K(crypt-222)]$_2$**5** (after the measurement) obtained from a saturated *o*-DFB solution, layered with toluene and simultaneous diethyl ether slow diffusion.

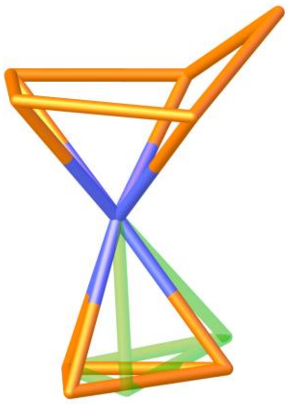

Figure S40. Wireframe representation of the molecular structure of ([K(crypt-222)]$_2$**5**. The disordered part (PART-1) is transparent.

6.3 Refinement of the solid-state molecular structure of [K(DME)$_3$][Co{η^4-P$_4$Si(nacnac′)}$_2$] ([K(DME)$_3$]1)

[K(DME)]**1** crystallizes as a separated ion pair, where three DME molecules coordinate the potassium counter ion. In the anion, two silatetraphosphacyclopentadiene rings featuring doubly deprotonated nacnac′ ligands are present, which are both η^4-coordinated to the central cobalt atom (Figure S35). The nacnac′ fragments show orientational disorder, which is common for this type of ligand.[3,7,54] Thus, the C=C double bonds are disordered over two positions (C1–C2 and C4–C5) in the crystal.

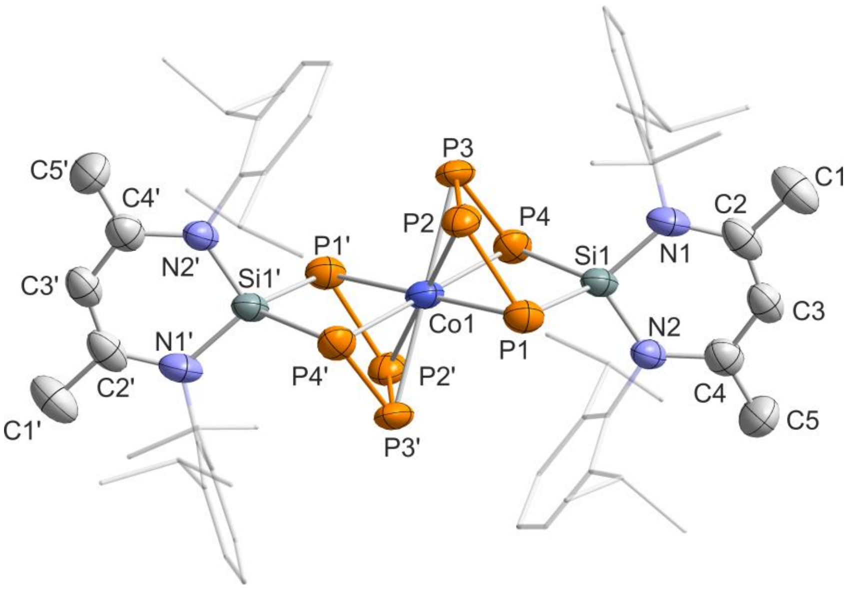

Figure S41. Solid-state molecular structure of [K(DME)$_3$]**1**. Hydrogen atoms, counterion, solvate molecules, and disorders are omitted for clarity. Thermal ellipsoids are drawn at the 40% probability level. The crystal of [K(DME)$_3$]**1** contains a second crystallographically independent molecule with very similar structural parameters; only one of these molecules is shown. Selected bond lengths [Å] and angles [°] (in case of disorder bond lengths and angles are given for the part with the highest occupancy): P1–P2 2.133(2), P1–P4 3.275(2), P2–P3 2.162(2), P3–P4 2.127(2), Si1–P1 2.233(2), Si1–P4 2.222(1), Co1–P1 2.404(1), Co1–P2 2.309(1), Co1–P3 2.332(1), Co1–P4 2.391(1), C1–C2 1.453(6), C4–C5 1.421(7), P1–P2–P3 105.49(7), P2–P3–P4 104.79(6), P3–P4–Si1 100.67(6), P2–P1–Si1 100.40(6), P1–Si1–P4 94.64(6).

Several crystallization efforts only afforded weakly diffracting crystals. All tested crystals showed disorder in both the phosphorus framework and the counterion. The addition of crown ethers did not improve the disorders. Suitable crystals were obtained by crystallizing **4** from a concentrated DME solution layered with n-hexane. $[K(DME)_3]$**1** crystallizes in the triclinic space group P-1 with one cation and two times half of the anionic molecule in the asymmetric unit. The disorder of the DME molecules around the potassium cation was treated with strong restraints (SIMU, DFIX) to model a chemically sensible coordination. Disorder of the phosphorus framework was treated with the SIMU restraint. One of the [Si(nacanc′)] moieties was additionally split into two parts to maintain a reasonable connectivity to the *catena*-P_4 moiety. The modelled disorders are presented in Figure S36. Due to the disorder, some uncertainty remains in the structural parameters, especially within the phosphorus framework.

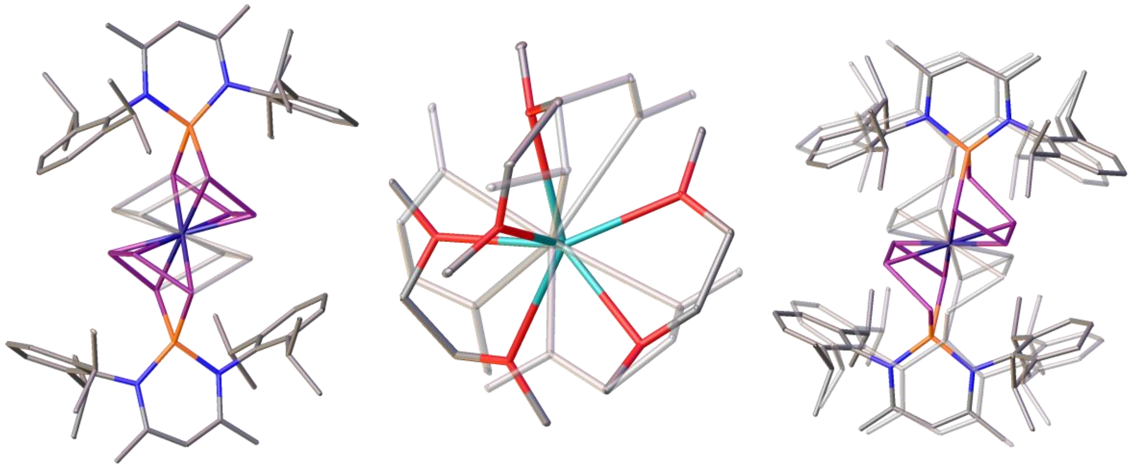

Figure S42. Wire/Stick representation of extracts of the grown solid-state molecular structure of $[K(DME)_3]$**1**. Hydrogen atoms are omitted for clarity. Disordered parts are transparent (PART 2).

6.4 Refinement of the solid-state structure of [(η^4-P_4)Co{η^4-P_4Si(nacnac)}] (2)

Compound **2** was crystallized from a CH_2Cl_2 solution layered with hexane at −35°C as **2**·CH_2Cl_2. The crystals rapidly lose the incorporated dichloromethane, and hence only poor-quality data could be obtained. Therefore, only preliminary X-ray data is reported. A solvent mask was calculated, and 176 electrons were found in a volume of 612Å^3 in 1 void per unit cell. This is consistent with the presence of one CH_2Cl_2 molecule in the asymmetric unit, which accounts for 168 electrons per unit cell.

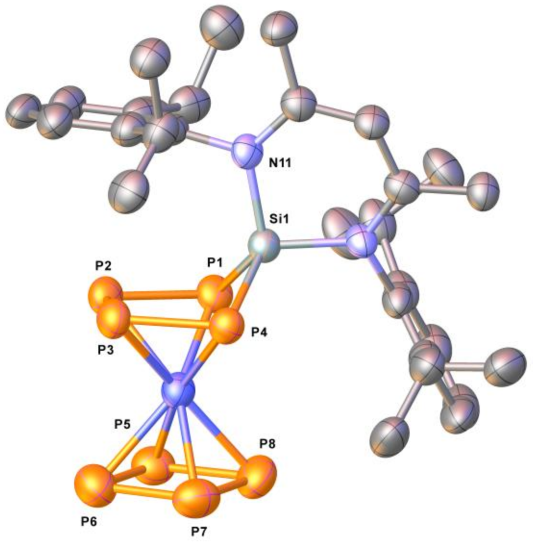

Figure S43. Solid-state molecular structure of **2**. Ellipsoids at 40 % probability. Hydrogen atoms are omitted for clarity. Selected bond lengths [Å] and angles: Co9-P1 2.364(2), Co9-P2 2.298(2), Co9-P3 2.2911(19), Co9-P4 2.3956(19), Co9-P5 2.329(2), Co9-P6 2.330(2), Co9-P7 2.279(2), Co9-P8 2.284(2), P1-P2 2.139(2), P1-Si1 2.210(2), P2-P3 2.159(3), P3-P 2.138(2), P4-Si1 2.202(2), P5-P6 2.156(3), P5-P8 2.184(3), P6-P7 2.141(3), P7-P8 2.170(3).

Table S10. Crystallographic data for compounds [Na(crypt-222)]**4,** ([K(crypt-222)]$_2$**5**, [K(DME)$_3$]**1** and **2**.

**Compound**	[K(DME)$_3$]**1**	**2**	[Na(crypt-222)]**4**	[K(crypt-222)]$_2$**5**
Formula	$C_{140}H_{220}Co_2K_2N_8O_{12}P_{16}Si_4$	$C_{30}H_{43}Cl_2CoN_2P_8Si$	$C_{18}H_{36}CoN_2NaO_6P_8$	$C_{36}H_{72}CoK_2N_4O_{12}P_8$
μ/mm^{-1}	4.315	8.392	8.693	6.647
Formula Weight	3011.17	837.403	706.209	1137.913
Size/mm	0.41×0.34×0.20	0.11×0.10×0.04	0.06×0.05×0.04	0.13×0.06×0.04
T/K	123.01(10)	123.00(10)	123.00(10)	123.00(10)
Crystal System	triclinic	monoclinic	monoclinic	triclinic
Space Group	P-1	$P2_1/c$	$C2/c$	P-1
a/Å	15.1112(6)	12.3433(4)	22.1210(3)	11.0883(3)
b/Å	15.9506(6)	18.7734(5)	17.3937(2)	11.5029(3)
c/Å	20.0778(8)	17.0709(5)	16.1493(2)	21.1237(6)
α/°	90.376(3)	90	90	86.001(2)
β/°	110.837(4)	102.557(3)	92.863(1)	84.852(2)
γ/°	115.288(4)	90	90	88.394(2)
V/Å^3	4016.9(3)	3861.1(2)	6205.95(13)	2676.23(13)
Z	1	4	8	2
Z'	0.5	1	1	1
Wavelength/Å	1.54184	1.54184	1.54184	1.54184
Radiation type	Cu K$_a$	Cu K$_a$	Cu K$_a$	Cu K$_a$
Q_{min}/°	3.386	3.55	3.23	3.85
Q_{max}/°	76.358	65.08	75.56	67.08
Index range h	-15 ≥ h ≥ 18	-14 ≥ h ≥ 14	-27 ≥ h ≥ 27	-13 ≥ h ≥ 13
Index range k	-20 ≥ k ≥ 19	-21 ≥ k ≥ 22	-20 ≥ k ≥ 21	-14 ≥ k ≥ 13
Index range l	-24 ≥ l ≥ 25	-20 ≥ l ≥ 19	-18 ≥ l ≥ 20	-26 ≥ l ≥ 26
Measured Refl's.	33164	98088	29343	39582
Indep't Refl's	16364	6587	6296	9525
Refl's I≥2s(I)	13630	5318	5690	6776
R_{int}	0.0485	0.1352	0.0190	0.0788
Parameters	1368	380	709	587
Restraints	1490	0	0	18
Largest Peak/eÅ^3	0.836	1.359	0.4129	1.0783
Deepest Hole/eÅ^3	-0.625	-0.747	-0.3223	-1.0682
GooF	1.031	1.056	1.0403	1.0031
R_1 (I≥2s(I) / all)	0.0846 / 0.0949	0.0861 / 0.0968	0.0198 / 0.0241	0.0854 / 0.1074
wR_2 (I≥2s(I) / all)	0.2391 / 0.2530	0.2579 / 0.2679	0.0368 / 0.0378	0.2490 / 0.2707
CCDC	2521085	2521087	2521084	2521086

7. References

(1) Jonas, K.; Mynott, R.; Krüger, C.; Sekutowski, J. C.; Tsay, Y.-H. Bis(η-1,5-Cyclooctadiene)Cobaltlithium. *Angew. Chem. Int. Ed.* **1976**, *15* (12), 767–768. https://doi.org/10.1002/anie.197607671.

(2) Driess, M.; Yao, S.; Brym, M.; van Wüllen, C.; Lentz, D. A New Type of N-Heterocyclic Silylene with Ambivalent Reactivity. *J. Am. Chem. Soc.* **2006**, *128* (30), 9628–9629. https://doi.org/10.1021/ja062928i.

(3) Ziegler, C. G. P.; Taube, C.; Kelly, J. A.; Hierlmeier, G.; Uttendorfer, M.; Weigand, J. J.; Wolf, R. An Unusual $Ni_2Si_2P_8$ Cluster Formed by Complexation and Thermolysis. *Chem. Commun.* **2020**, *56* (90), 14071–14074. https://doi.org/10.1039/D0CC05365A.

(4) Budzelaar, P. H. M. *IvorySoft: gNMR for Windows, NMR Simulation Program*; 2006.

(5) Butovskiy, M. V.; Balázs, G.; Bodensteiner, M.; Peresypkina, E. V.; Virovets, A. V.; Sutter, J.; Scheer, M. Ferrocene and Pentaphosphaferrocene: A Comparative Study Regarding Redox Chemistry. *Angew. Chem. Int. Ed.* **2013**, *52* (10), 2972–2976. https://doi.org/10.1002/anie.201209329.

(6) Azhakar, R.; Ghadwal, R. S.; Roesky, H. W.; Granitzka, M.; Stalke, D. Reactivity Studies of a Stable N-Heterocyclic Silylene with Triphenylsilanol and Pentafluorophenol. *Organometallics* **2012**, *31* (15), 5506–5510. https://doi.org/10.1021/om300476q.

(7) Ziegler, C. G. P. Heterobimetallic Polyphosphido Complexes as Useful Synthons for the Activation and Functionalization of White Phosphorus. Dissertation, Universität Regensburg, Regensburg, **2021**.

(8) Stoll, S.; Schweiger, A. EasySpin, a Comprehensive Software Package for Spectral Simulation and Analysis in EPR. *J. Magn. Res.* **2006**, *178* (1), 42–55. https://doi.org/10.1016/j.jmr.2005.08.013.

(9) Casey, T.; cwEPR, MATLAB Central File Exchange. **2024**.

(10) Perdew, J. P.; Wang, Y. Accurate and Simple Analytic Representation of the Electron-Gas Correlation Energy. *Phys. Rev. B* **1992**, *45* (23), 13244–13249. https://doi.org/10.1103/PhysRevB.45.13244.

(11) Perdew, J. P.; Burke, K.; Ernzerhof, M. Generalized Gradient Approximation Made Simple. *Phys. Rev. Lett.* **1996**, *77* (18), 3865–3868. https://doi.org/10.1103/PhysRevLett.77.3865.

(12) Perdew, J. P.; Ernzerhof, M.; Burke, K. Rationale for Mixing Exact Exchange with Density Functional Approximations. *J. Chem. Phys.* **1996**, *105* (22), 9982–9985. https://doi.org/10.1063/1.472933.

(13) Provasi, P. F.; Sauer, S. P. A. Optimized Basis Sets for the Calculation of Indirect Nuclear Spin-Spin Coupling Constants Involving the Atoms B, Al, Si, P, and Cl. *J. Chem. Phys.* **2010**, *133* (5), 054308. https://doi.org/10.1063/1.3465553.

(14) Hedegård, E. D.; Kongsted, J.; Sauer, S. P. A. Optimized Basis Sets for Calculation of Electron Paramagnetic Resonance Hyperfine Coupling Constants: Aug-Cc-pVTZ-J for the 3d Atoms Sc–Zn. *J. Chem. Theory Comput.* **2011**, *7* (12), 4077–4087. https://doi.org/10.1021/ct200587k.

(15) Winter, R. F.; Geiger, W. E. Electron-Transfer Properties of Cp^*FeP_5: Evidence for Dimerization Reactions Following Both Oxidation and Reduction. *Organometallics* **1999**, *18* (10), 1827–1833. https://doi.org/10.1021/om9809724.

(16) Butovskiy, M. V.; Balázs, G.; Bodensteiner, M.; Peresypkina, E. V.; Virovets, A. V.; Sutter, J.; Scheer, M. Ferrocene and Pentaphosphaferrocene: A Comparative Study Regarding Redox Chemistry. *Angew. Chem. Int. Ed.* **2013**, *52* (10), 2972–2976. https://doi.org/10.1002/anie.201209329.

(17) Frisch, M. J.; Trucks, G. W.; Schlegel, H. B.; Scuseria, G. E.; Robb, M. A.; Cheeseman, J. R.; Scalmani, G.; Barone, V.; Petersson, G. A.; et al. *Gaussian 09, Gaussian, Inc.: Wallingsford, CT* **2009**.

(18) Grimme, S.; Antony, J.; Ehrlich, S.; Krieg, H. A Consistent and Accurate *Ab Initio* Parametrization of Density Functional Dispersion Correction (DFT-D) for the 94 Elements H-Pu. *J. Chem. Phys.* **2010**, *132* (15), 154104. https://doi.org/10.1063/1.3382344.

(19) Weigend, F.; Ahlrichs, R. Balanced Basis Sets of Split Valence, Triple Zeta Valence and Quadruple Zeta Valence Quality for H to Rn: Design and Assessment of Accuracy. *Phys. Chem. Chem. Phys.* **2005**, *7* (18), 3297–3305. https://doi.org/10.1039/b508541a.

(20) Jensen, F. Segmented Contracted Basis Sets Optimized for Nuclear Magnetic Shielding. *J. Chem. Theory Comput.* **2015**, *11* (1), 132–138. https://doi.org/10.1021/ct5009526.
(21) Payard, P.-A.; Perego, L. A.; Grimaud, L.; Ciofini, I. A DFT Protocol for the Prediction of ^{31} P NMR Chemical Shifts of Phosphine Ligands in First-Row Transition-Metal Complexes. *Organometallics* **2020**, *39* (17), 3121–3130. https://doi.org/10.1021/acs.organomet.0c00309.
(22) Álvarez-Moreno, M.; De Graaf, C.; López, N.; Maseras, F.; Poblet, J. M.; Bo, C. Managing the Computational Chemistry Big Data Problem: The **ioChem-BD** Platform. *J. Chem. Inf. Model.* **2015**, *55* (1), 95–103. https://doi.org/10.1021/ci500593j.
(23) K. Latypov, S.; M. Polyancev, F.; G. Yakhvarov, D.; G. Sinyashin, O. Quantum Chemical Calculations of 31 P NMR Chemical Shifts: Scopes and Limitations. *Phys. Chem. Chem. Phys.* **2015**, *17* (10), 6976–6987. https://doi.org/10.1039/C5CP00240K.
(24) Zumbulyadis, N.; Dailey, B. P. The 1H and 31P N.M.R. Spectra of Phosphine in Isotropic and Nematic Solvents. *Mol. Phys.* **1974**, *27* (3), 633–640. https://doi.org/10.1080/00268977400100551.
(25) Van Wazer, J. R.; Callis, C. F.; Shoolery, J. N.; Jones, R. C. Principles of Phosphorus Chemistry. II. Nuclear Magnetic Resonance Measurements1. *J. Am. Chem. Soc.* **1956**, *78* (22), 5715–5726. https://doi.org/10.1021/ja01603a002.
(26) Fischer, M.; Roy, M. M. D.; Wales, L. L.; Ellwanger, M. A.; Heilmann, A.; Aldridge, S. Structural Snapshots in Reversible Phosphinidene Transfer: Synthetic, Structural, and Reaction Chemistry of a Sn═P Double Bond. *J. Am. Chem. Soc.* **2022**, *144* (20), 8908–8913. https://doi.org/10.1021/jacs.2c03302.
(27) Wüllen, C. van. A Comparison of Density Functional Methods for the Calculation of Phosphorus-31 NMR Chemical Shifts. *Phys. Chem. Chem. Phys.* **2000**, *2* (10), 2137–2144. https://doi.org/10.1039/B000461H.
(28) Cicač-Hudi, M.; H. Schlindwein, S.; M. Feil, C.; Nieger, M.; Gudat, D. Isolable N-Heterocyclic Carbene Adducts of the Elusive Diiodophosphine. *Chem. Commun.* **2018**, *54* (55), 7645–7648. https://doi.org/10.1039/C8CC03972K.
(29) Schleyer, P. von R.; Manoharan, M.; Wang, Z.-X.; Kiran, B.; Jiao, H.; Puchta, R.; van Eikema Hommes, N. J. R. Dissected Nucleus-Independent Chemical Shift Analysis of π-Aromaticity and Antiaromaticity. *Org. Lett.* **2001**, *3* (16), 2465–2468. https://doi.org/10.1021/ol016217v.
(30) Schleyer, P. von R.; Maerker, C.; Dransfeld, A.; Jiao, H.; van Eikema Hommes, N. J. R. Nucleus-Independent Chemical Shifts: A Simple and Efficient Aromaticity Probe. *J. Am. Chem. Soc.* **1996**, *118* (26), 6317–6318. https://doi.org/10.1021/ja960582d.
(31) Adamo, C.; Barone, V. Toward Reliable Density Functional Methods without Adjustable Parameters: The PBE0 Model. *J. Chem. Phys.* **1999**, *110* (13), 6158–6170. https://doi.org/10.1063/1.478522.
(32) Caldeweyher, E.; Ehlert, S.; Hansen, A.; Neugebauer, H.; Spicher, S.; Bannwarth, C.; Grimme, S. A Generally Applicable Atomic-Charge Dependent London Dispersion Correction. *J. Chem. Phys.* **2019**, *150* (15), 154122. https://doi.org/10.1063/1.5090222.
(33) Staroverov, V. N.; Scuseria, G. E.; Tao, J.; Perdew, J. P. Comparative Assessment of a New Nonempirical Density Functional: Molecules and Hydrogen-Bonded Complexes. *J. Chem. Phys.* **2003**, *119* (23), 12129–12137. https://doi.org/10.1063/1.1626543.
(34) Rappoport, D.; Furche, F. Property-Optimized Gaussian Basis Sets for Molecular Response Calculations. *J. Chem. Phys.* **2010**, *133* (13), 134105. https://doi.org/10.1063/1.3484283.
(35) Marenich, A. V.; Cramer, C. J.; Truhlar, D. G. Universal Solvation Model Based on Solute Electron Density and on a Continuum Model of the Solvent Defined by the Bulk Dielectric Constant and Atomic Surface Tensions. *J. Phys. Chem. B* **2009**, *113* (18), 6378–6396. https://doi.org/10.1021/jp810292n.
(36) Meurer, F.; Kleemiss, F.; Riesinger, C.; Balázs, G.; Vuković, V.; Shenderovich, I. G.; Jelsch, C.; Bodensteiner, M. Probing the Isolobal Relation between Cp′′NiP3 and White Phosphorus by Experimental Charge Density Analysis. *Chem. Europ. J.* **2024**, *30* (19), e202303762. https://doi.org/10.1002/chem.202303762.
(37) SCALE3ABS, CrysAlisPro, Aglient Technologies Inc., Oxford, GB. **2012**.
(38) G. M. Sheldrick; SADABS, Bruker AXS, Madison, USA. **2007**.
(39) Clark, R. C.; Reid, J. S. The Analytical Calculation of Absorption in Multifaceted Crystals. *Acta Cryst. A* **1995**, *51* (6), 887–897. https://doi.org/10.1107/S0108767395007367.

(40) Dolomanov, O. V.; Bourhis, L. J.; Gildea, R. J.; Howard, J. A. K.; Puschmann, H. *OLEX2* : A Complete Structure Solution, Refinement and Analysis Program. *J. Appl. Crystallogr.* **2009**, *42* (2), 339–341. https://doi.org/10.1107/S0021889808042726.
(41) Sheldrick, G. M. *SHELXT* – Integrated Space-Group and Crystal-Structure Determination. *Acta Cryst. A* **2015**, *A71* (1), 3–8. https://doi.org/10.1107/S2053273314026370.
(42) Sheldrick, G. M. Crystal Structure Refinement with *SHELXL*. *Acta Cryst. C* **2015**, *C71* (1), 3–8. https://doi.org/10.1107/S2053229614024218.
(43) Bourhis, L. J.; Dolomanov, O. V.; Gildea, R. J.; Howard, J. A. K.; Puschmann, H. The Anatomy of a Comprehensive Constrained, Restrained Refinement Program for the Modern Computing Environment – *Olex2* Dissected. *Acta Crystallogr A Found Adv* **2015**, *71* (1), 59–75. https://doi.org/10.1107/S2053273314022207.
(44) Kleemiss, F.; Dolomanov, O. V.; Bodensteiner, M.; Peyerimhoff, N.; Midgley, L.; Bourhis, L. J.; Genoni, A.; Malaspina, L. A.; Jayatilaka, D.; Spencer, J. L.; White, F.; Grundkötter-Stock, B.; Steinhauer, S.; Lentz, D.; Puschmann, H.; Grabowsky, S. Accurate Crystal Structures and Chemical Properties from NoSpherA2. *Chem. Sci.* **2021**, *12* (5), 1675–1692. https://doi.org/10.1039/D0SC05526C.
(45) Grabowsky, S.; Genoni, A.; Bürgi, H.-B. Quantum Crystallography. *Chem. Sci.* **2017**, *8* (6), 4159–4176. https://doi.org/10.1039/C6SC05504D.
(46) Capelli, S. C.; Bürgi, H.-B.; Dittrich, B.; Grabowsky, S.; Jayatilaka, D. Hirshfeld Atom Refinement. *IUCrJ* **2014**, *1* (5), 361–379. https://doi.org/10.1107/S2052252514014845.
(47) Neese, F. Software Update: The ORCA Program System—Version 5.0. *WIREs Computational Molecular Science* **2022**, *12* (5), e1606. https://doi.org/10.1002/wcms.1606.
(48) Neese, F.; Wennmohs, F.; Becker, U.; Riplinger, C. The ORCA Quantum Chemistry Program Package. *J. Chem. Phys.* **2020**, *152* (22), 224108. https://doi.org/10.1063/5.0004608.
(49) Neese, F. The SHARK Integral Generation and Digestion System. *J. Comput. Chem.* **2023**, *44* (3), 381–396. https://doi.org/10.1002/jcc.26942.
(50) Mardirossian, N.; Head-Gordon, M. ωB97X-V: A 10-Parameter, Range-Separated Hybrid, Generalized Gradient Approximation Density Functional with Nonlocal Correlation, Designed by a Survival-of-the-Fittest Strategy. *Phys. Chem. Chem. Phys.* **2014**, *16* (21), 9904–9924. https://doi.org/10.1039/C3CP54374A.
(51) Weigend, F.; Ahlrichs, R. Balanced Basis Sets of Split Valence, Triple Zeta Valence and Quadruple Zeta Valence Quality for H to Rn: Design and Assessment of Accuracy. *Phys. Chem. Chem. Phys.* **2005**, *7* (18), 3297–3305. https://doi.org/10.1039/B508541A.
(52) Brüx, D.; Meurer, F.; Kleemiss, F. Benchmarking Crystal Structure Refinement: A Systematic Study on Hirshfeld Atom Refinement. *Struct. Dyn.* **2025**, *12* (5), 054101. https://doi.org/10.1063/4.0000774.
(53) Riesinger, C.; Meurer, F.; Zimmermann, L.; Dütsch, L.; Scheer, M. Strain-Release Driven Arsenium Ion Bond Insertion. *Angew. Chem. Int. Ed.* **2025**, *64* (35), e202510186. https://doi.org/10.1002/anie.202510186.
(54) Xiong, Y.; Yao, S.; Brym, M.; Driess, M. Consecutive Insertion of a Silylene into the P4 Tetrahedron: Facile Access to Strained SiP_4 and Si_2P_4 Cage Compounds. *Angew. Chem. Int. Ed.* **2007**, *46* (24), 4511–4513. https://doi.org/10.1002/anie.200701203.
